# Quantification of the Effects of Droughts on Daily Mortality in Spain at Different Timescales at Regional and National Levels: A Meta-Analysis

**DOI:** 10.3390/ijerph17176114

**Published:** 2020-08-22

**Authors:** Coral Salvador, Raquel Nieto, Cristina Linares, Julio Díaz, Luis Gimeno

**Affiliations:** 1 EPhysLab (Environmental Physics Laboratory), CIM-UVIGO, Universidade de Vigo, 32004 Ourense, Spain; rnieto@uvigo.es (R.N.); l.gimeno@uvigo.es (L.G.); 2 Department of Epidemiology and Biostatistics, Carlos III National Institute of Health (Instituto de Salud Carlos III/ISCIII), National School of Public Health, 28029 Madrid, Spain; clinares@isciii.es (C.L.); j.diaz@isciii.es (J.D.)

**Keywords:** drought, standardized precipitation evapotranspiration index, standardized precipitation index, meta-analysis, mortality, Spain

## Abstract

A performance assessment of two different indices (the Standardized Precipitation Index (SPI) and the Standardized Precipitation Evapotranspiration Index (SPEI)) for monitoring short-term and short–medium-term drought impacts on daily specific-cause mortality in Spain was conducted. To achieve a comprehensive, nationwide view, a meta-analysis was performed using a combination of provincial relative risks (RRs). Moreover, the subdivisions of Spain based on administrative, climatic, and demographic criteria to obtain the measures of combined risks were also taken into account. The results of the SPEI and SPI calculated at the same timescale were similar. Both showed that longer drought events produced greater RR values, for respiratory mortality. However, at the local administrative level, Galicia, Castilla-y-Leon, and Extremadura showed the greatest risk of daily mortality associated with drought episodes, with Andalucía, País Vasco, and other communities being notably impacted. Based on climatic regionalization, Northwest, Central, and Southern Spain were the regions most affected by different drought conditions for all analyzed causes of daily mortality, while the Mediterranean coastal region was less affected. Demographically, the regions with the highest proportion of people aged 65 years of age and over reflected the greatest risk of daily natural, circulatory, and respiratory mortality associated with drought episodes.

## 1. Introduction

Drought is a complex phenomenon that occurs due to a deficiency of precipitation over an extended period of time. It can be promoted by an increase in evapotranspiration associated with the incidence of high temperatures, strong winds, or low humidity, causing a significant hydrological imbalance [1,2]. It is among the most damaging types of natural disaster. Drought operates in different places and at different timescales, leading to notable impacts on diverse environmental and social sectors [3,4,5] with significant effects on human health, including an increased risk of morbidity and mortality [6,7,8,9,10,11]. The response of environmental systems to the occurrence of this hydrologically extreme phenomenon can vary according to the time period in which it is measured. For this reason, evaluating the performance of different types of drought indices and the sensitivity of the response to different timescales is important for predicting and quantifying drought impacts on a specific system or sector, such as human health. In this context, drought effects can be typically classified as direct, commonly referred to as the reduction of water quantity and quality, reduced crop and food production, diminution of forest productivity, reduction of soil and air quality, wildlife losses, or higher risk of heatwaves and/ or wildfires, which, in turn, lead to increased pollution due to wildfire smoke [12,13]. The consequences of these impacts are considered indirect effects, such as the reduction of income for farmers, the increased food and timber prices, migration, and most of the human health impacts [3,6,14,15].

The Iberian Peninsula in southwestern Europe has particularly suffered from long and severe periods of drought in recent decades [5], causing forest reduction, crop yield failures, and severe impacts on the economy [16]. In this area, drought is described as the main hydroclimatic hazard. According to future climate change projections, droughts in this region are likely to become more frequent and severe by the end of the 21st century [17,18]. Assessments of drought impacts at different timescales have been conducted on several systems in the Iberian Peninsula using variables such as streamflow and reservoir shortages [19,20], groundwater level [21], vegetation activity [22], crop productivity [23], forest growth [24,25], and the occurrence of wildfires and areas burned by them [26,27,28]. However, analyzing the details of the relationship between drought characteristics and health impacts using different indices and different timescales is seldom considered.

At the national level, there is only one recent study, carried out in peninsular Spain from 2000 to 2009, in which the study of the short-term effects of drought conditions on daily specific-cause mortality were measured. This was determined via the calculation of the relative risk (RR) of drought conditions, measured on a one-month timescale using the Standardized Precipitation Index (SPI; based on precipitation data) and the Standardized Precipitation Evapotranspiration Index (SPEI; which additionally incorporates temperature data for its calculation, based on the difference between precipitation and evapotranspiration potential). Daily natural (all causes except accidents), circulatory, and respiratory mortality were assessed for each province in the study area [29]. In a regional study conducted in Galicia (northwest Spain), the first evidence of the relationship between drought and daily mortality rate was gathered using the SPI and the SPEI calculated at one, three, six, and nine months of drought accumulation [30]. In general, shorter timescales (one and three months) reflected the highest drought effects on daily mortality in inland provinces, whereas for coastal provinces, a significant relationship between daily circulatory-caused mortality and drought measured was observed only at a longer timescale (9 months). Moreover, slight differences were obtained when both types of indices were compared; however, qualitatively, there was variability in the risk magnitude of different causes of deaths linked to drought conditions measured at different timescales. This fact highlights the importance of carrying out a broader analysis, at least for shorter timescales, across Spain. In turn, these studies revealed that for a considerable number of provinces drought indices calculated at short-term were indirect indicators that largely reflected the impact of drought on daily mortality through the occurrence of heatwaves and atmospheric pollution, phenomena frequently associated with this hydrological extreme.

In this context, several environmental mechanisms have been proposed to link drought episodes with health outcomes such as increased morbidity and mortality due to cardiorespiratory causes. Drought and heatwaves can contribute to a higher risk of wildfires, which can cause damaging repercussions on respiratory and circulatory systems, including mortality, due to smoke exposure [7,31,32,33]. Moreover, in a direct pathway, drought can also lead to poor air quality [13]. Meanwhile, in urban areas drought is usually correlated with the presence of resilient high atmospheric pressure systems such as persistent atmospheric blocking conditions, which frequently lead to higher concentrations of surface ozone (under increased ultraviolet irradiance) and other accumulated pollutants [34,35], with significant impacts on natural, circulatory, and respiratory causes of deaths [36]. Persistent blocking conditions can, in turn, trigger the increase of other extreme events such as severe heat and cold events [37,38,39], both with notable short-term effects on cardiovascular and respiratory systems and death [40]. Meanwhile, longer droughts could cause the exacerbation of health conditions and premature mortality through their impact on mental health. The occurrence of prolonged droughts has been associated with chronic stress, generalized anxiety, and depression [7,41], which have been linked to a higher risk of cardiovascular diseases and other disorders [42,43]. In addition, prolonged droughts could contribute to a higher risk of suicide, especially in the vulnerable populations [9,15,41]. Thus, dry regions and areas prone to drought usually present a concern for human health.

**Figure 1:**
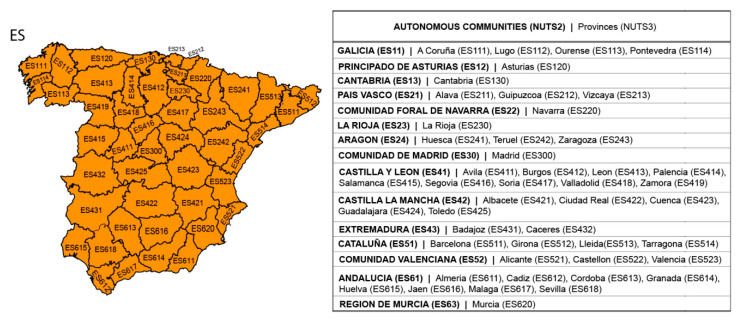
Subdivision of peninsular Spain according to the European Nomenclature of Territorial Units for Statistics (NUTS). The provincial and Autonomous Communities used correspond to levels 2 (NUTS2) and 3 (NUTS3), respectively. The NUTS3 provinces are shown on the map, with their names and the names of the corresponding NUTS2Autonomous Communities listed in the box on the right. ES = NUTS code of Spain.

According to those works previously cited, it is highlighted that more than one period of accumulation is needed to measure drought, and that to observe and compare its effects, timescales longer than one month are required. Thus, considering that daily mortality was found to be mainly influenced by shorter timescales in the regional study of Galicia, the impacts of short–medium-term droughts (i.e., those lasting around three months) on daily natural, circulatory, and respiratory mortality were evaluated at the provincial level from 2000 to 2009 in this study. The main objective was to compare different findings to reveal which drought accumulation period had greater influence on the different causes of deaths in each of the 47 peninsular provinces of Spain (Figure 1). Moreover, the combination of the provincial RRs of daily mortality linked to drought, according to broader territorial spatial groups and overall for peninsular Spain, was also considered using a meta-analysis of random effects. This allowed for attaining a comprehensive view of the effects for the entire the country.

In climatological terms and in terms of drought occurrence, the Iberian Peninsula is a region with marked spatial differences in the frequency, average duration, and magnitude of drought episodes, with strong north–south and west–east gradients. In general, drought frequency is higher in the North than in the South, whereas the average duration and magnitude of droughts are much higher in the Central and Southern regions [44,45]. Following Vicente-Serrano (2006), peninsular Spain was divided according to six different drought spatial patterns on shorter timescales (one and three months) based on a principal component analysis [46]. Monjo et al. (2020) recently characterized meteorological droughts from south to north as a function of the longitude of alternating dry and wet periods. The south is where long dry spells alternate with short wet events, and the North is where median dry spells alternate with longer wet spells [47]. Furthermore, Northwest Spain shows the highest frequency of flash droughts (i.e., drought events which develop rapidly). However, these events show a decreasing trend in this region over the last six decades. In contrast, the Central and Southern regions show a lower frequency, but positive trends [48]. On the other hand, the spatial distribution of drought in the Iberian Peninsula varies in function according to the timescale used for its measurement, i.e., its complexity increases on longer timescales [46].

Furthermore, it is known that the distribution of demography in terms of age exacerbates the different causes of mortality and morbidity rates, including those linked to extreme climatic events such as droughts [7]. Spain presents a regressive or bulb-shaped population pyramid. The Northwest territories are where the highest proportion of aging people are concentrated [49].

Thus, to obtain a broader measure of the relative risk (RR) at different levels through the combination of provincial RR values according to different criteria, several meta-analyses were conducted in this study based on three different motivations with diverse aims: (1) To develop a perspective on the effects of drought at a higher administrative division than provinces, namely Autonomous Communities, which are needed as the basis for future decision making for public health policy across the country; (2) with a climatological aim, to quantify the combined relative risks of daily mortality taking into account the drought spatial patterns [46]; and (3) to generate evidence of the influence of age on mortality due to droughts for those groups of provinces with a higher proportion of people aged 65 and over. The main objective of this study was to quantify the impact of droughts on daily mortality in Spain at different territorial levels and sectors. This is the first step in economically establishing the effect that this hydrological extreme has on population health in terms of mortality. In this aspect, economic quantification of drought effects is crucial in deciding and prioritizing the actions by the administrations. The approach of this study may serve as guidance to provincial, autonomic, and national health systems and other relevant authorities to implement effective strategies for better drought management, obtain better preparation, and create early responses to mitigate the damaging effects of drought on human health as well as reduce the vulnerability in a high-risk population group like the elderly.

## 2. Materials and Methods

### 2.1. Variables

The dependent variables used corresponded to daily specific-cause mortality classified according to the 10th revision of the International Statistical Classification of Diseases and Related Health Problems (ICD10). Particularly, the daily natural (ICD10: A00-R99), circulatory (ICD10:I00-I99), and respiratory (ICD10: J00-J99) mortality data for each province of peninsular Spain (Figure 1) from 2000 to 2009 were used. Mortality is represented by the number of deaths in the capital city and towns with over 10,000 inhabitants, and it was provided by the National Institute of Statistics (INE) of Spain. No mortality data were available for Palencia province (NUT3 ES414) or Madrid (NUT3 ES300) for 2000.

The independent variables used were two multi-scalar drought meteorological indices: The SPI [50] and the SPEI [51]. Drought conditions were identified by both indices as values less than 0; the change to a positive value indicated the end of the drought event. Data for both drought indices for one month (short-term) and three months (short–medium term) of accumulation (denoted as SPI-1/SPEI-1 and SPI-3/SPEI-3, respectively) were obtained from the Spanish National Research Council (CSIC) website http://monitordesequia.csic.es [16], at a weekly resolution. These drought series are based on a standard normal variable; therefore, both can be compared in time and space across different timescales [51]. Following the methodology developed in previous studies [29,30], we constructed daily series assuming the same conditions for each seven-day interval. Moreover, other control variables such as the trend (considering n1=1 for the first day of the series, n1=2 for the second day, and so one to the end of the series), the autoregressive nature of the dependent variable, and the seasonality (through sine and cosine functions for the annual (360-day), half-year (180-day), four-month (120-day), quarterly (90-day), and bimonthly (60-day) periodicities were also taken into account.

### 2.2. Statistical Analysis

The purpose of this meta-analysis study was to develop a broader view on the RRs of daily specific-cause mortality associated with different drought conditions beyond a provincial (i.e., relatively small) area effect and to compare the findings obtained to the use of different timescales for SPEI and SPI. The individual provincial RR results due to SPI-1/SPEI-1 for each province from 2000 to 2009 were published by Salvador et al. (2020) [29]. Applying the same methodology, the RRs of mortality linked to drought over the short–medium term, measured by SPI-3/SPEI-3, were quantified based on generalized linear models with the Poisson regression link. As SPEI and SPI have negative values for the drought periods, the negative coefficients obtained in the Poisson models were those that were taken into consideration. Then, from the magnitude of these coefficients in absolute value, the RR of daily mortality was calculated for each unit of increment of both drought indices over the short and short–medium timescales. Thus, the RR shows the increase in daily mortality risk associated with the increase in drought severity in the exposed population relative to the unexposed population.

To determine the significant variables, the “backward-step procedure” was carried out, beginning with the model that included all explanatory variables (independent and control variables), removing gradually and individually those that displayed least statistical significance until a model with only statistically significant variables at *p* < 0.05 was obtained. Single models for independent SPEI-3/SPI-3 series and for the different causes of daily mortality were conducted.

Moreover, the RRs for each province yielded by the Poisson models were combined by means of a meta-analysis of random effects to obtain the overall value for peninsular Spain. A measure of the RRs (95% CI) according to the different criteria of spatial grouping (administrative, climatic, or demographic) was also obtained. This method incorporated an estimation of variability [52], namely, inter-studio heterogeneity.

## 3. Results

### 3.1. RR of Mortality Associated with Droughts by Spanish Administrative Division

The RR values of daily mortality associated with the occurrence of drought, as measured by the different timescales for peninsular Spain as a whole from 2000 to 2009, are shown in Table 1. A significant relationship between drought episodes and all analyzed causes of daily mortality, independent of the type of index (SPEI or SPI) and timescale (one or three months), was found. The results obtained using both indices at the same timescale were generally very similar; moreover, differences in the magnitude of daily mortality risks associated with the use of the different timescales for SPEI and SPI were found, although the differences were not significant. The highest impact was found for respiratory deaths, especially from short–medium-term drought events. Meanwhile, for circulatory deaths, the RR values were similar under different drought conditions, reflecting SPI-1, a higher RR value. In the case of natural deaths, greater RR values were obtained for short–medium-term droughts. Figures S1–S9 in the Supplementary Material list all RR values with their respective 95% confidence intervals.

**Figure 2:**
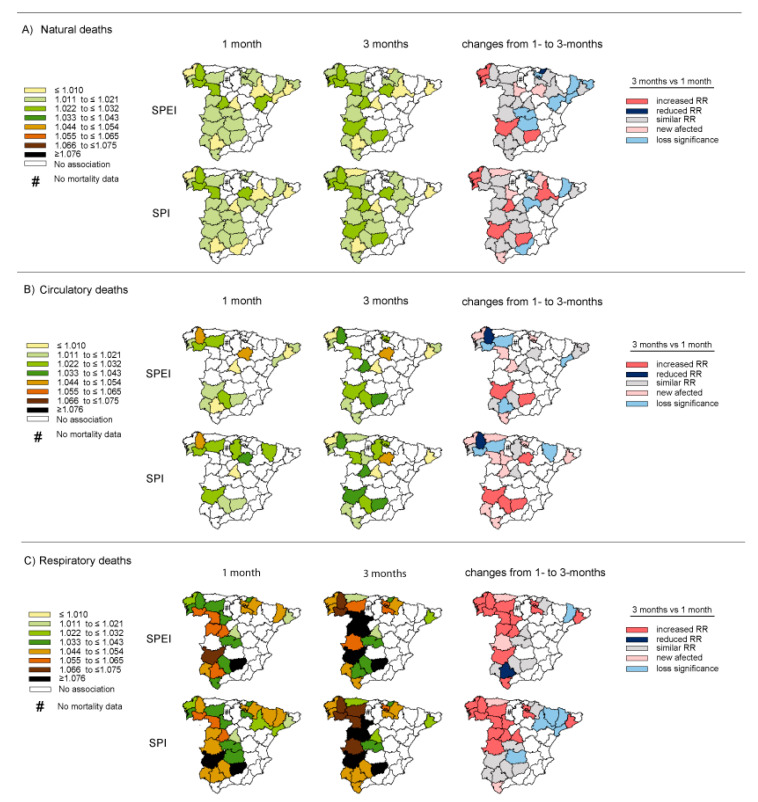
Relative risks (RRs) (95% CI) of daily mortality linked to drought conditions. Comparison of the provincial RRs of daily mortality associated with drought measured at different timescales. RR values for daily (**A**) natural, (**B**) circulatory, and (**C**) respiratory mortality linked to droughts measured by the Standardized Precipitation Evapotranspiration Index (SPEI) and the Standardized Precipitation Index (SPI) calculated for short (one month, left-hand column) and short–medium (three months, center column) timescales. The right-hand column provides a comparison of the differences among them. RR values for SPEI-1 and SPI-1 weretaken from Salvador et al. (2020) [29]. Complete statistical information is provided in the Supplementary Material (Figures S1–S3).

Although the results obtained at a nationwide level show an overall perspective and reflect the main evidence, evaluation at the provincial and Autonomous Community levels identified additional differences and heterogeneity throughout the country. As mentioned previously, the RRs for each province for one month of accumulation were calculated by Salvador et al. (2020) [29]. Although the overall RRs’ values at three months were similar (SPEI-3 vs. SPI-3), when a provincial analysis was conducted for three months of accumulation, some differences were found, namely that SPI was generally slightly better for detecting mortality risk for several provinces than SPEI. Figure 2 shows a comparison of the RRs for daily natural, circulatory, and respiratory mortality associated with drought conditions measured by SPEI/SPI and calculated at one and three months of accumulation. The highest impact of drought observed was on respiratory deaths. Moreover, in the majority of cases, the RR values were higher (or similar) under short–medium-term drought conditions than under short-term conditions. (Although the difference was not significant, this increase in risk is remarkable, especially for respiratory mortality in those provinces located in Western Spain). However, there were particularities where daily mortality was mainly manifested by one type of index (or timescale) rather than the other. For instance, in provinces located in Northeast Spain, mortality was mainly influenced by short-term drought events because the significant relationship between drought and mortality was lost when this hydrological extreme was measured over the short–medium term. Meanwhile, it should be noted that although the overall RR value of natural and respiratory deaths was qualitatively greater under longer drought events, the number of statistically significant provinces was lower with the use of SPEI-3 for natural deaths and SPI-3 for respiratory deaths in comparison with that obtained via the use of SPEI-1 and SPI-1, respectively. However, in the case of circulatory mortality, an increase in the number of affected provinces was found, particularly for SPI-3.

As Spain is administratively divided into intermediate territorial areas termed Autonomous Communities, the RRs levels were obtained for each. This will provide useful information for public health administrations as it is extremely important to know the mortality risk factors of the population in order to implement action plans and make appropriate management decisions.

**Figure 3:**
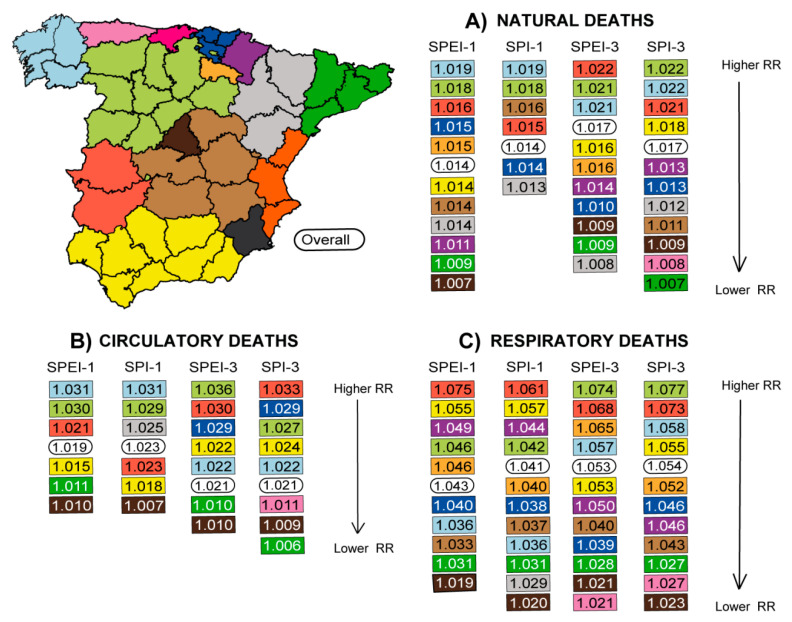
Combined provincial relative risk (RR) values of daily specific-cause mortality associated with drought for each Autonomous Community and overall for peninsular Spain from 2000 to 2009, as measured by the Standardized Precipitation Evapotranspiration Index (SPEI) and the Standardized Precipitation Index (SPI) calculated for short and short–medium timescales (one and three months of accumulation, respectively). Complete statistical information is provided in the Supplementary Material (Figures S1–S3).

As the management of public health has been delegated by the National Government to the Autonomous Communities, Figure 3 shows the combined provincial RR values for daily natural, circulatory, and respiratory mortality linked to different drought conditions at the Autonomous Community level. The main findings indicate that, in general, Galicia, Castilla-y-Leon, and Extremadura (Northwest and West Iberian Peninsula) show the greatest risk of daily mortality linked to drought conditions. All three have RR values above the overall value for the country, except Galicia for respiratory deaths when drought was measured over the short-term. Particularly, in Castilla-y-Leon, a significantly greater impact was observed for short–medium-term droughts than for short-term droughts, as measured by SPI (SPI-3 vs. SPI-1, Figure S3 in the Supplementary Material). Other autonomous communities such as Pais Vasco (principally for circulatory mortality associated with drought episodes measured in the short–medium term), Andalucia and Navarra (for respiratory deaths), and La Rioja (for respiratory mortality linked to short–medium-term droughts) were also notably impacted. In contrast, the lowest RR values due to any analyzed cause of death were found in the Comunidad de Madrid, Cataluña, and Principado de Asturias. Meanwhile, drought events were not found to have any influence on daily mortality in Cantabria, Murcia, or Comunidad Valenciana on the Mediterranean coast. It should be noted that there were differences (although not significant) in the magnitude of the risks in some of the Autonomous Communities according to the index, timescale, and cause of death analyzed, particularly for circulatory and respiratory deaths. For instance, in Galicia, shorter droughts reflected a higher impact on circulatory mortality than short–medium-term ones, contrary to what was observed for respiratory deaths. In Extremadura, the shortest timescale of SPEI (SPEI-1) showed a higher risk for respiratory deaths than SPEI-3. Conversely, the SPI-3 calculated greater impact on respiratory mortality than SPI-1 for this region.

### 3.2. RR of Daily Mortality Associated with Climatological Spatial Patterns of Droughts

**Figure 4:**
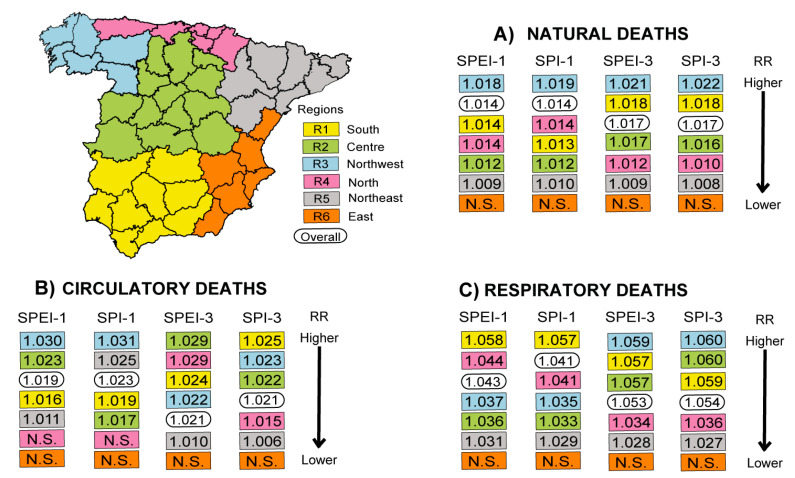
Combined provincial relative risk (RR) values of daily specific-cause mortality associated with drought for different spatial patterns of drought distribution and overall for peninsular Spain from 2000 to 2009, as measured by the Standardized Precipitation Evapotranspiration Index (SPEI) and the Standardized Precipitation Index (SPI) obtained for short and short—medium timescales (one and three months of accumulation, respectively). Complete statistical information is provided in the Supplementary Material (Figures S4–S6).

As previously mentioned, the behavior of droughts varies according to region. For this reason, the known spatial distribution pattern of drought indices across Spain [44,46] were additionally analyzed to produce a climatological point of view of the RRs of daily mortality linked to drought. Figure 4 shows the combined provincial RR values of daily specific-cause mortality linked to the occurrence of droughts grouped according to the spatial distribution of drought indices described by Vicente-Serrano (2006) and obtained at shorter timescales [46]. Figures S4–S6, in the Supplementary Material show the combined RRs of the different causes of daily mortality associated with drought by the climatic regionalization with their respective 95% confidence intervals. The provinces were grouped into six regions, which are the most similar to the six spatial patterns of drought across Spain. The main findings demonstrated that droughts had a significant influence on daily mortality in all regions except East Spain, where there was no significant relationship between this phenomenon and any cause of mortality (no province in this region had a significant RR value). In contrast, Northwest, Central, and South were the most affected regions under the different drought conditions. That is, in qualitative terms, the Northwest had a higher RR value than the RR value for peninsular Spain for natural and circulatory causes of mortality and the South had a higher value than that of peninsular Spain for respiratory deaths.

For natural deaths, the highest impact was observed in the Northwest (especially remarkable for three months of accumulation) and the South using both types of indices, followed by Central when droughts were measured at the short–medium term. Contrarily, the Northeast was the region least affected. For daily circulatory-caused mortality, the Northwest (mainly for short-term drought events) as well as Central and Southern regions (mainly for short–medium drought conditions) were the territories most affected. The lowest influence of drought on daily circulatory deaths was evidenced in the Northeast and the North, except when drought episodes were measured by SPI-1 and SPEI-3, respectively. Meanwhile, the greatest RR values of daily respiratory mortality measured by SPEI-1 and SPI-1 were found in the South, North, and Northwest, whereas they were found in the Northwest, South, and Central regions for SPEI-3 and SPI-3. In addition, the RRs of respiratory mortality associated with drought episodes in the Northwest were significantly higher for short–medium-term droughts measured by both indices than for short-term droughts (Figure S6 in the Supplementary Material). Again, Northeast Spain was the region least impacted by droughts in terms of respiratory mortality risks.

### 3.3. RR of Daily Mortality Associated with Droughts Based on Demography

**Figure 5:**
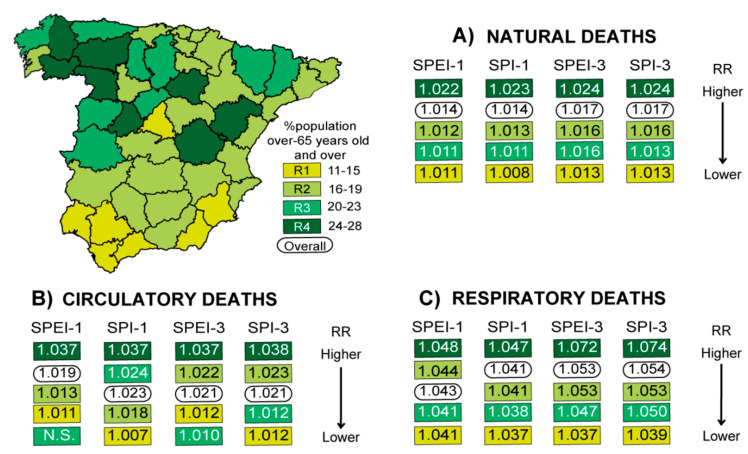
Combined provincial relative risk (RR) values of daily specific-cause mortality associated with drought in different territories based on the proportion of people aged 65 years and over from 2000 to 2009, measured by the Standardized Precipitation Evapotranspiration Index (SPEI) and the Standardized Precipitation Index (SPI) obtained for short and short–medium timescales (one and three months of accumulation, respectively). Complete statistical information is provided in the Supplementary Material (Figures S7–S9).

Annual demographic data of the population of each province from 2000 to 2009 was obtained from the INE [53]. Each province was categorized into one of the following four groups according to whether the population was aged 65 years old and over represented: (1) 11–15% of the population (8 provinces), (2) 16–19% of the population (23 provinces), (3) 20–23% of the population (9 provinces), or (4) 24–28% of the population (7 provinces; Figure 5). The combined RRs of the different analyzed causes of mortality measured by both SPEI and SPI at short and short–medium terms were then obtained according to the above defined groups (Figure 5 and Figures S7–S9). The main findings obtained indicated that the group of provinces with the highest proportion of people aged 65 years old and over had the greatest risk of mortality linked to the occurrence of drought episodes both at short and short–medium timescales. The impacts were found to be higher for short–medium-term drought events (the difference being significant at the 95% level of confidence using SPEI and with a significant trend using SPI for respiratory mortality). Moreover, in the areas with the lowest proportion of people aged 65 years old and over, the lowest RR values were generally observed (with some exceptions, principally for circulatory deaths). Those provinces with the lowest proportion of people aged 65 years old and over (e.g., Madrid, Murcia, Malaga) were the least affected. Meanwhile, the results obtained through the use of SPEI and SPI obtained at the same timescale were very similar. It is notable that some of the most drought-prone regions, which show the highest mortality, also coincide with the regions with aging populations (e.g., Northwest Spain, where the majority of provinces have a high proportion of people over the age of 65). East Spain was the climatological region least affected in terms of mortality, and with fewer people aged 65 years or older. However, Southern Spain as a whole, which also contained a majority of provinces with the lowest proportion of people aged 65 years or older, was a climatological region notably impacted by drought on mortality. It should be noted that this analysis considered age to be a factor that can influence drought risk on population health; thus, based on this first approach, more exhaustive and direct analyses that take into account different age groups should be conducted in the future.

## 4. Discussion

This study found that in peninsular Spain, drought events are associated with daily natural, circulatory, and respiratory causes of mortality, with the highest impact found on respiratory deaths. In the majority of cases, short–medium-term drought events (measured by SPEI-3and SPI-3) reflected higher (or similar) impacts than short-term ones (using SPEI-1and SPI-1).Given that drought effects on cardiorespiratory conditions are principally indirect through mechanisms linked to extreme temperatures and reduced air quality (either directly, associated with persistent atmospheric high pressure systems, or indirectly through a higher risk of wildfires) [6,10,29,54,55], these phenomena could been exacerbated by longer drought conditions, reflecting greater impact on daily mortality than short-term droughts. In this context droughts can occur combined with other environmental events, and these phenomena can mutually strengthen one another through positive feedbacks [56,57,58] and cause notable impacts on health [35,59]. It has been described that high temperatures, dust, and poor air quality are common during drought conditions [60]. Moreover, warmer temperatures and ultraviolet radiation have been also often associated with increases in ground-level ozone [10,59], which, in consequence, could highly compromise cardiovascular and pulmonary health in the exposed population [15,61,62,63,64]. However, it should be noted that all these hazards can also occur independently.

Meanwhile, the performance assessment of different drought indices calculated for different timescales to identify and quantify health effects was for first time considered nationwide. The main results of this study indicated that the overall RRs of the different causes of mortality analyzed for peninsular Spain overall were similar when SPEI and SPI, calculated at the same timescale, were compared. This suggests that a deficiency in precipitation was the most influential variable in the estimation of drought effects on the different causes of deaths, and the atmospheric evaporative demand had a minor role (the differences between the indices are reflected by the potential evapotranspiration). Therefore, for this region as a whole, either index can be used to measure links with the risk in daily mortality. Using either index, regional differences were observed, which could be associated with the specific characteristics of each region, which are characterized by particular climatic and environmental conditions. This shows that an assessment at different regional levels is essential for this type of study. Moreover, although in this study we did not conduct an independent analysis for summer and winter periods, the season where drought principally occurs in a determinate territory could influence the fact that SPI (which considers only precipitation for its calculation) or SPEI (which additionally takes into account the influence of temperature) can be a better proxy to reflect the different risks of daily mortality in specific cases.

The main findings obtained through the analysis from a public health point of view (at the administrative level) suggest that the Autonomous Communities located in Northwest and West Spain showed the greatest risk of daily mortality associated with the occurrence of drought conditions, followed by other Autonomous Communities such as Andalucia or Pais Vasco, which were also notably impacted. Based on the climatic regionalization of peninsular Spain, the Northwest, Central, and Southern regions were most affected in contrast to that observed in the Mediterranean coastal regions. In this aspect, several factors could influence regional differences of daily mortality effects attributable to droughts, due to the risk depending on a combination of the exposure to the drought events and the social vulnerability [65,66]. Differences in drought characteristics across peninsular Spain could account for the higher impact of drought on mortality in the previously cited regions. Particularly, a recent study highlights that Southern and Central Spain have a higher probability of extreme drought events’ occurrence in comparison with the Northern and Eastern regions [45]. In this way, a higher severity of droughts could influence a greater risk on health, as it was shown, for instance, in a study conducted in the USA [9] taking into account different severity of drought conditions in which, during high severity of worsening drought periods, there was a significant increase of mortality in older adults. Meanwhile, in the north of Spain, droughts occur more frequently than in the south [44]. In this case, if there is an inadequate recovery time for the population following a drought episode, as well as a deficiency of adaptation measures, drought could be a factor of vulnerability increasing the susceptibility of the population for subsequent events and rising the health risks associated with them [55]. Differences in the population vulnerability could also contribute to differences in mortality risks attributable to drought, which largely depends on demographic and economic factors, health status, adaptive capacity of the population, or social behavior [55,65,66,67].

Meanwhile, from a demographic point of view, the region that included those provinces with the highest proportion of people aged 65 years and over showed the greatest risk of daily natural, circulatory, and respiratory mortality associated with the occurrence of this hydroclimatic hazard. This may be linked to the fact that the elderly are particularly vulnerable to extreme climatic phenomena due to their high prevalence of chronic health conditions, reduced mobility, and their decreased ability to respond to environmental stressors, which make them a group at greater risk of morbidity and mortality associated with the occurrence of these hazards. Moreover, gender is another factor that could greatly influence the population vulnerability to extreme climatic events, as reports the Intergovernmental Panel on Climate Change [7]. However, in this study, this variable could not be additionally controlled.

Given that the risks are the result of a complex interplay between these different factors, the combination of them should be taken into account, even more when, in many cases, the results observed for Spain, considering the administrative, climatic, and demographic approach, are not always coincident, e.g., in Southern Spain. Meanwhile, in Galicia and Castilla-y-Leon, two Autonomous Communities located within a climatic region highly impacted by drought in terms of daily mortality, are also the most impacted taking into account the demographic criteria of analysis and both are composed of provinces with the highest proportion of elderly people. Whereas eastern Spain, the region with a low proportion of elderly people, was the climatic region least affected by droughts in terms of mortality. Other variables, like the economy and government politics of each Autonomous Community, resources’ availability, and the preparedness of autonomic public health systems and environmental territorial services against the effects of drought and other climate-related extreme phenomena that can be concurrent with it, could also influence the differences in health repercussions associated with drought episodes. Moreover, the management of water resources in the society, environmental awareness and degradation, urbanization, the use of water and land, population growth, and migration trends are also important vulnerability factors to drought [65]. On the other hand, the fact that this study considered an indirect measure of age, considering an overall threshold for the aging population (65 years old), highlights the need to conduct further assessments taking into account more and different age groups.

## 5. Conclusions

This study highlights the importance to implement individual and coordinated action plans based on specific characteristics of each region due to the heterogeneity of the results, and to conduct cooperative strategies at different levels and sectors (e.g., public health, environmental, scientific, socioeconomic, and political), including integrative approaches for better drought management, preparedness, and adaptation, with the aim to mitigate the drought risks on health, and to reduce the vulnerability, especially among the most-at-risk population [65,68].

## Figures and Tables

**Table 1:** Overall relative risks (RRs) and their respective 95% confidence intervals of daily specific-cause mortality associated with drought in peninsular Spain at national level from 2000 to 2009. Standardized Precipitation Evapotranspiration Index (SPEI) and the Standardized Precipitation Index (SPI) measured at one and three months of accumulation (SPEI-1/SPI-1 and SPEI-3/SPI-3, respectively).

Causes of Mortality	SPEI-1	SPI-1	SPEI-3	SPI-3
Natural deaths	1.014 (1.012, 1.016)	1.014 (1.011, 1.016)	1.017 (1.014, 1.020)	1.017 (1.014, 1.020)
Circulatory deaths	1.019 (1.014, 1.025)	1.023 (1.016, 1.031)	1.021 (1.015, 1.027)	1.021 (1.015, 1.026)
Respiratory deaths	1.043 (1.035, 1.052)	1.041 (1.034, 1.048)	1.053 (1.043, 1.062)	1.054 (1.045, 1.063)

## Supplementary Materials

The following are available online at https://www.mdpi.com/1660-4601/17/17/6114/s1, Figure S1: Forest plots of the relative risk (RR) values of daily natural mortality associated with droughts by the administrative subdivisions of peninsular Spain, i.e., the Autonomous Communities and their provinces; Figure S2: Forest plots of the relative risk (RR) values of daily circulatory mortality associated with droughts by the administrative subdivisions of peninsular Spain, i.e., the Autonomous Communities and their provinces; Figure S3: Forest plots of the relative risk (RR) values of daily respiratory mortality associated with droughts by the administrative subdivisions of peninsular Spain, i.e., the Autonomous Communities and their provinces.; Figure S4: Forest plots of the relative risks (RR) values of daily natural mortality associated with droughts by the climatic regionalization; Figure S5: Forest plots of the relative risks (RR) values of daily circulatory mortality associated with droughts by the climatic regionalization; Figure S6: Forest plots of the relative risks (RR) values of daily respiratory mortality associated with droughts by the climatic regionalization; Figure S7: Forest plots of the relative risks (RR) values of daily natural mortality associated with droughts for provincial groups based on the proportion of elderly population in peninsular Spain; Figure S8: Forest plots of the relative risks (RR) values of daily circulatory mortality associated with droughts for provincial groups based on the proportion of elderly population in peninsular Spain; Figure S9: Forest plots of the relative risks (RR) values of daily respiratory mortality associated with droughts for provincial groups based on the proportion of elderly population in peninsular Spain.
