# Supplementary material for: Quantification of the Effects of Droughts on Daily Mortality in Spain at Different Timescales at Regional and National Levels: A Meta-Analysis
Source: Int J Environ Res Public Health. 2020 Aug 22;17(17):6114. doi: 10.3390/ijerph17176114 (PMC7504151; doi:10.3390/ijerph17176114)
Supplement: Supplementary file 1 [file ijerph-17-06114-s001.pdf]

International Journal of
Environmental Research
and Public Health

MDPI

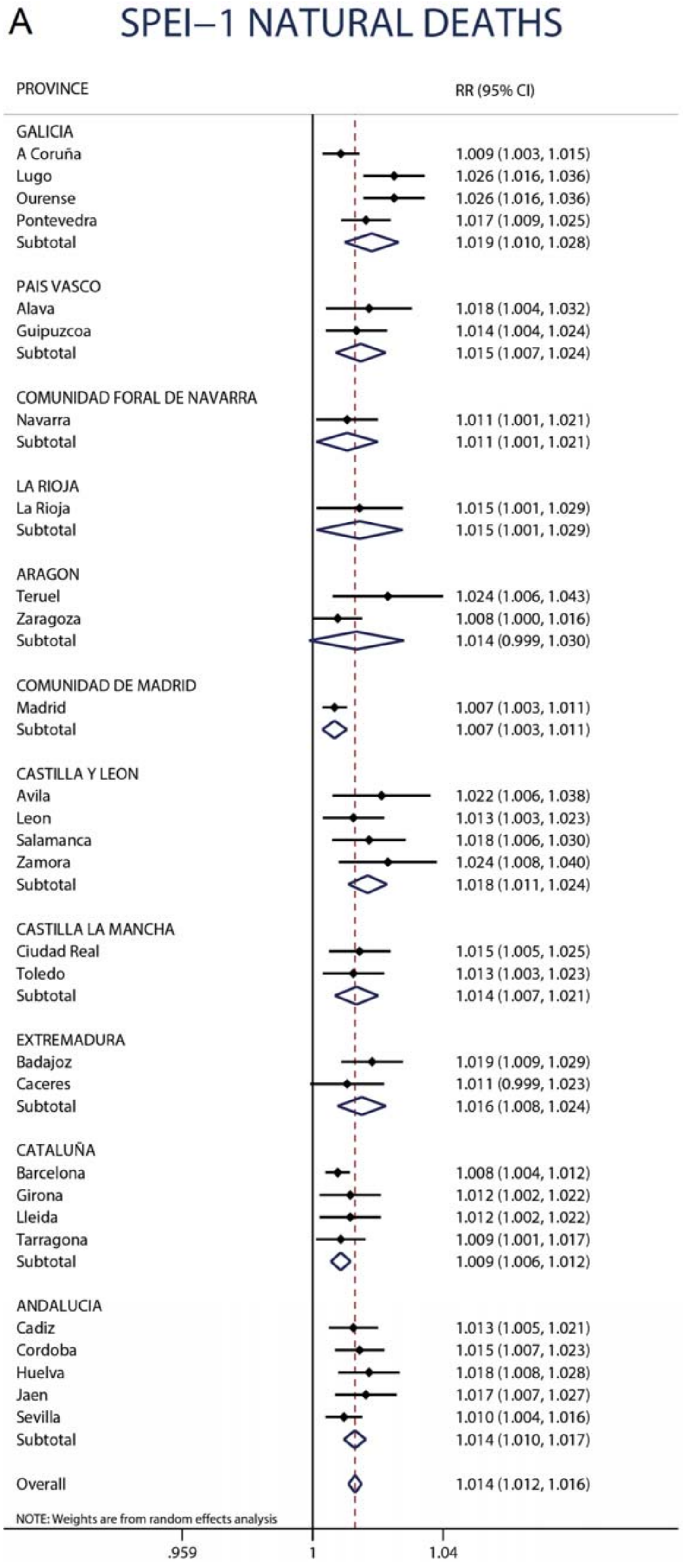

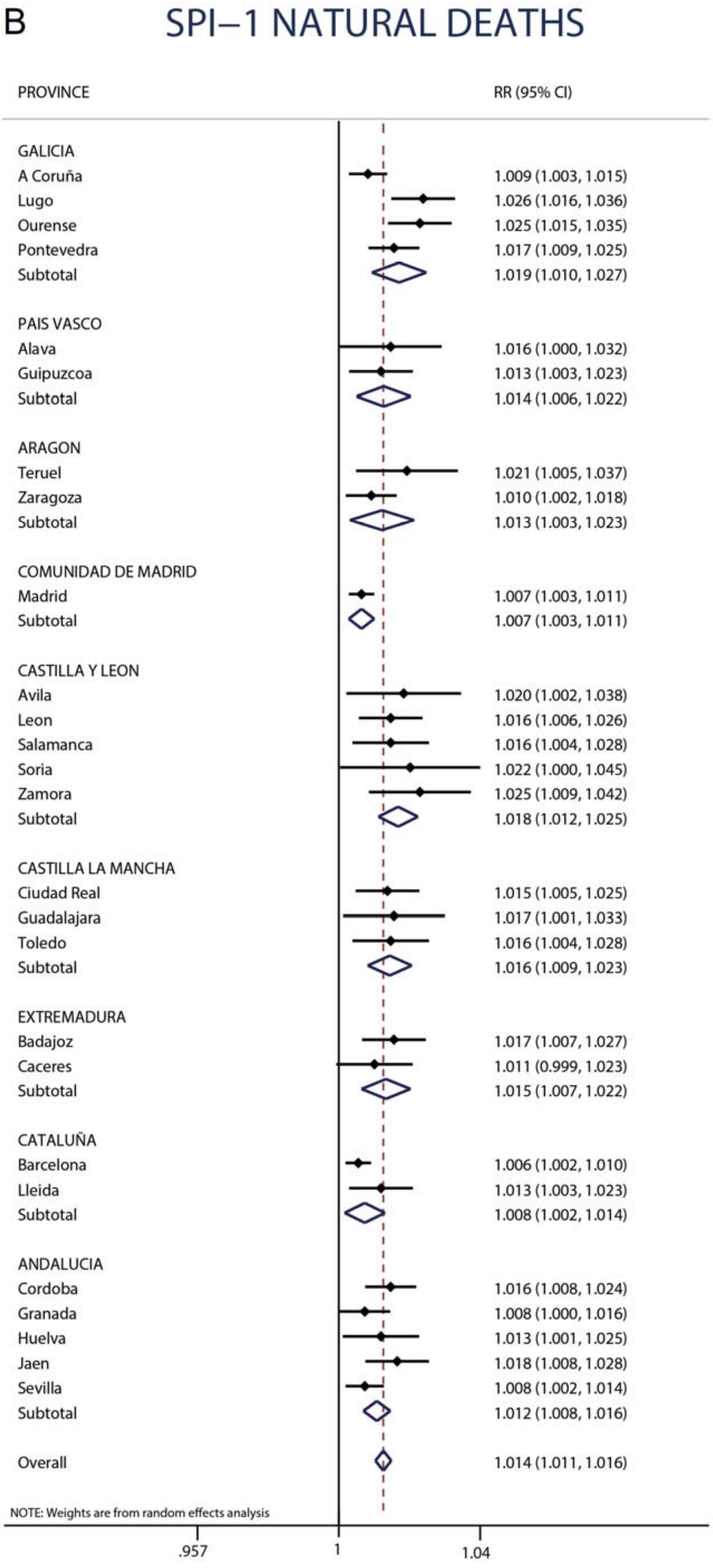
B
SPI–1 NATURAL DEATHS
PROVINCE
RR (95% CI)
GALICIA
A Coruña
1.009 (1.003, 1.015)
Lugo
1.026 (1.016, 1.036)
Ourense
1.025 (1.015, 1.035)
Pontevedra
1.017 (1.009, 1.025)
Subtotal
1.019 (1.010, 1.027)
PAIS VASCO
Alava
1.016 (1.000, 1.032)
Guipuzcoa
1.013 (1.003, 1.023)
Subtotal
1.014 (1.006, 1.022)
ARAGON
Teruel
1.021 (1.005, 1.037)
Zaragoza
1.010 (1.002, 1.018)
Subtotal
1.013 (1.003, 1.023)
COMUNIDAD DE MADRID
Madrid
1.007 (1.003, 1.011)
Subtotal
1.007 (1.003, 1.011)
CASTILLA Y LEON
Avila
1.020 (1.002, 1.038)
Leon
1.016 (1.006, 1.026)
Salamanca
1.016 (1.004, 1.028)
Soria
1.022 (1.000, 1.045)
Zamora
1.025 (1.009, 1.042)
Subtotal
1.018 (1.012, 1.025)
CASTILLA LA MANCHA
Ciudad Real
1.015 (1.005, 1.025)
Guadalajara
1.017 (1.001, 1.033)
Toledo
1.016 (1.004, 1.028)
Subtotal
1.016 (1.009, 1.023)
EXTREMADURA
Badajoz
1.017 (1.007, 1.027)
Caceres
1.011 (0.999, 1.023)
Subtotal
1.015 (1.007, 1.022)
CATALUÑA
Barcelona
1.006 (1.002, 1.010)
Lleida
1.013 (1.003, 1.023)
Subtotal
1.008 (1.002, 1.014)
ANDALUCIA
Cordoba
1.016 (1.008, 1.024)
Granada
1.008 (1.000, 1.016)
Huelva
1.013 (1.001, 1.025)
Jaen
1.018 (1.008, 1.028)
Sevilla
1.008 (1.002, 1.014)
Subtotal
1.012 (1.008, 1.016)
Overall
1.014 (1.011, 1.016)
NOTE: Weights are from random effects analysis
.957
1
1.04

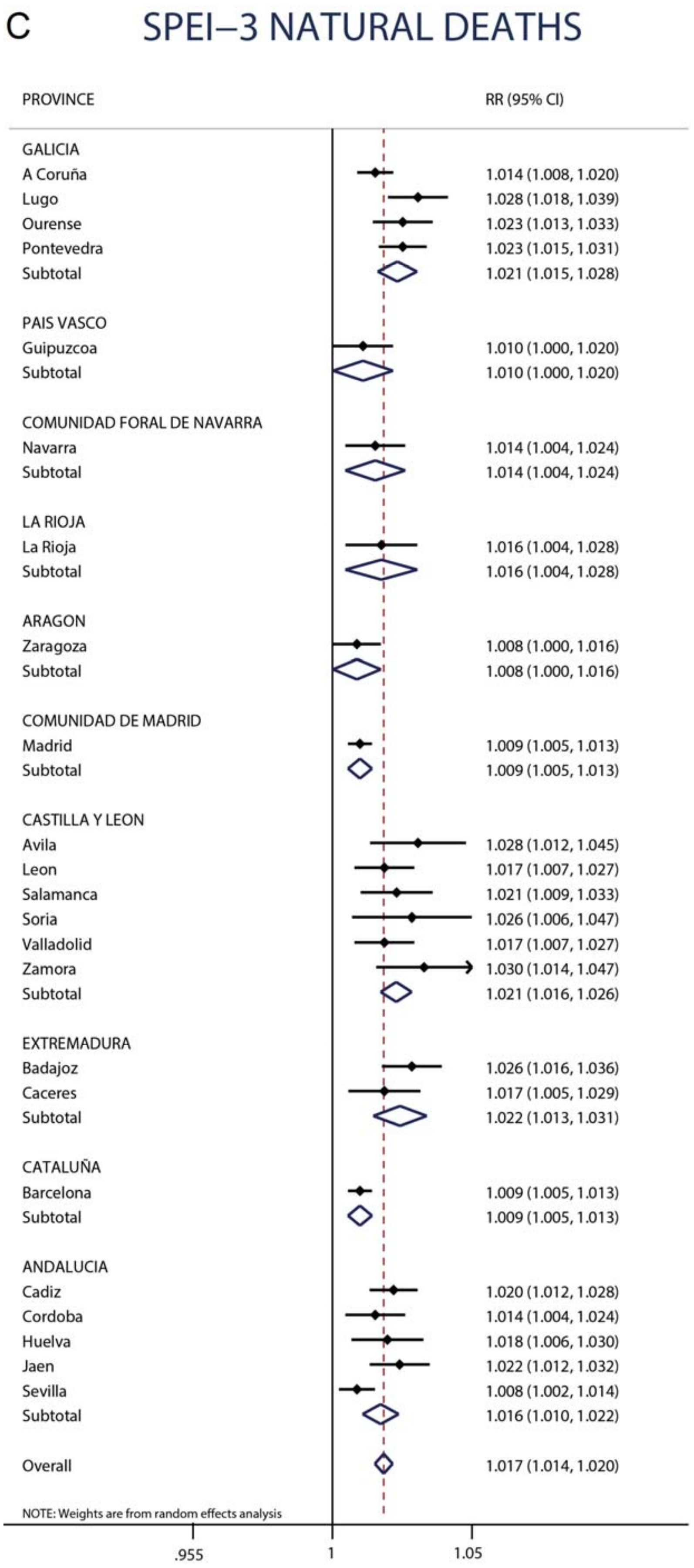
C
SPEI–3 NATURAL DEATHS
PROVINCE
RR (95% CI)
GALICIA
A Coruña 1.014 (1.008, 1.020)
Lugo 1.028 (1.018, 1.039)
Ourense 1.023 (1.013, 1.033)
Pontevedra 1.023 (1.015, 1.031)
Subtotal 1.021 (1.015, 1.028)
PAIS VASCO
Guipuzcoa 1.010 (1.000, 1.020)
Subtotal 1.010 (1.000, 1.020)
COMUNIDAD FORAL DE NAVARRA
Navarra 1.014 (1.004, 1.024)
Subtotal 1.014 (1.004, 1.024)
LA RIOJA
La Rioja 1.016 (1.004, 1.028)
Subtotal 1.016 (1.004, 1.028)
ARAGON
Zaragoza 1.008 (1.000, 1.016)
Subtotal 1.008 (1.000, 1.016)
COMUNIDAD DE MADRID
Madrid 1.009 (1.005, 1.013)
Subtotal 1.009 (1.005, 1.013)
CASTILLA Y LEON
Avila 1.028 (1.012, 1.045)
Leon 1.017 (1.007, 1.027)
Salamanca 1.021 (1.009, 1.033)
Soria 1.026 (1.006, 1.047)
Valladolid 1.017 (1.007, 1.027)
Zamora 1.030 (1.014, 1.047)
Subtotal 1.021 (1.016, 1.026)
EXTREMADURA
Badajoz 1.026 (1.016, 1.036)
Caceres 1.017 (1.005, 1.029)
Subtotal 1.022 (1.013, 1.031)
CATALUÑA
Barcelona 1.009 (1.005, 1.013)
Subtotal 1.009 (1.005, 1.013)
ANDALUCIA
Cadiz 1.020 (1.012, 1.028)
Cordoba 1.014 (1.004, 1.024)
Huelva 1.018 (1.006, 1.030)
Jaen 1.022 (1.012, 1.032)
Sevilla 1.008 (1.002, 1.014)
Subtotal 1.016 (1.010, 1.022)
Overall 1.017 (1.014, 1.020)
NOTE: Weights are from random effects analysis
.955
1
1.05

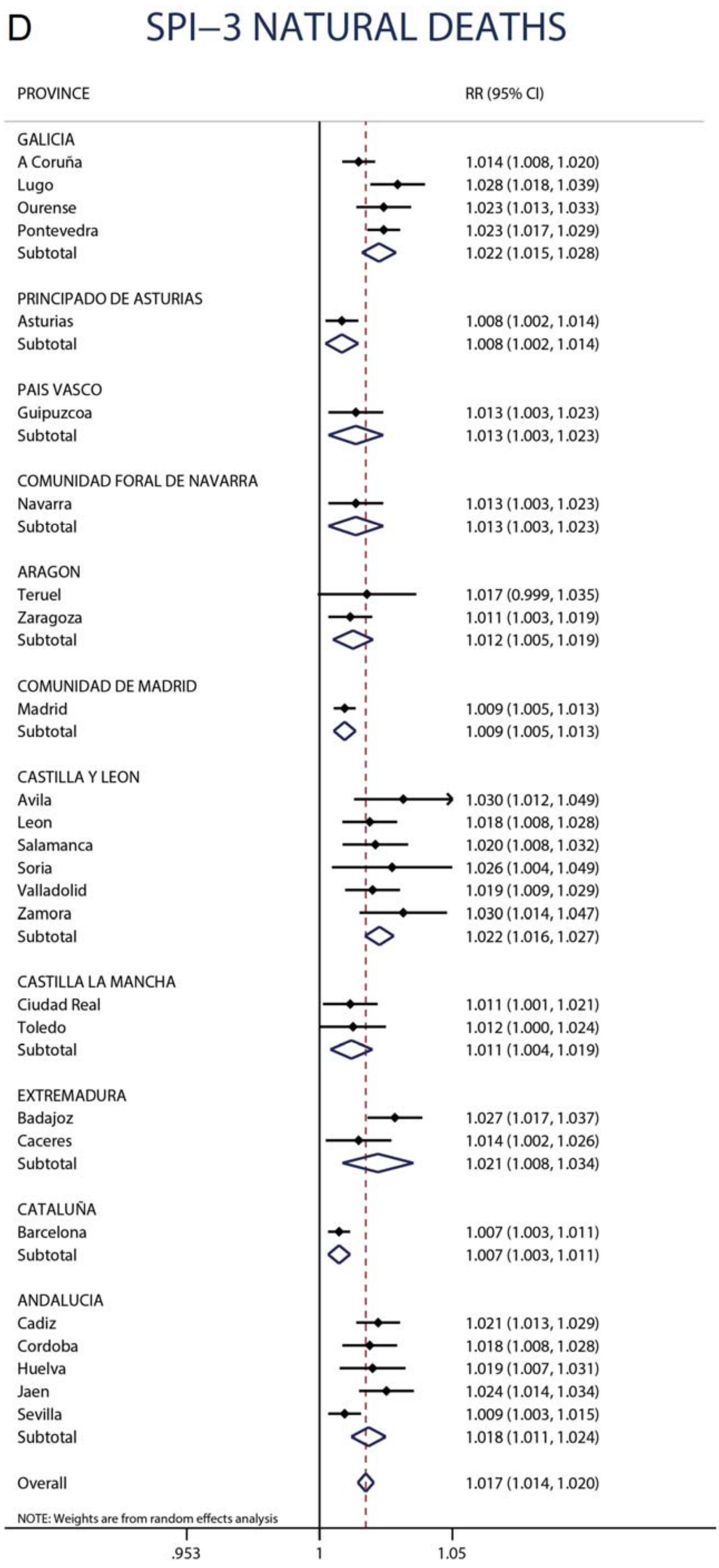
D
SPI–3 NATURAL DEATHS
PROVINCE
RR (95% CI)
GALICIA
A Coruña 1.014 (1.008, 1.020)
Lugo 1.028 (1.018, 1.039)
Ourense 1.023 (1.013, 1.033)
Pontevedra 1.023 (1.017, 1.029)
Subtotal 1.022 (1.015, 1.028)
PRINCIPADO DE ASTURIAS
Asturias 1.008 (1.002, 1.014)
Subtotal 1.008 (1.002, 1.014)
PAIS VASCO
Guipuzcoa 1.013 (1.003, 1.023)
Subtotal 1.013 (1.003, 1.023)
COMUNIDAD FORAL DE NAVARRA
Navarra 1.013 (1.003, 1.023)
Subtotal 1.013 (1.003, 1.023)
ARAGON
Teruel 1.017 (0.999, 1.035)
Zaragoza 1.011 (1.003, 1.019)
Subtotal 1.012 (1.005, 1.019)
COMUNIDAD DE MADRID
Madrid 1.009 (1.005, 1.013)
Subtotal 1.009 (1.005, 1.013)
CASTILLA Y LEON
Avila 1.030 (1.012, 1.049)
Leon 1.018 (1.008, 1.028)
Salamanca 1.020 (1.008, 1.032)
Soria 1.026 (1.004, 1.049)
Valladolid 1.019 (1.009, 1.029)
Zamora 1.030 (1.014, 1.047)
Subtotal 1.022 (1.016, 1.027)
CASTILLA LA MANCHA
Ciudad Real 1.011 (1.001, 1.021)
Toledo 1.012 (1.000, 1.024)
Subtotal 1.011 (1.004, 1.019)
EXTREMADURA
Badajoz 1.027 (1.017, 1.037)
Caceres 1.014 (1.002, 1.026)
Subtotal 1.021 (1.008, 1.034)
CATALUÑA
Barcelona 1.007 (1.003, 1.011)
Subtotal 1.007 (1.003, 1.011)
ANDALUCIA
Cadiz 1.021 (1.013, 1.029)
Cordoba 1.018 (1.008, 1.028)
Huelva 1.019 (1.007, 1.031)
Jaen 1.024 (1.014, 1.034)
Sevilla 1.009 (1.003, 1.015)
Subtotal 1.018 (1.011, 1.024)
Overall 1.017 (1.014, 1.020)
NOTE: Weights are from random effects analysis
.953
1
1.05

Figure S1. Forest plots of the relative risk (RR) values of daily natural mortality associated with droughts by the administrative subdivisions of peninsular Spain, i.e., the Autonomous Communities and their provinces. **A** and **B**: Droughts measured by the Standardized Precipitation Evapotranspiration Index (SPEI) and the Standardized Precipitation Index (SPI) obtained at one month of drought accumulation (SPEI-1 and SPI-1, respectively). **C** and **D**: As per A and B, but for three months of accumulation (SPEI-3 and SPI-3, respectively). Only provinces with a statistically significant association ($p<0.05$) between drought indices and natural deaths are shown. Provincial RR data obtained with the use of both SPEI-1 and SPI-1 from Salvador et al., 2020.

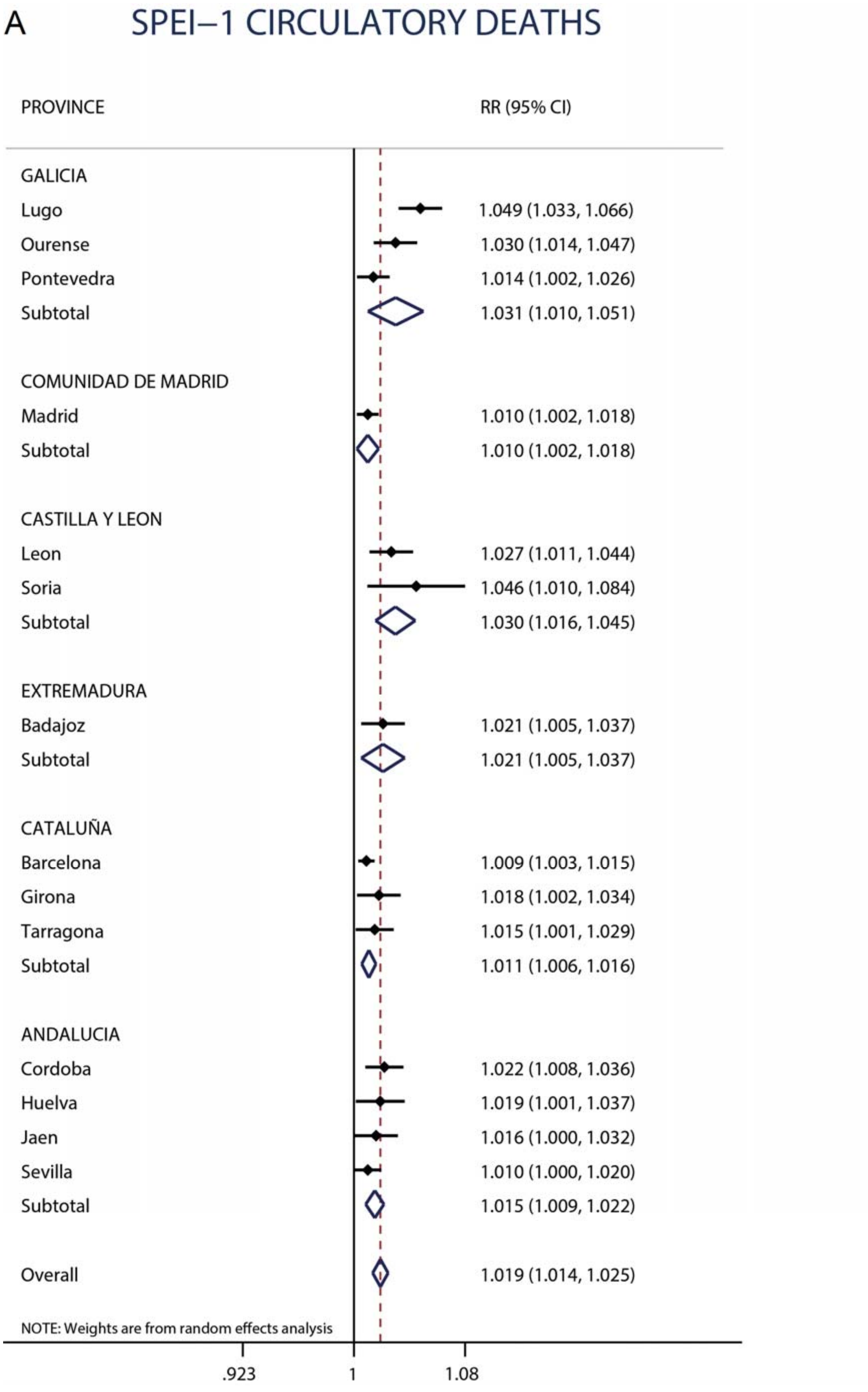
A
SPEI–1 CIRCULATORY DEATHS
PROVINCE
RR (95% CI)
GALICIA
Lugo
1.049 (1.033, 1.066)
Ourense
1.030 (1.014, 1.047)
Pontevedra
1.014 (1.002, 1.026)
Subtotal
1.031 (1.010, 1.051)
COMUNIDAD DE MADRID
Madrid
1.010 (1.002, 1.018)
Subtotal
1.010 (1.002, 1.018)
CASTILLA Y LEON
Leon
1.027 (1.011, 1.044)
Soria
1.046 (1.010, 1.084)
Subtotal
1.030 (1.016, 1.045)
EXTREMADURA
Badajoz
1.021 (1.005, 1.037)
Subtotal
1.021 (1.005, 1.037)
CATALUÑA
Barcelona
1.009 (1.003, 1.015)
Girona
1.018 (1.002, 1.034)
Tarragona
1.015 (1.001, 1.029)
Subtotal
1.011 (1.006, 1.016)
ANDALUCIA
Cordoba
1.022 (1.008, 1.036)
Huelva
1.019 (1.001, 1.037)
Jaen
1.016 (1.000, 1.032)
Sevilla
1.010 (1.000, 1.020)
Subtotal
1.015 (1.009, 1.022)
Overall
1.019 (1.014, 1.025)
NOTE: Weights are from random effects analysis
.923
1
1.08

B

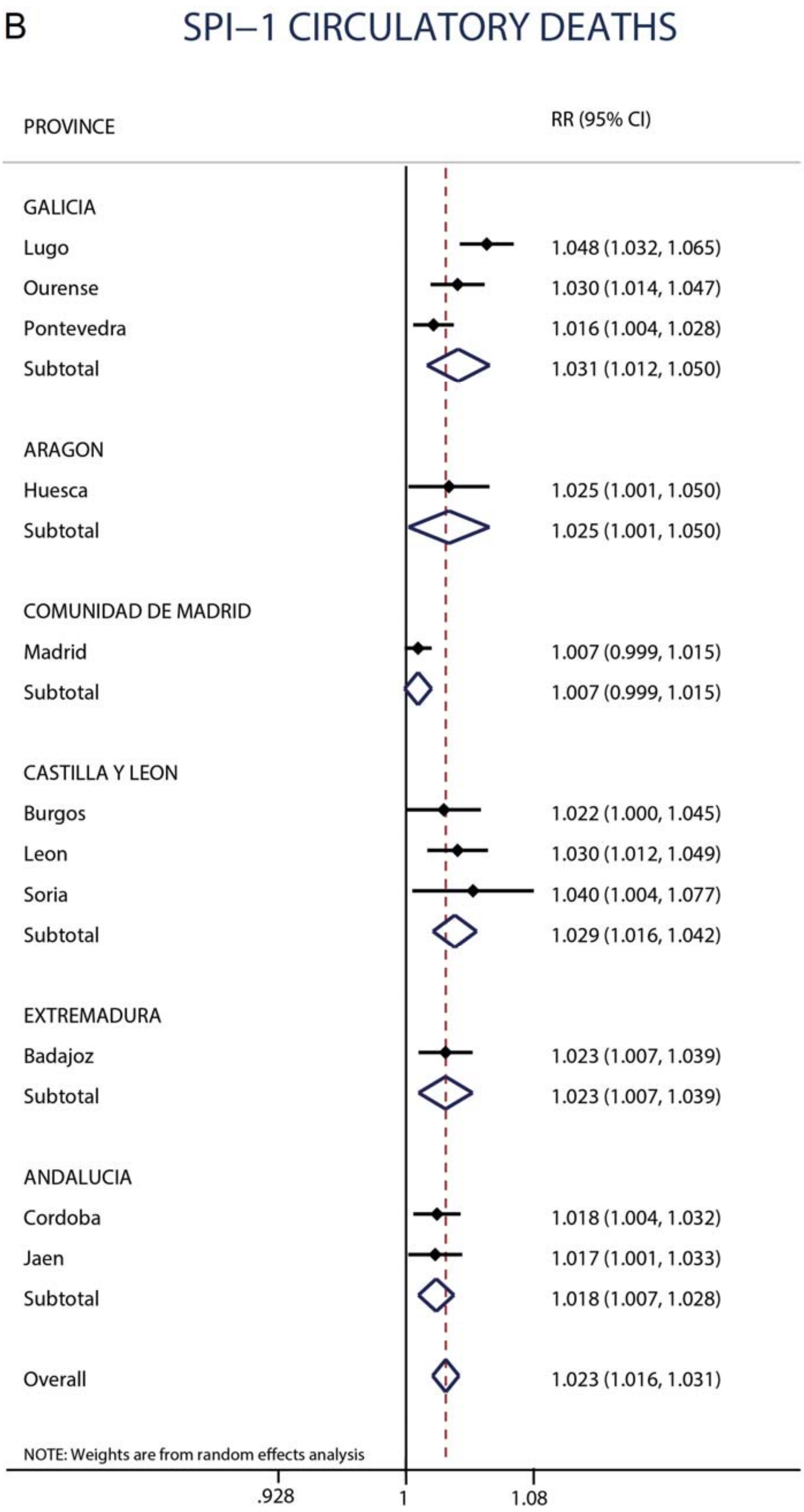
SPI−1 CIRCULATORY DEATHS
PROVINCE
RR (95% CI)
GALICIA
Lugo
1.048 (1.032, 1.065)
Ourense
1.030 (1.014, 1.047)
Pontevedra
1.016 (1.004, 1.028)
Subtotal
1.031 (1.012, 1.050)
ARAGON
Huesca
1.025 (1.001, 1.050)
Subtotal
1.025 (1.001, 1.050)
COMUNIDAD DE MADRID
Madrid
1.007 (0.999, 1.015)
Subtotal
1.007 (0.999, 1.015)
CASTILLA Y LEON
Burgos
1.022 (1.000, 1.045)
Leon
1.030 (1.012, 1.049)
Soria
1.040 (1.004, 1.077)
Subtotal
1.029 (1.016, 1.042)
EXTREMADURA
Badajoz
1.023 (1.007, 1.039)
Subtotal
1.023 (1.007, 1.039)
ANDALUCIA
Cordoba
1.018 (1.004, 1.032)
Jaen
1.017 (1.001, 1.033)
Subtotal
1.018 (1.007, 1.028)
Overall
1.023 (1.016, 1.031)
NOTE: Weights are from random effects analysis
.928
1
1.08

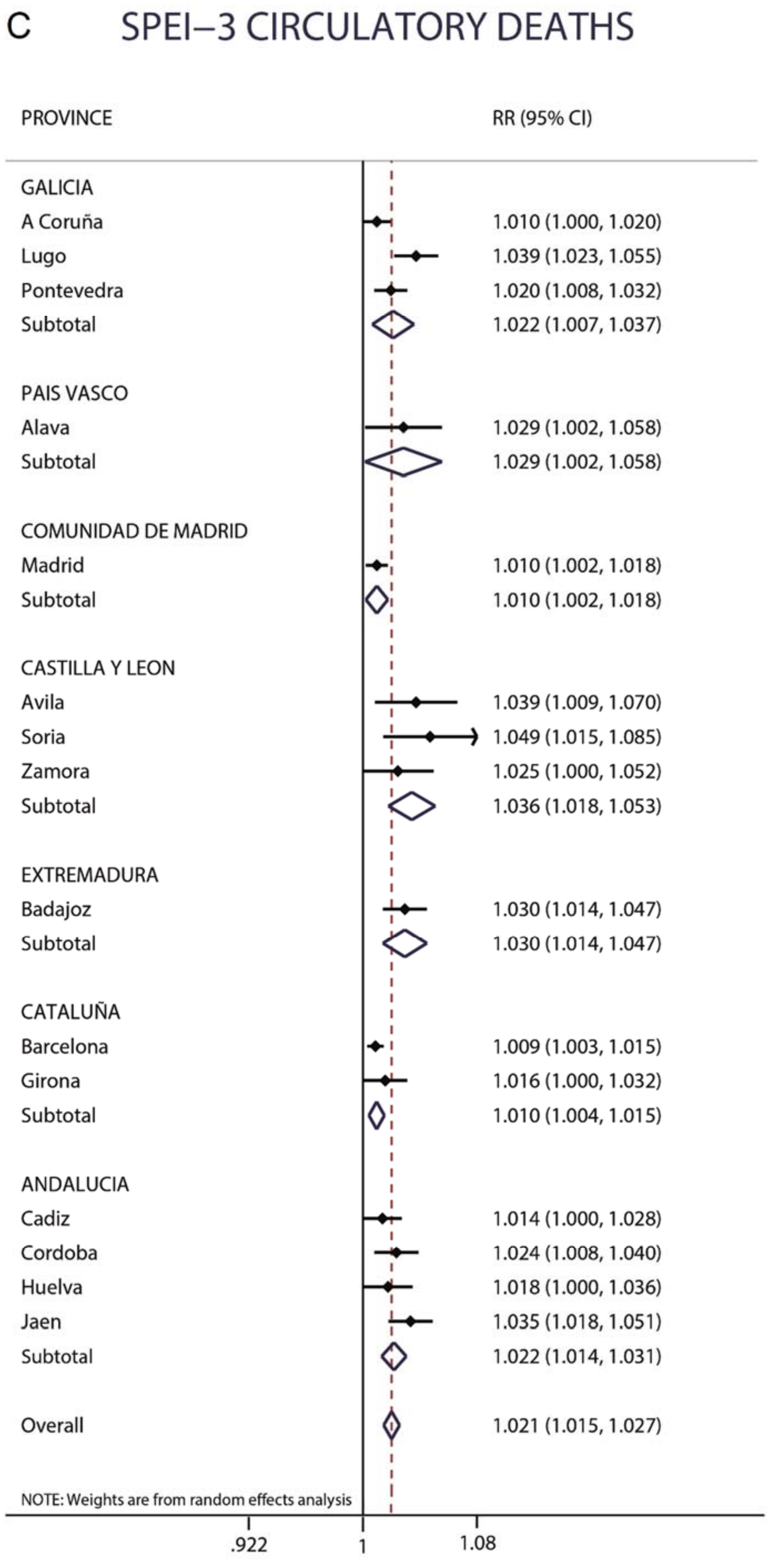
C
SPEI–3 CIRCULATORY DEATHS
PROVINCE
RR (95% CI)
GALICIA
A Coruña
1.010 (1.000, 1.020)
Lugo
1.039 (1.023, 1.055)
Pontevedra
1.020 (1.008, 1.032)
Subtotal
1.022 (1.007, 1.037)
PAIS VASCO
Alava
1.029 (1.002, 1.058)
Subtotal
1.029 (1.002, 1.058)
COMUNIDAD DE MADRID
Madrid
1.010 (1.002, 1.018)
Subtotal
1.010 (1.002, 1.018)
CASTILLA Y LEON
Avila
1.039 (1.009, 1.070)
Soria
1.049 (1.015, 1.085)
Zamora
1.025 (1.000, 1.052)
Subtotal
1.036 (1.018, 1.053)
EXTREMADURA
Badajoz
1.030 (1.014, 1.047)
Subtotal
1.030 (1.014, 1.047)
CATALUÑA
Barcelona
1.009 (1.003, 1.015)
Girona
1.016 (1.000, 1.032)
Subtotal
1.010 (1.004, 1.015)
ANDALUCIA
Cadiz
1.014 (1.000, 1.028)
Cordoba
1.024 (1.008, 1.040)
Huelva
1.018 (1.000, 1.036)
Jaen
1.035 (1.018, 1.051)
Subtotal
1.022 (1.014, 1.031)
Overall
1.021 (1.015, 1.027)
NOTE: Weights are from random effects analysis
.922
1
1.08

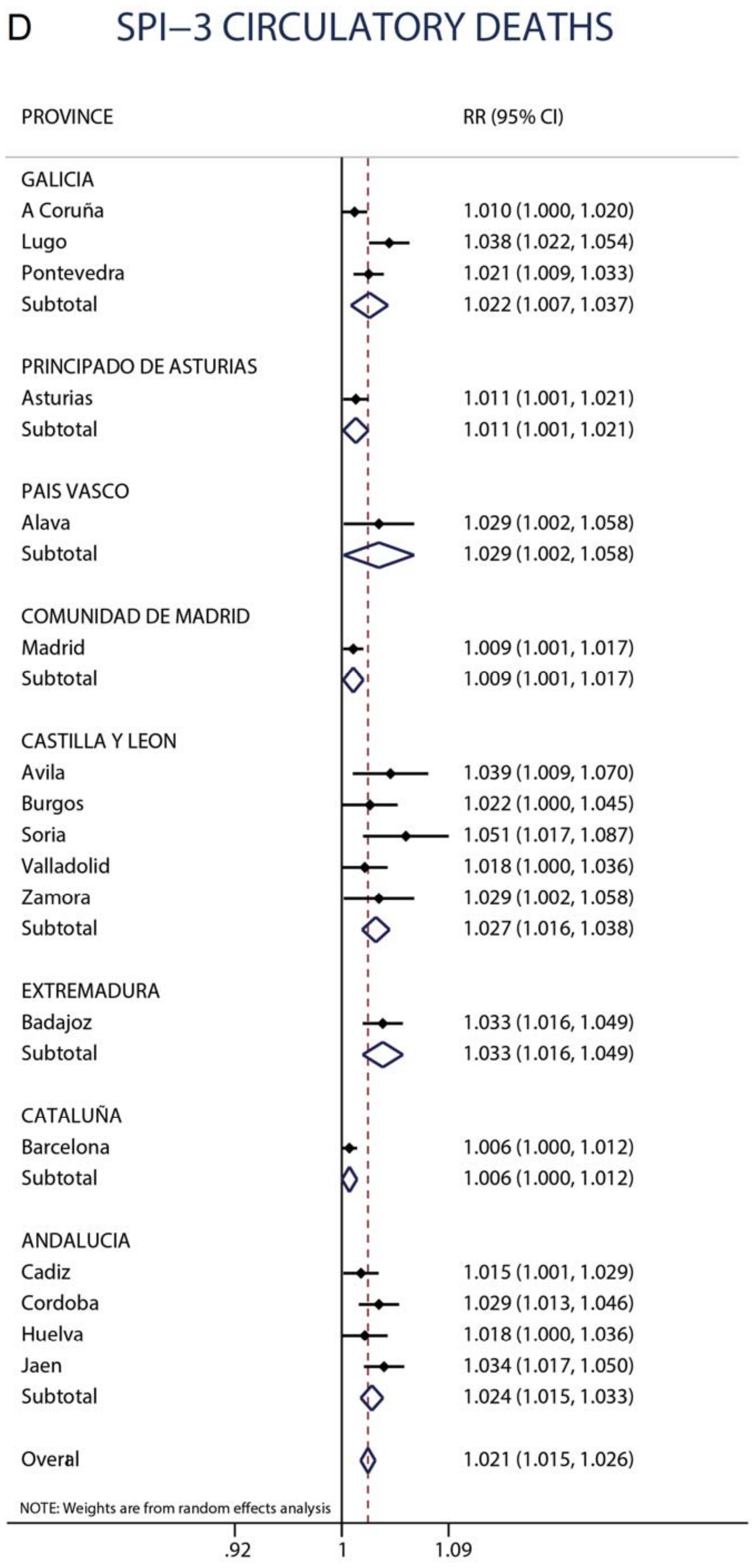
D
SPI–3 CIRCULATORY DEATHS
PROVINCE
RR (95% CI)
GALICIA
A Coruña 1.010 (1.000, 1.020)
Lugo 1.038 (1.022, 1.054)
Pontevedra 1.021 (1.009, 1.033)
Subtotal 1.022 (1.007, 1.037)
PRINCIPADO DE ASTURIAS
Asturias 1.011 (1.001, 1.021)
Subtotal 1.011 (1.001, 1.021)
PAIS VASCO
Alava 1.029 (1.002, 1.058)
Subtotal 1.029 (1.002, 1.058)
COMUNIDAD DE MADRID
Madrid 1.009 (1.001, 1.017)
Subtotal 1.009 (1.001, 1.017)
CASTILLA Y LEON
Avila 1.039 (1.009, 1.070)
Burgos 1.022 (1.000, 1.045)
Soria 1.051 (1.017, 1.087)
Valladolid 1.018 (1.000, 1.036)
Zamora 1.029 (1.002, 1.058)
Subtotal 1.027 (1.016, 1.038)
EXTREMADURA
Badajoz 1.033 (1.016, 1.049)
Subtotal 1.033 (1.016, 1.049)
CATALUÑA
Barcelona 1.006 (1.000, 1.012)
Subtotal 1.006 (1.000, 1.012)
ANDALUCIA
Cadiz 1.015 (1.001, 1.029)
Cordoba 1.029 (1.013, 1.046)
Huelva 1.018 (1.000, 1.036)
Jaen 1.034 (1.017, 1.050)
Subtotal 1.024 (1.015, 1.033)
Overal 1.021 (1.015, 1.026)
NOTE: Weights are from random effects analysis
.92
1
1.09

Figure 2. Forest plots of the relative risk (RR) values of daily circulatory mortality associated with droughts by the administrative subdivisions of peninsular Spain, i.e., the Autonomous Communities and their provinces. **A** and **B**: Droughts measured by the Standardized Precipitation Evapotranspiration Index (SPEI) and the Standardized Precipitation Index (SPI) obtained at one month of drought accumulation (SPEI-1 and SPI-1, respectively). **C** and **D**: As per A and B, but for three months of accumulation (SPEI-3 and SPI-3, respectively). Only provinces with a statistically significant association ($p<0.05$) between drought indices and circulatory deaths are shown. Provincial RR data obtained with the use of both SPEI-1 and SPI-1 from Salvador et al., 2020.

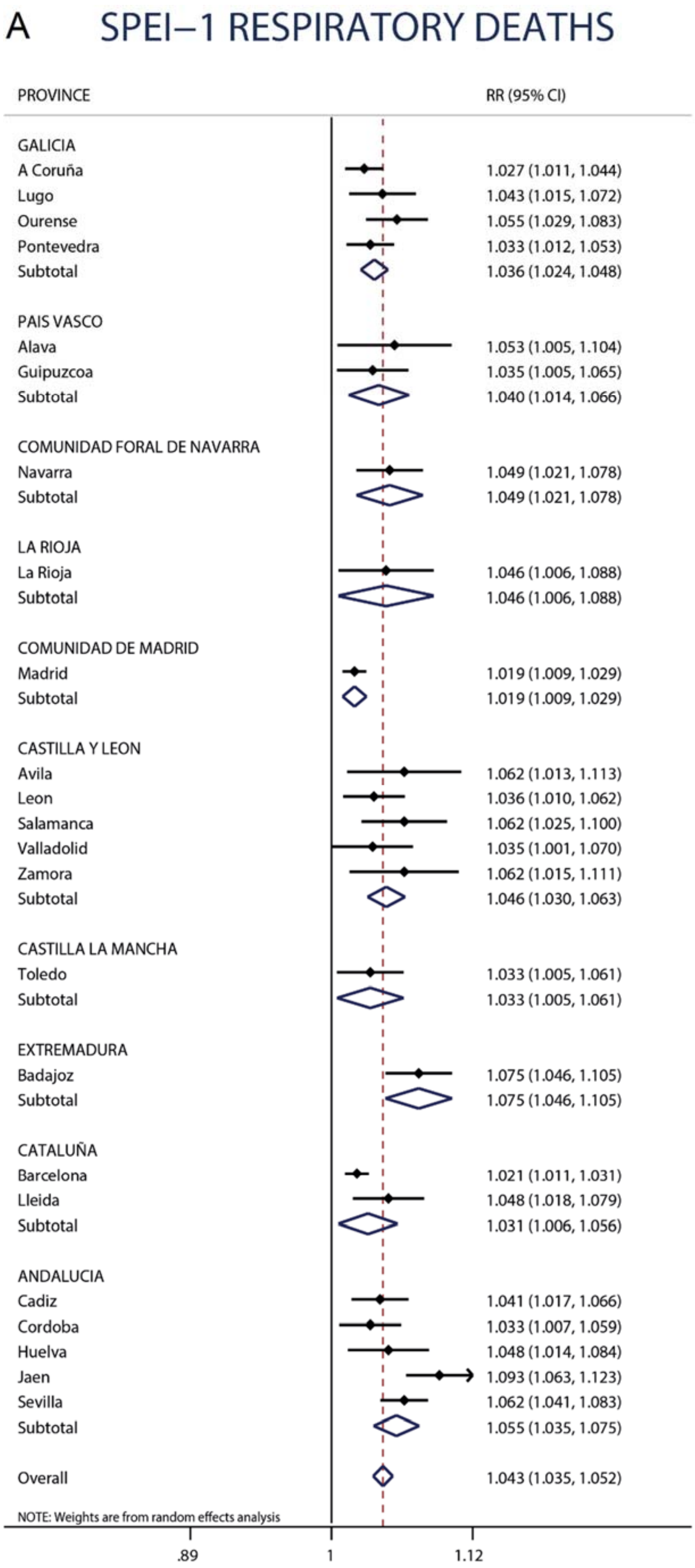
A
SPEI–1 RESPIRATORY DEATHS
PROVINCE
RR (95% CI)
GALICIA
A Coruña
1.027 (1.011, 1.044)
Lugo
1.043 (1.015, 1.072)
Ourense
1.055 (1.029, 1.083)
Pontevedra
1.033 (1.012, 1.053)
Subtotal
1.036 (1.024, 1.048)
PAIS VASCO
Alava
1.053 (1.005, 1.104)
Guipuzcoa
1.035 (1.005, 1.065)
Subtotal
1.040 (1.014, 1.066)
COMUNIDAD FORAL DE NAVARRA
Navarra
1.049 (1.021, 1.078)
Subtotal
1.049 (1.021, 1.078)
LA RIOJA
La Rioja
1.046 (1.006, 1.088)
Subtotal
1.046 (1.006, 1.088)
COMUNIDAD DE MADRID
Madrid
1.019 (1.009, 1.029)
Subtotal
1.019 (1.009, 1.029)
CASTILLA Y LEON
Avila
1.062 (1.013, 1.113)
Leon
1.036 (1.010, 1.062)
Salamanca
1.062 (1.025, 1.100)
Valladolid
1.035 (1.001, 1.070)
Zamora
1.062 (1.015, 1.111)
Subtotal
1.046 (1.030, 1.063)
CASTILLA LA MANCHA
Toledo
1.033 (1.005, 1.061)
Subtotal
1.033 (1.005, 1.061)
EXTREMADURA
Badajoz
1.075 (1.046, 1.105)
Subtotal
1.075 (1.046, 1.105)
CATALUÑA
Barcelona
1.021 (1.011, 1.031)
Lleida
1.048 (1.018, 1.079)
Subtotal
1.031 (1.006, 1.056)
ANDALUCIA
Cadiz
1.041 (1.017, 1.066)
Cordoba
1.033 (1.007, 1.059)
Huelva
1.048 (1.014, 1.084)
Jaen
1.093 (1.063, 1.123)
Sevilla
1.062 (1.041, 1.083)
Subtotal
1.055 (1.035, 1.075)
Overall
1.043 (1.035, 1.052)
NOTE: Weights are from random effects analysis
.89
1
1.12

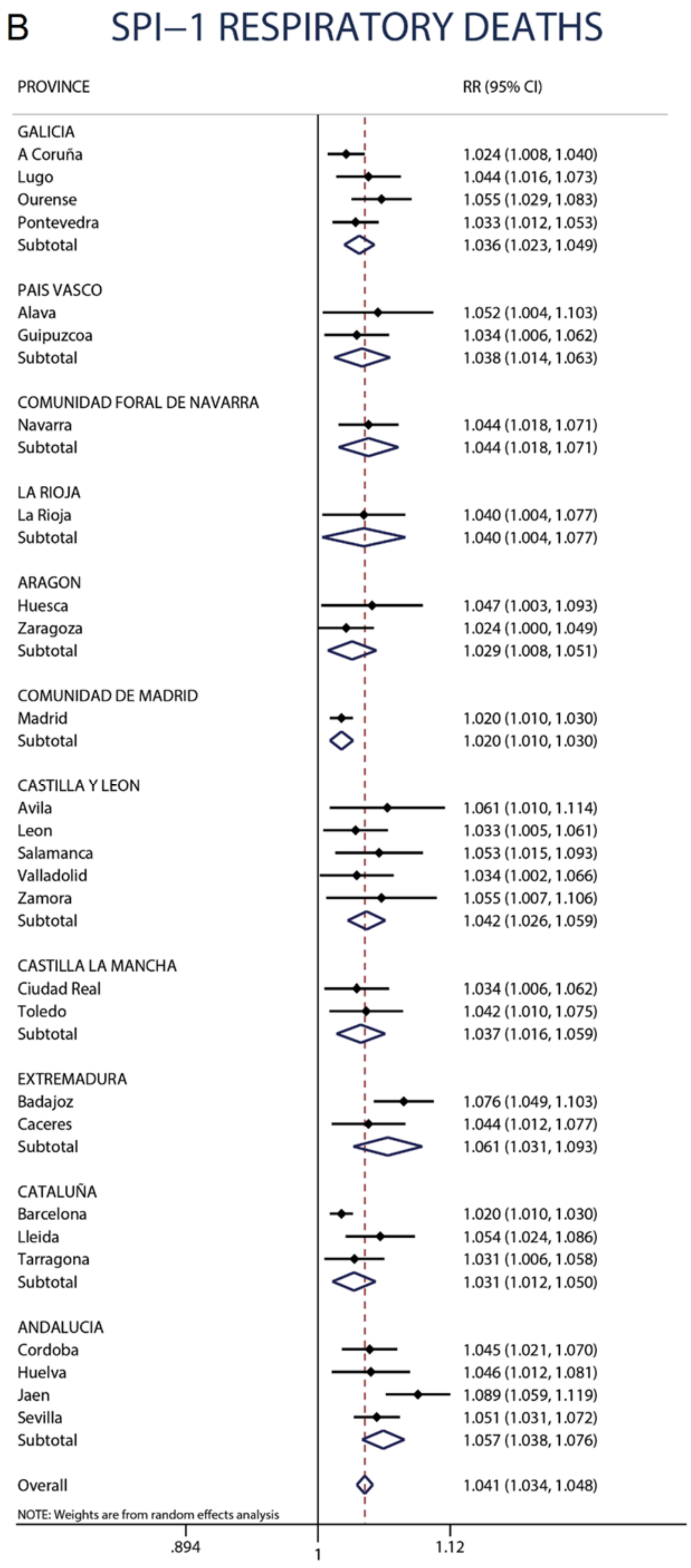
B
SPI–1 RESPIRATORY DEATHS
PROVINCE
RR (95% CI)
GALICIA
A Coruña 1.024 (1.008, 1.040)
Lugo 1.044 (1.016, 1.073)
Ourense 1.055 (1.029, 1.083)
Pontevedra 1.033 (1.012, 1.053)
Subtotal 1.036 (1.023, 1.049)
PAIS VASCO
Alava 1.052 (1.004, 1.103)
Guipuzcoa 1.034 (1.006, 1.062)
Subtotal 1.038 (1.014, 1.063)
COMUNIDAD FORAL DE NAVARRA
Navarra 1.044 (1.018, 1.071)
Subtotal 1.044 (1.018, 1.071)
LA RIOJA
La Rioja 1.040 (1.004, 1.077)
Subtotal 1.040 (1.004, 1.077)
ARAGON
Huesca 1.047 (1.003, 1.093)
Zaragoza 1.024 (1.000, 1.049)
Subtotal 1.029 (1.008, 1.051)
COMUNIDAD DE MADRID
Madrid 1.020 (1.010, 1.030)
Subtotal 1.020 (1.010, 1.030)
CASTILLA Y LEON
Avila 1.061 (1.010, 1.114)
Leon 1.033 (1.005, 1.061)
Salamanca 1.053 (1.015, 1.093)
Valladolid 1.034 (1.002, 1.066)
Zamora 1.055 (1.007, 1.106)
Subtotal 1.042 (1.026, 1.059)
CASTILLA LA MANCHA
Ciudad Real 1.034 (1.006, 1.062)
Toledo 1.042 (1.010, 1.075)
Subtotal 1.037 (1.016, 1.059)
EXTREMADURA
Badajoz 1.076 (1.049, 1.103)
Caceres 1.044 (1.012, 1.077)
Subtotal 1.061 (1.031, 1.093)
CATALUÑA
Barcelona 1.020 (1.010, 1.030)
Lleida 1.054 (1.024, 1.086)
Tarragona 1.031 (1.006, 1.058)
Subtotal 1.031 (1.012, 1.050)
ANDALUCIA
Cordoba 1.045 (1.021, 1.070)
Huelva 1.046 (1.012, 1.081)
Jaen 1.089 (1.059, 1.119)
Sevilla 1.051 (1.031, 1.072)
Subtotal 1.057 (1.038, 1.076)
Overall 1.041 (1.034, 1.048)
NOTE: Weights are from random effects analysis
.894
1
1.12

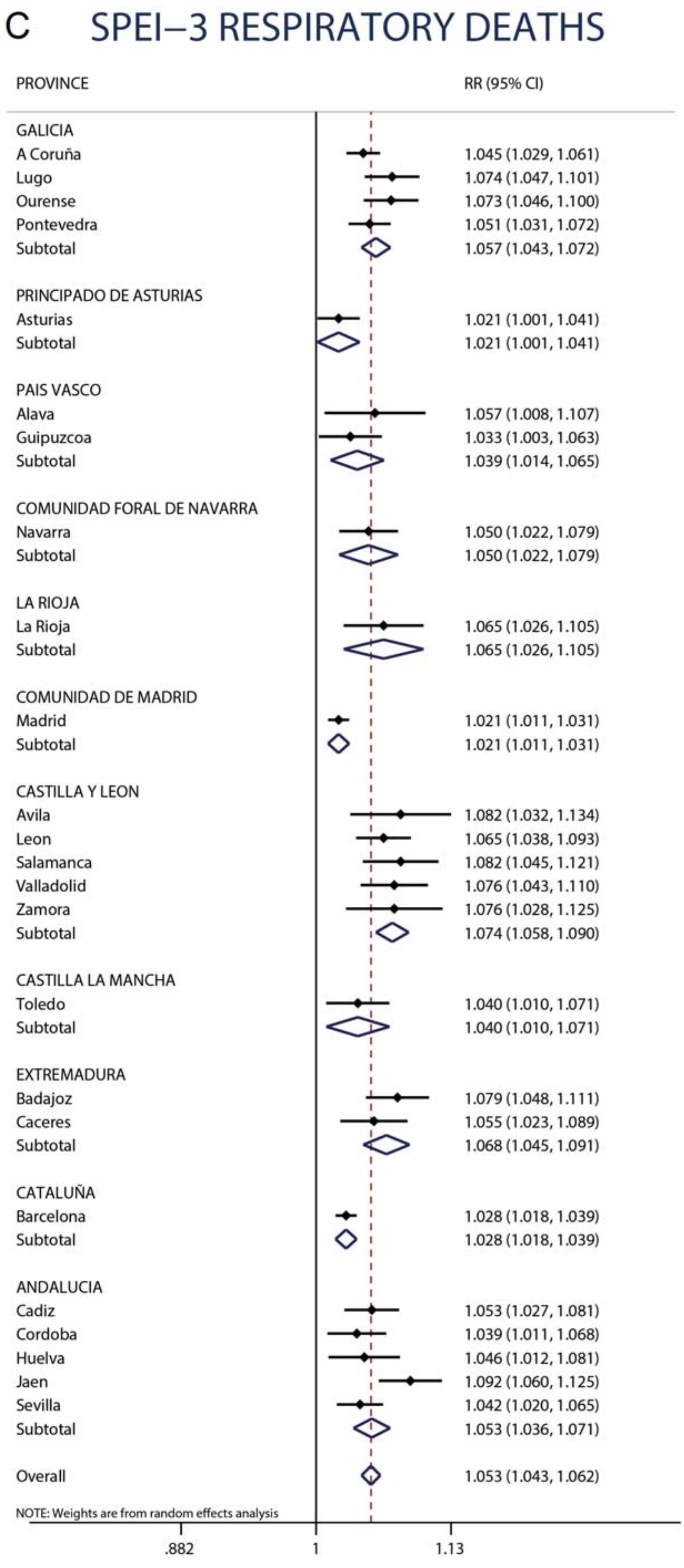
C
SPEI–3 RESPIRATORY DEATHS
PROVINCE
RR (95% CI)
GALICIA
A Coruña 1.045 (1.029, 1.061)
Lugo 1.074 (1.047, 1.101)
Ourense 1.073 (1.046, 1.100)
Pontevedra 1.051 (1.031, 1.072)
Subtotal 1.057 (1.043, 1.072)
PRINCIPADO DE ASTURIAS
Asturias 1.021 (1.001, 1.041)
Subtotal 1.021 (1.001, 1.041)
PAIS VASCO
Alava 1.057 (1.008, 1.107)
Guipuzcoa 1.033 (1.003, 1.063)
Subtotal 1.039 (1.014, 1.065)
COMUNIDAD FORAL DE NAVARRA
Navarra 1.050 (1.022, 1.079)
Subtotal 1.050 (1.022, 1.079)
LA RIOJA
La Rioja 1.065 (1.026, 1.105)
Subtotal 1.065 (1.026, 1.105)
COMUNIDAD DE MADRID
Madrid 1.021 (1.011, 1.031)
Subtotal 1.021 (1.011, 1.031)
CASTILLA Y LEON
Avila 1.082 (1.032, 1.134)
Leon 1.065 (1.038, 1.093)
Salamanca 1.082 (1.045, 1.121)
Valladolid 1.076 (1.043, 1.110)
Zamora 1.076 (1.028, 1.125)
Subtotal 1.074 (1.058, 1.090)
CASTILLA LA MANCHA
Toledo 1.040 (1.010, 1.071)
Subtotal 1.040 (1.010, 1.071)
EXTREMADURA
Badajoz 1.079 (1.048, 1.111)
Caceres 1.055 (1.023, 1.089)
Subtotal 1.068 (1.045, 1.091)
CATALUÑA
Barcelona 1.028 (1.018, 1.039)
Subtotal 1.028 (1.018, 1.039)
ANDALUCIA
Cadiz 1.053 (1.027, 1.081)
Cordoba 1.039 (1.011, 1.068)
Huelva 1.046 (1.012, 1.081)
Jaen 1.092 (1.060, 1.125)
Sevilla 1.042 (1.020, 1.065)
Subtotal 1.053 (1.036, 1.071)
Overall 1.053 (1.043, 1.062)
NOTE: Weights are from random effects analysis
.882
1
1.13

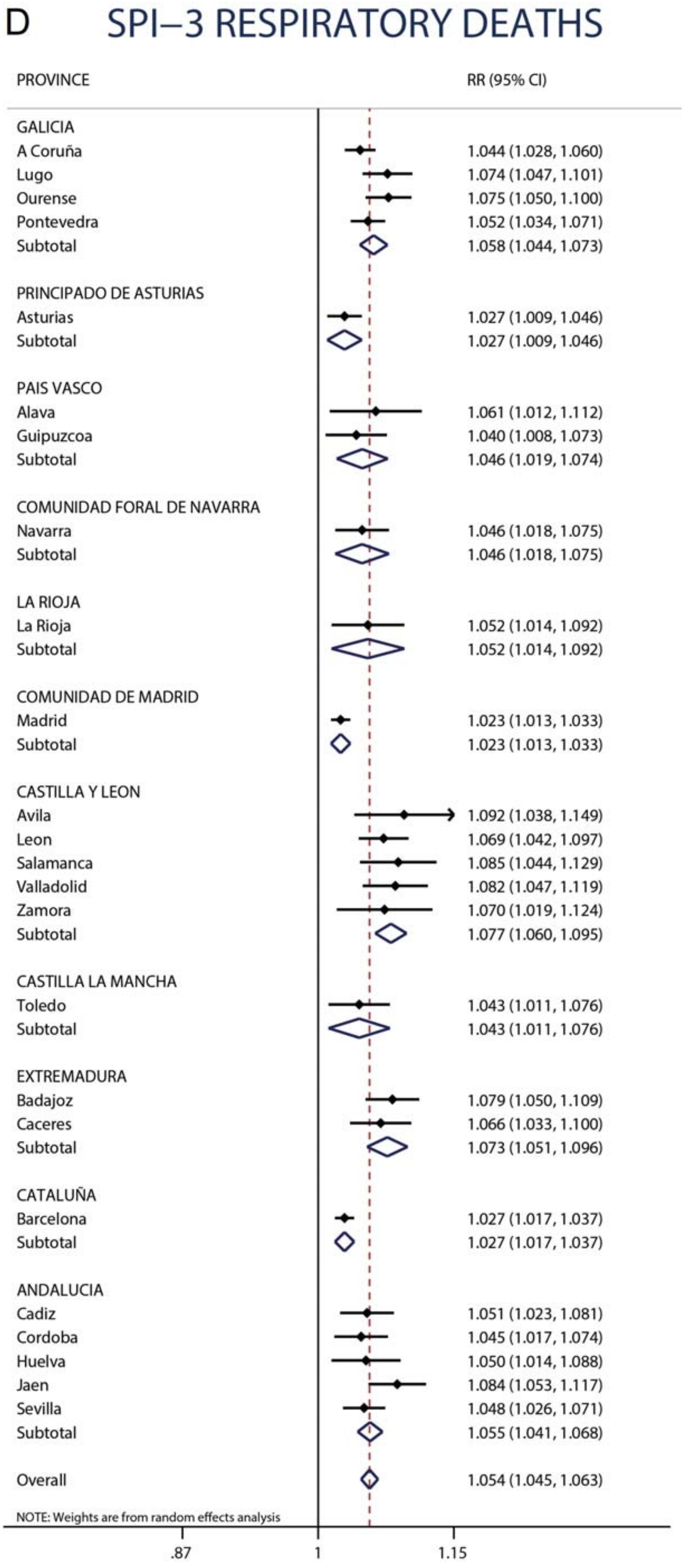
D
SPI–3 RESPIRATORY DEATHS
PROVINCE
RR (95% CI)
GALICIA
A Coruña 1.044 (1.028, 1.060)
Lugo 1.074 (1.047, 1.101)
Ourense 1.075 (1.050, 1.100)
Pontevedra 1.052 (1.034, 1.071)
Subtotal 1.058 (1.044, 1.073)
PRINCIPADO DE ASTURIAS
Asturias 1.027 (1.009, 1.046)
Subtotal 1.027 (1.009, 1.046)
PAIS VASCO
Alava 1.061 (1.012, 1.112)
Guipuzcoa 1.040 (1.008, 1.073)
Subtotal 1.046 (1.019, 1.074)
COMUNIDAD FORAL DE NAVARRA
Navarra 1.046 (1.018, 1.075)
Subtotal 1.046 (1.018, 1.075)
LA RIOJA
La Rioja 1.052 (1.014, 1.092)
Subtotal 1.052 (1.014, 1.092)
COMUNIDAD DE MADRID
Madrid 1.023 (1.013, 1.033)
Subtotal 1.023 (1.013, 1.033)
CASTILLA Y LEON
Avila 1.092 (1.038, 1.149)
Leon 1.069 (1.042, 1.097)
Salamanca 1.085 (1.044, 1.129)
Valladolid 1.082 (1.047, 1.119)
Zamora 1.070 (1.019, 1.124)
Subtotal 1.077 (1.060, 1.095)
CASTILLA LA MANCHA
Toledo 1.043 (1.011, 1.076)
Subtotal 1.043 (1.011, 1.076)
EXTREMADURA
Badajoz 1.079 (1.050, 1.109)
Caceres 1.066 (1.033, 1.100)
Subtotal 1.073 (1.051, 1.096)
CATALUÑA
Barcelona 1.027 (1.017, 1.037)
Subtotal 1.027 (1.017, 1.037)
ANDALUCIA
Cadiz 1.051 (1.023, 1.081)
Cordoba 1.045 (1.017, 1.074)
Huelva 1.050 (1.014, 1.088)
Jaen 1.084 (1.053, 1.117)
Sevilla 1.048 (1.026, 1.071)
Subtotal 1.055 (1.041, 1.068)
Overall 1.054 (1.045, 1.063)
NOTE: Weights are from random effects analysis
.87
1
1.15

Figure S3. Forest plots of the relative risk (RR) values of daily respiratory mortality associated with droughts by the administrative subdivisions of peninsular Spain, i.e., the Autonomous Communities and their provinces. **A** and **B**: Droughts measured by the Standardized Precipitation Evapotranspiration Index (SPEI) and the Standardized Precipitation Index (SPI) obtained at one month of drought accumulation (SPEI-1 and SPI-1, respectively). **C** and **D**: As per A and B, but for three months of accumulation (SPEI-3 and SPI-3, respectively). Only provinces with a statistically significant association ($p<0.05$) between drought indices and respiratory deaths are shown. Provincial RR data obtained with the use of both SPEI-1 and SPI-1 from Salvador et al., 2020.

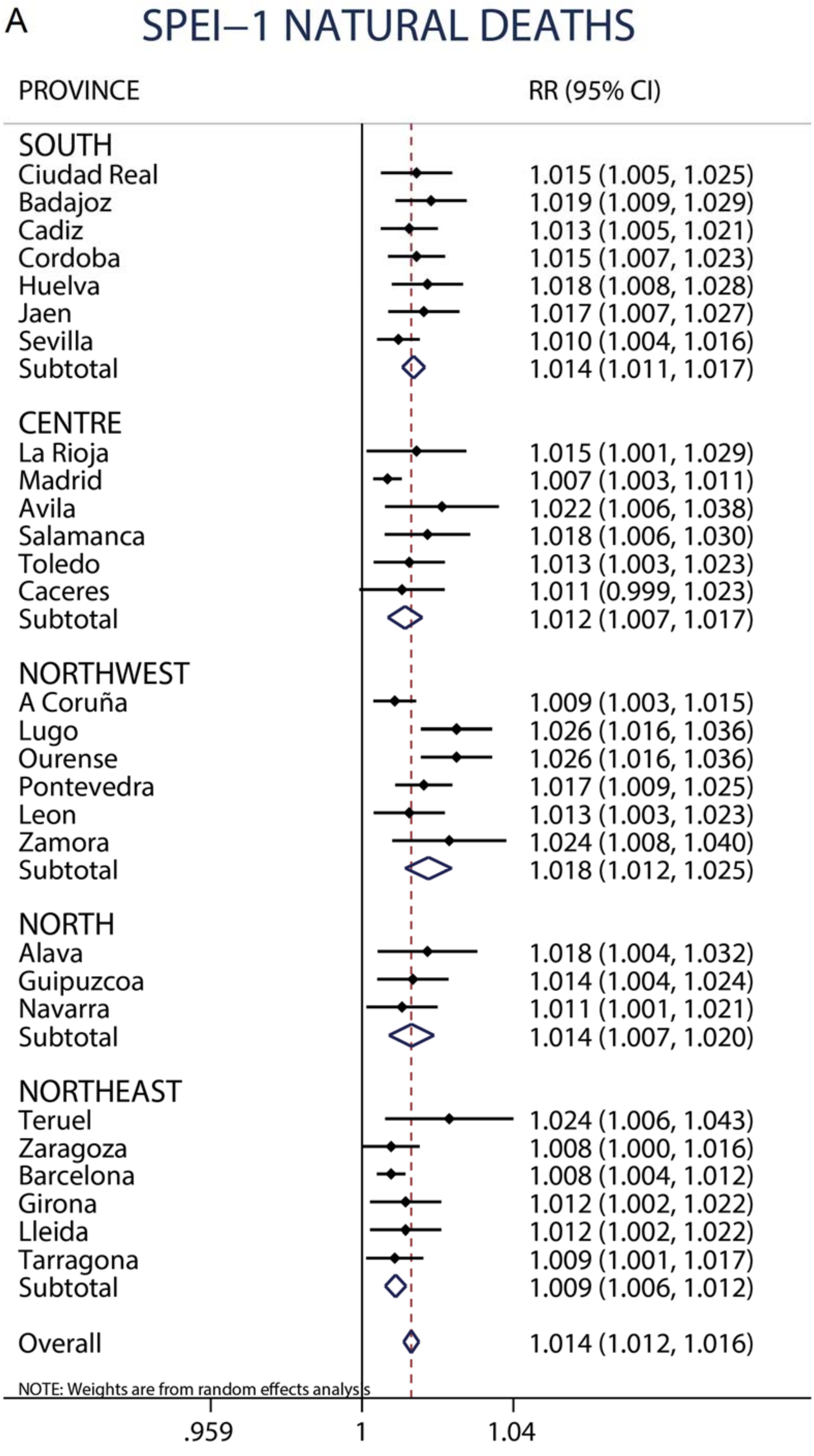
A
SPEI−1 NATURAL DEATHS
PROVINCE
RR (95% CI)
SOUTH
Ciudad Real 1.015 (1.005, 1.025)
Badajoz 1.019 (1.009, 1.029)
Cadiz 1.013 (1.005, 1.021)
Cordoba 1.015 (1.007, 1.023)
Huelva 1.018 (1.008, 1.028)
Jaen 1.017 (1.007, 1.027)
Sevilla 1.010 (1.004, 1.016)
Subtotal 1.014 (1.011, 1.017)
CENTRE
La Rioja 1.015 (1.001, 1.029)
Madrid 1.007 (1.003, 1.011)
Avila 1.022 (1.006, 1.038)
Salamanca 1.018 (1.006, 1.030)
Toledo 1.013 (1.003, 1.023)
Caceres 1.011 (0.999, 1.023)
Subtotal 1.012 (1.007, 1.017)
NORTHWEST
A Coruña 1.009 (1.003, 1.015)
Lugo 1.026 (1.016, 1.036)
Ourense 1.026 (1.016, 1.036)
Pontevedra 1.017 (1.009, 1.025)
Leon 1.013 (1.003, 1.023)
Zamora 1.024 (1.008, 1.040)
Subtotal 1.018 (1.012, 1.025)
NORTH
Alava 1.018 (1.004, 1.032)
Guipuzcoa 1.014 (1.004, 1.024)
Navarra 1.011 (1.001, 1.021)
Subtotal 1.014 (1.007, 1.020)
NORTHEAST
Teruel 1.024 (1.006, 1.043)
Zaragoza 1.008 (1.000, 1.016)
Barcelona 1.008 (1.004, 1.012)
Girona 1.012 (1.002, 1.022)
Lleida 1.012 (1.002, 1.022)
Tarragona 1.009 (1.001, 1.017)
Subtotal 1.009 (1.006, 1.012)
Overall 1.014 (1.012, 1.016)
NOTE: Weights are from random effects analysis
.959
1
1.04

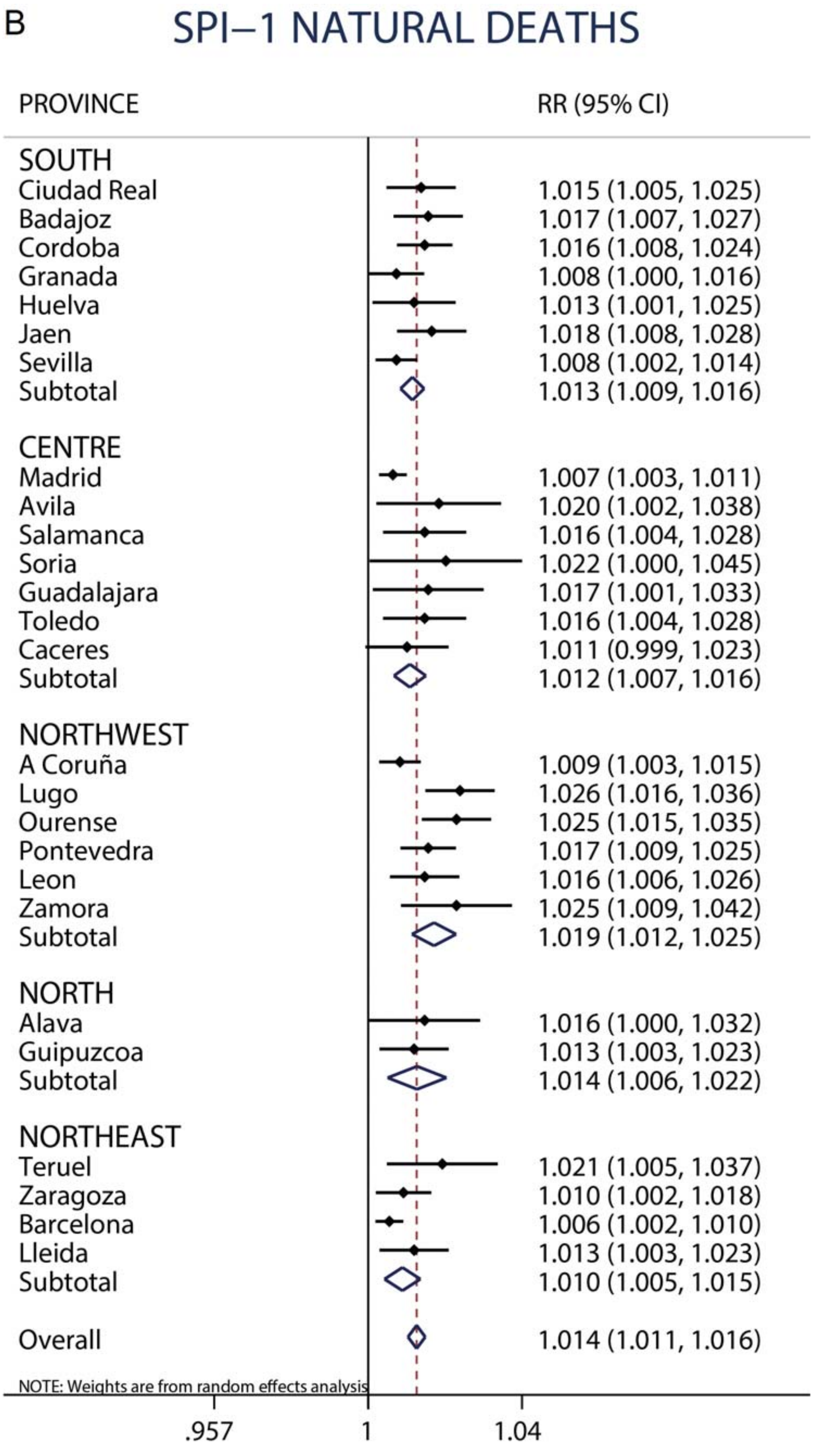
B
SPI−1 NATURAL DEATHS
PROVINCE
RR (95% CI)
SOUTH
Ciudad Real 1.015 (1.005, 1.025)
Badajoz 1.017 (1.007, 1.027)
Cordoba 1.016 (1.008, 1.024)
Granada 1.008 (1.000, 1.016)
Huelva 1.013 (1.001, 1.025)
Jaen 1.018 (1.008, 1.028)
Sevilla 1.008 (1.002, 1.014)
Subtotal 1.013 (1.009, 1.016)
CENTRE
Madrid 1.007 (1.003, 1.011)
Avila 1.020 (1.002, 1.038)
Salamanca 1.016 (1.004, 1.028)
Soria 1.022 (1.000, 1.045)
Guadalajara 1.017 (1.001, 1.033)
Toledo 1.016 (1.004, 1.028)
Caceres 1.011 (0.999, 1.023)
Subtotal 1.012 (1.007, 1.016)
NORTHWEST
A Coruña 1.009 (1.003, 1.015)
Lugo 1.026 (1.016, 1.036)
Ourense 1.025 (1.015, 1.035)
Pontevedra 1.017 (1.009, 1.025)
Leon 1.016 (1.006, 1.026)
Zamora 1.025 (1.009, 1.042)
Subtotal 1.019 (1.012, 1.025)
NORTH
Alava 1.016 (1.000, 1.032)
Guipuzcoa 1.013 (1.003, 1.023)
Subtotal 1.014 (1.006, 1.022)
NORTHEAST
Teruel 1.021 (1.005, 1.037)
Zaragoza 1.010 (1.002, 1.018)
Barcelona 1.006 (1.002, 1.010)
Lleida 1.013 (1.003, 1.023)
Subtotal 1.010 (1.005, 1.015)
Overall 1.014 (1.011, 1.016)
NOTE: Weights are from random effects analysis
.957
1
1.04

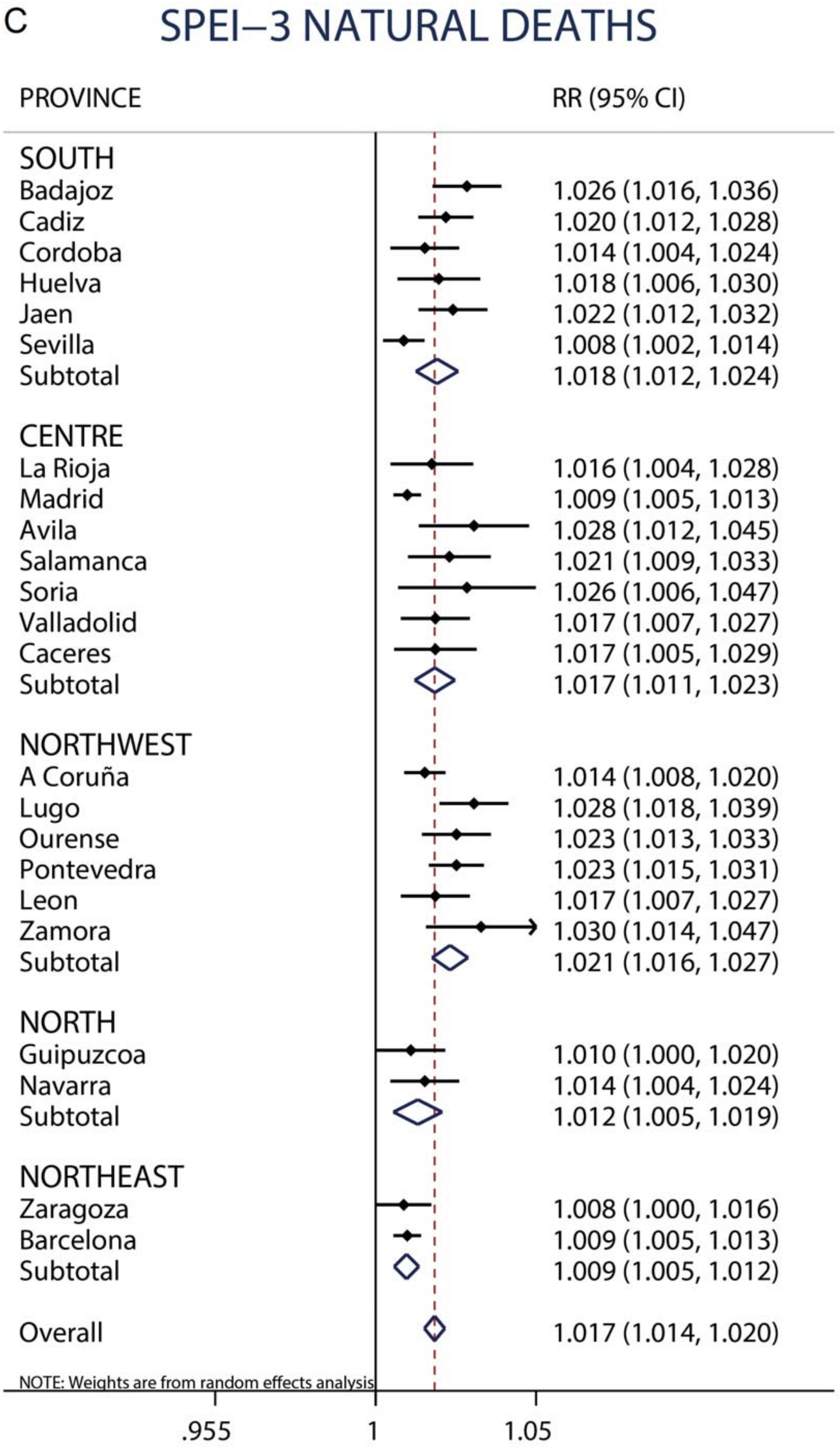
C
SPEI–3 NATURAL DEATHS
PROVINCE
RR (95% CI)
SOUTH
Badajoz 1.026 (1.016, 1.036)
Cadiz 1.020 (1.012, 1.028)
Cordoba 1.014 (1.004, 1.024)
Huelva 1.018 (1.006, 1.030)
Jaen 1.022 (1.012, 1.032)
Sevilla 1.008 (1.002, 1.014)
Subtotal 1.018 (1.012, 1.024)
CENTRE
La Rioja 1.016 (1.004, 1.028)
Madrid 1.009 (1.005, 1.013)
Avila 1.028 (1.012, 1.045)
Salamanca 1.021 (1.009, 1.033)
Soria 1.026 (1.006, 1.047)
Valladolid 1.017 (1.007, 1.027)
Caceres 1.017 (1.005, 1.029)
Subtotal 1.017 (1.011, 1.023)
NORTHWEST
A Coruña 1.014 (1.008, 1.020)
Lugo 1.028 (1.018, 1.039)
Ourense 1.023 (1.013, 1.033)
Pontevedra 1.023 (1.015, 1.031)
Leon 1.017 (1.007, 1.027)
Zamora 1.030 (1.014, 1.047)
Subtotal 1.021 (1.016, 1.027)
NORTH
Guipuzcoa 1.010 (1.000, 1.020)
Navarra 1.014 (1.004, 1.024)
Subtotal 1.012 (1.005, 1.019)
NORTHEAST
Zaragoza 1.008 (1.000, 1.016)
Barcelona 1.009 (1.005, 1.013)
Subtotal 1.009 (1.005, 1.012)
Overall 1.017 (1.014, 1.020)
NOTE: Weights are from random effects analysis
.955
1
1.05

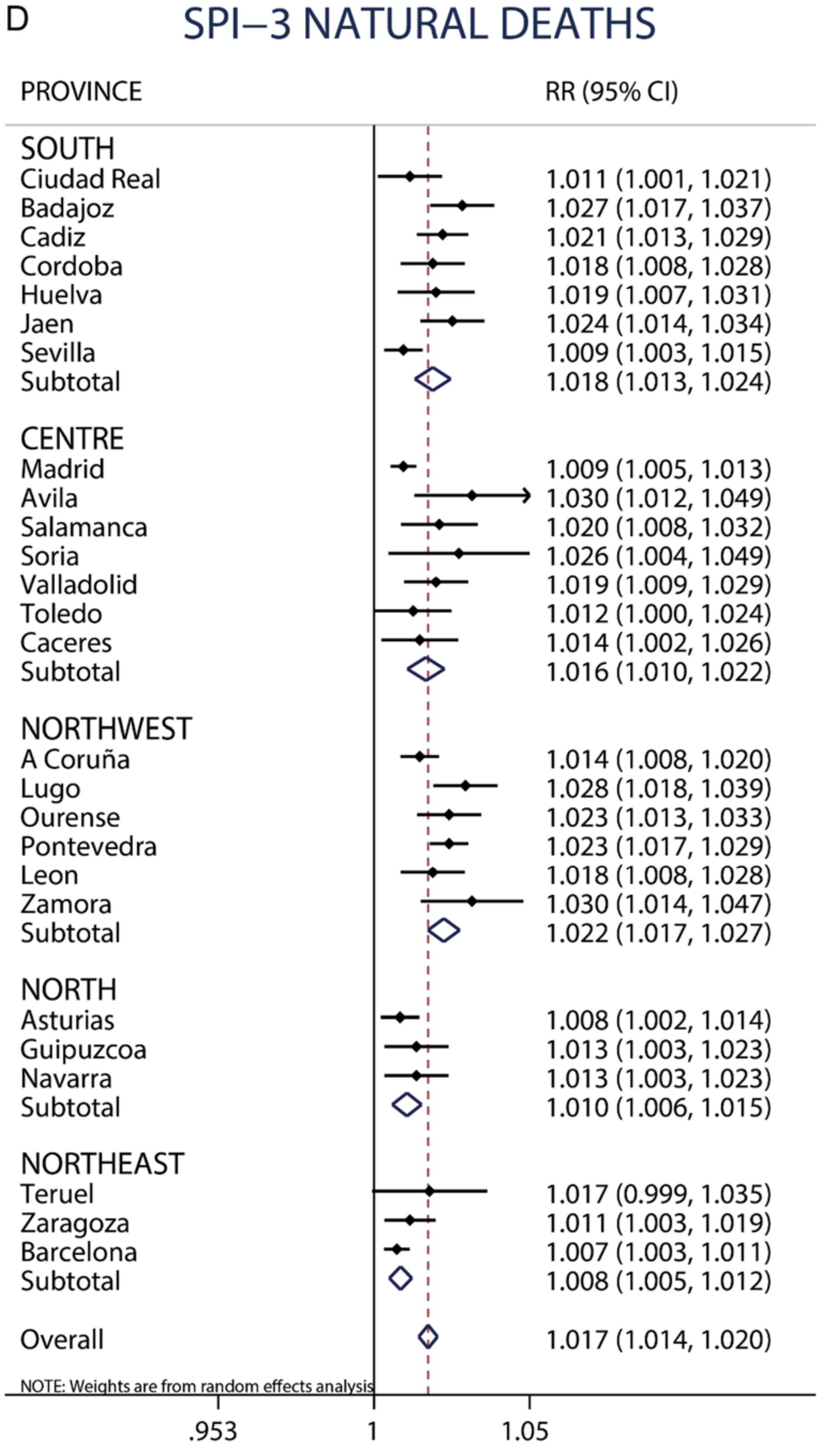
D
SPI–3 NATURAL DEATHS
PROVINCE
RR (95% CI)
SOUTH
Ciudad Real 1.011 (1.001, 1.021)
Badajoz 1.027 (1.017, 1.037)
Cadiz 1.021 (1.013, 1.029)
Cordoba 1.018 (1.008, 1.028)
Huelva 1.019 (1.007, 1.031)
Jaen 1.024 (1.014, 1.034)
Sevilla 1.009 (1.003, 1.015)
Subtotal 1.018 (1.013, 1.024)
CENTRE
Madrid 1.009 (1.005, 1.013)
Avila 1.030 (1.012, 1.049)
Salamanca 1.020 (1.008, 1.032)
Soria 1.026 (1.004, 1.049)
Valladolid 1.019 (1.009, 1.029)
Toledo 1.012 (1.000, 1.024)
Caceres 1.014 (1.002, 1.026)
Subtotal 1.016 (1.010, 1.022)
NORTHWEST
A Coruña 1.014 (1.008, 1.020)
Lugo 1.028 (1.018, 1.039)
Ourense 1.023 (1.013, 1.033)
Pontevedra 1.023 (1.017, 1.029)
Leon 1.018 (1.008, 1.028)
Zamora 1.030 (1.014, 1.047)
Subtotal 1.022 (1.017, 1.027)
NORTH
Asturias 1.008 (1.002, 1.014)
Guipuzcoa 1.013 (1.003, 1.023)
Navarra 1.013 (1.003, 1.023)
Subtotal 1.010 (1.006, 1.015)
NORTHEAST
Teruel 1.017 (0.999, 1.035)
Zaragoza 1.011 (1.003, 1.019)
Barcelona 1.007 (1.003, 1.011)
Subtotal 1.008 (1.005, 1.012)
Overall 1.017 (1.014, 1.020)
NOTE: Weights are from random effects analysis
.953
1
1.05

Figure S4. Forest plots of the relative risks (RR) values of daily natural mortality associated with droughts by the climatic regionalization. **A** and **B**: Droughts measured by the Standardized Precipitation Evapotranspiration Index (SPEI) and the Standardized Precipitation Index (SPI) obtained at one month of drought accumulation (SPEI-1 and SPI-1, respectively). **C** and **D**: As per A and B, but for three months of accumulation (SPEI-3 and SPI-3, respectively). Only provinces with a statistically significant association ($p<0.05$) between drought indices and natural deaths are shown. Provincial RR data obtained with the use of both SPEI-1 and SPI-1 from Salvador et al., 2020.

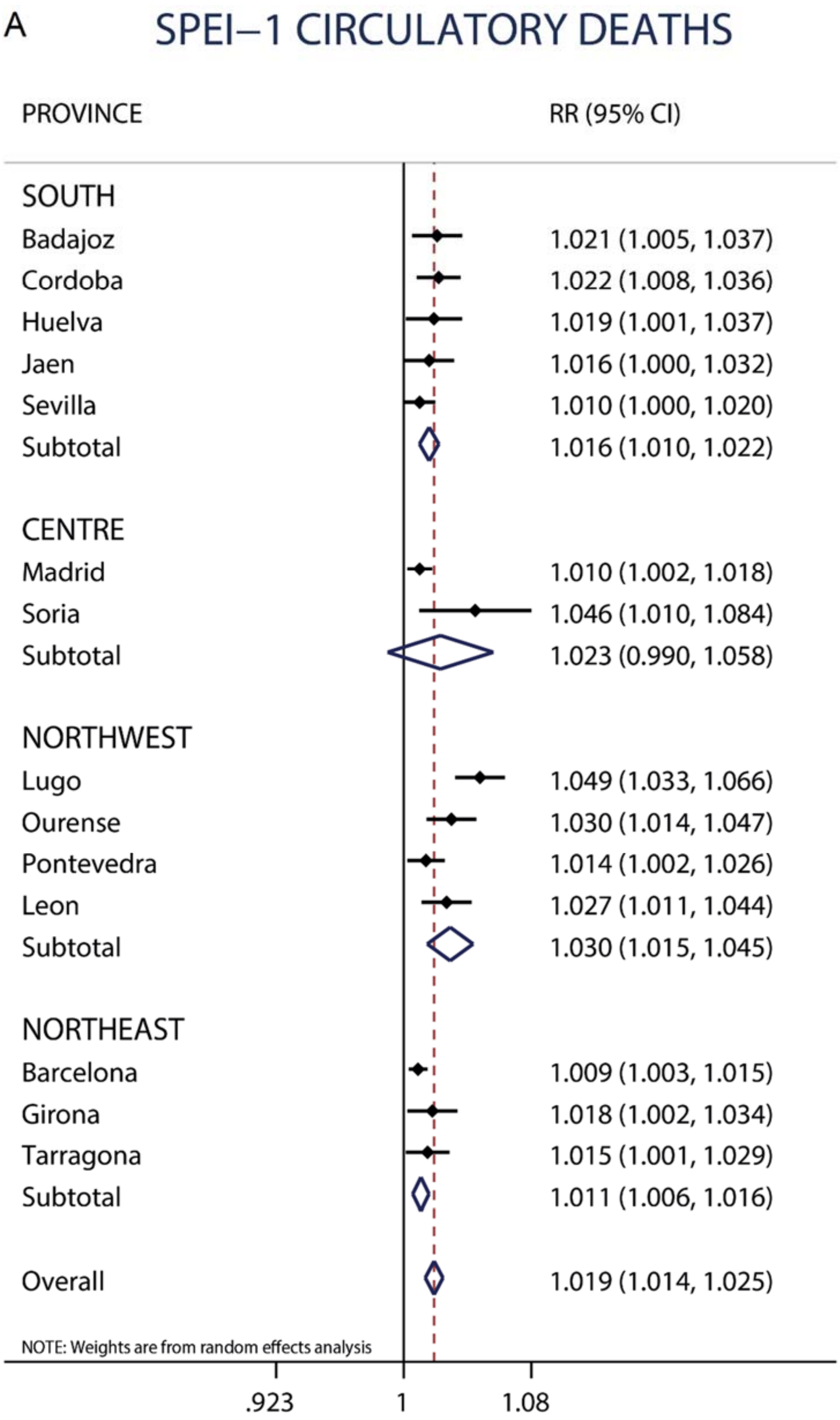
A
SPEI–1 CIRCULATORY DEATHS
PROVINCE
RR (95% CI)
SOUTH
Badajoz 1.021 (1.005, 1.037)
Cordoba 1.022 (1.008, 1.036)
Huelva 1.019 (1.001, 1.037)
Jaen 1.016 (1.000, 1.032)
Sevilla 1.010 (1.000, 1.020)
Subtotal 1.016 (1.010, 1.022)
CENTRE
Madrid 1.010 (1.002, 1.018)
Soria 1.046 (1.010, 1.084)
Subtotal 1.023 (0.990, 1.058)
NORTHWEST
Lugo 1.049 (1.033, 1.066)
Ourense 1.030 (1.014, 1.047)
Pontevedra 1.014 (1.002, 1.026)
Leon 1.027 (1.011, 1.044)
Subtotal 1.030 (1.015, 1.045)
NORTHEAST
Barcelona 1.009 (1.003, 1.015)
Girona 1.018 (1.002, 1.034)
Tarragona 1.015 (1.001, 1.029)
Subtotal 1.011 (1.006, 1.016)
Overall 1.019 (1.014, 1.025)
NOTE: Weights are from random effects analysis
.923
1
1.08

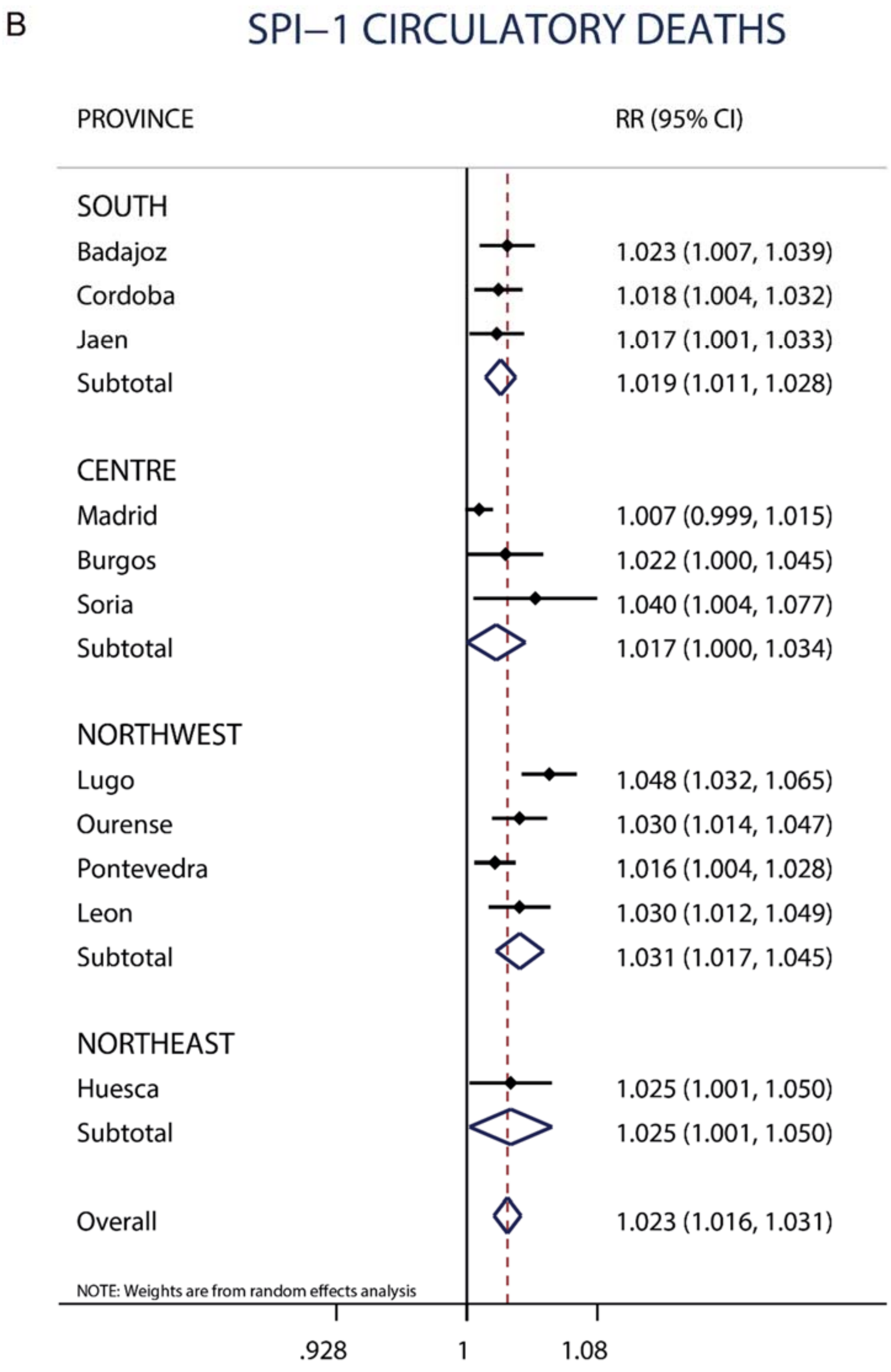
B
SPI–1 CIRCULATORY DEATHS
PROVINCE
RR (95% CI)
SOUTH
Badajoz 1.023 (1.007, 1.039)
Cordoba 1.018 (1.004, 1.032)
Jaen 1.017 (1.001, 1.033)
Subtotal 1.019 (1.011, 1.028)
CENTRE
Madrid 1.007 (0.999, 1.015)
Burgos 1.022 (1.000, 1.045)
Soria 1.040 (1.004, 1.077)
Subtotal 1.017 (1.000, 1.034)
NORTHWEST
Lugo 1.048 (1.032, 1.065)
Ourense 1.030 (1.014, 1.047)
Pontevedra 1.016 (1.004, 1.028)
Leon 1.030 (1.012, 1.049)
Subtotal 1.031 (1.017, 1.045)
NORTHEAST
Huesca 1.025 (1.001, 1.050)
Subtotal 1.025 (1.001, 1.050)
Overall 1.023 (1.016, 1.031)
NOTE: Weights are from random effects analysis
.928
1
1.08

C

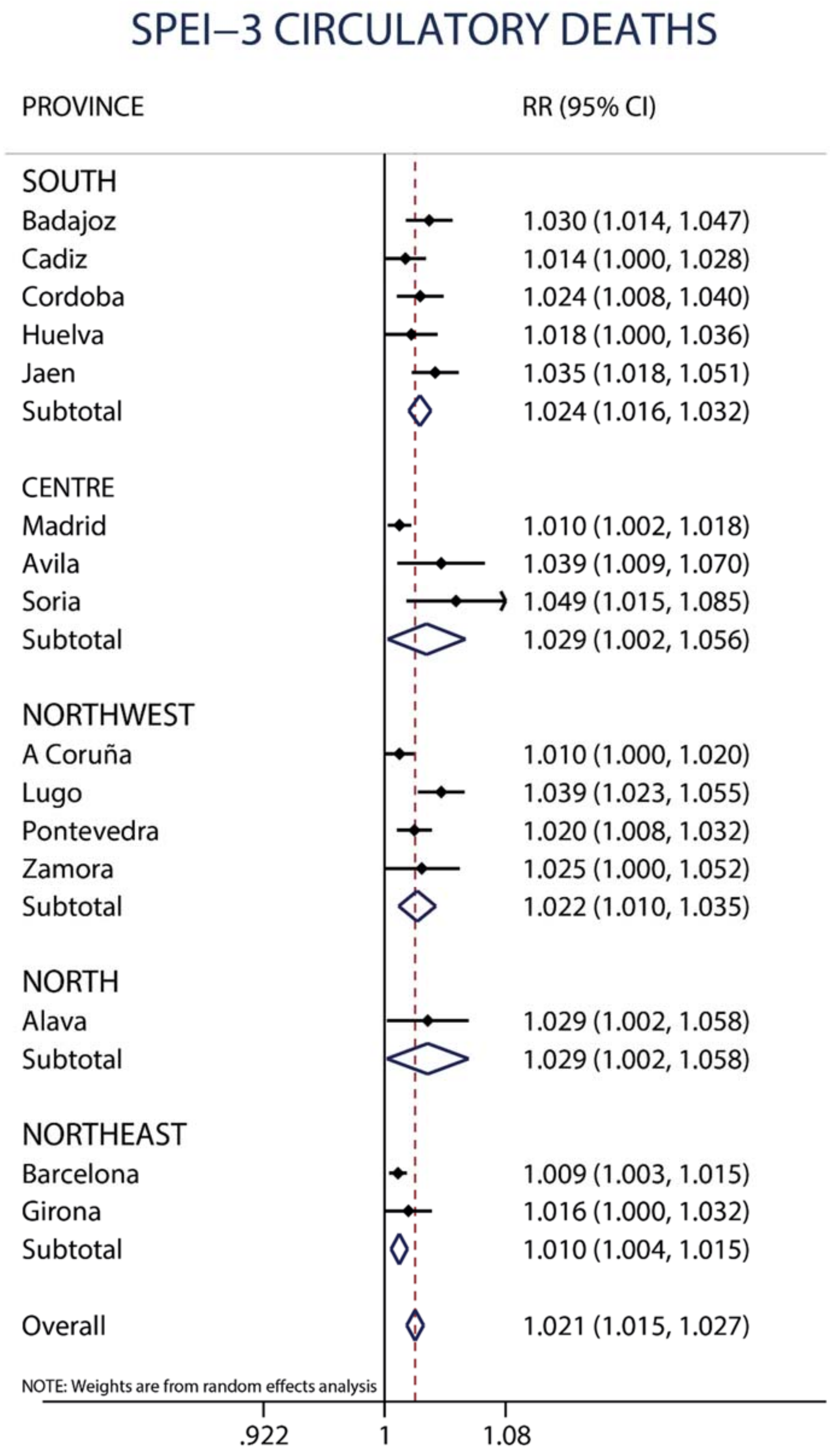
SPEI–3 CIRCULATORY DEATHS
PROVINCE
RR (95% CI)
SOUTH
Badajoz 1.030 (1.014, 1.047)
Cadiz 1.014 (1.000, 1.028)
Cordoba 1.024 (1.008, 1.040)
Huelva 1.018 (1.000, 1.036)
Jaen 1.035 (1.018, 1.051)
Subtotal 1.024 (1.016, 1.032)
CENTRE
Madrid 1.010 (1.002, 1.018)
Avila 1.039 (1.009, 1.070)
Soria 1.049 (1.015, 1.085)
Subtotal 1.029 (1.002, 1.056)
NORTHWEST
A Coruña 1.010 (1.000, 1.020)
Lugo 1.039 (1.023, 1.055)
Pontevedra 1.020 (1.008, 1.032)
Zamora 1.025 (1.000, 1.052)
Subtotal 1.022 (1.010, 1.035)
NORTH
Alava 1.029 (1.002, 1.058)
Subtotal 1.029 (1.002, 1.058)
NORTHEAST
Barcelona 1.009 (1.003, 1.015)
Girona 1.016 (1.000, 1.032)
Subtotal 1.010 (1.004, 1.015)
Overall 1.021 (1.015, 1.027)
NOTE: Weights are from random effects analysis
.922
1
1.08

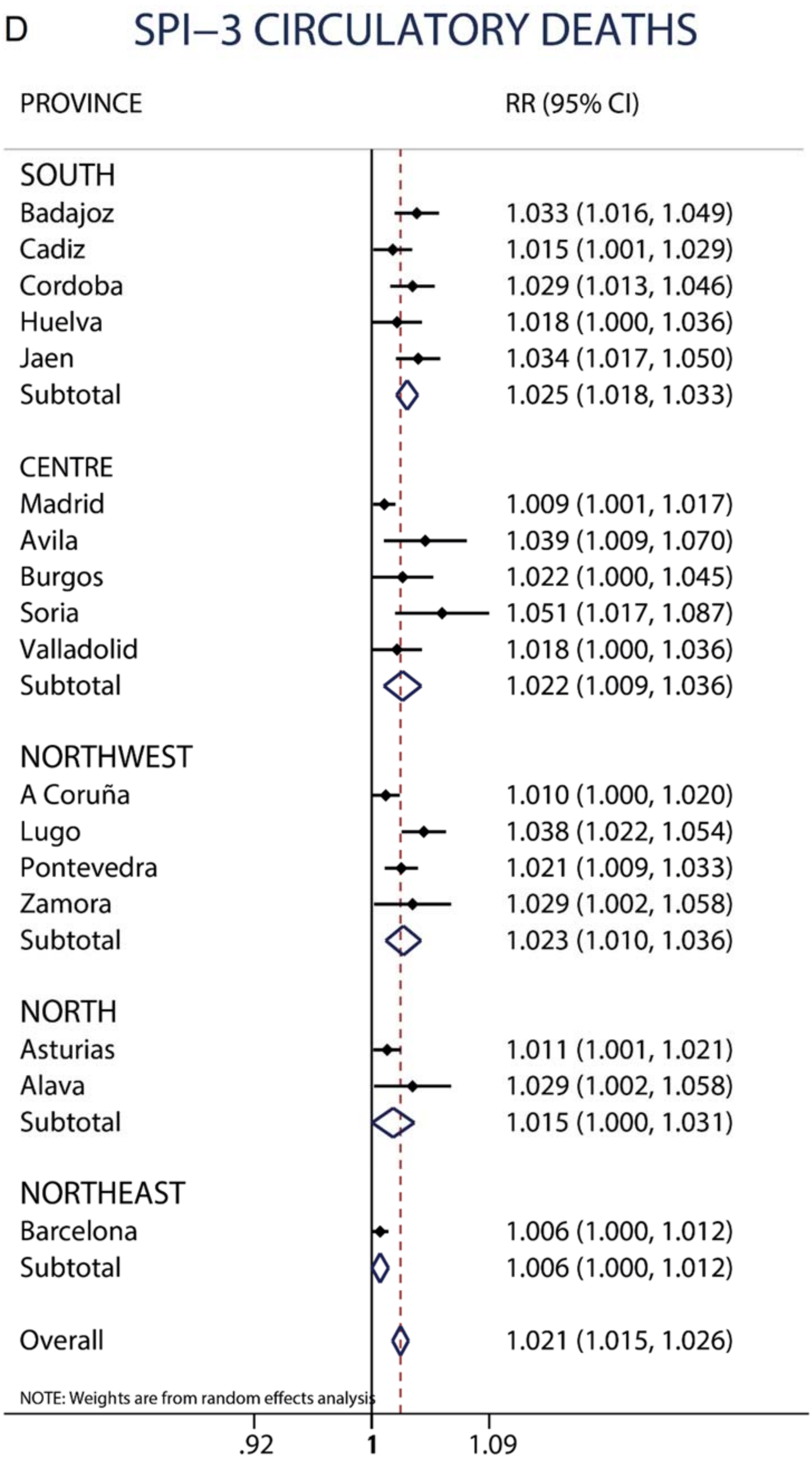
D
SPI–3 CIRCULATORY DEATHS
PROVINCE
RR (95% CI)
SOUTH
Badajoz 1.033 (1.016, 1.049)
Cadiz 1.015 (1.001, 1.029)
Cordoba 1.029 (1.013, 1.046)
Huelva 1.018 (1.000, 1.036)
Jaen 1.034 (1.017, 1.050)
Subtotal 1.025 (1.018, 1.033)
CENTRE
Madrid 1.009 (1.001, 1.017)
Avila 1.039 (1.009, 1.070)
Burgos 1.022 (1.000, 1.045)
Soria 1.051 (1.017, 1.087)
Valladolid 1.018 (1.000, 1.036)
Subtotal 1.022 (1.009, 1.036)
NORTHWEST
A Coruña 1.010 (1.000, 1.020)
Lugo 1.038 (1.022, 1.054)
Pontevedra 1.021 (1.009, 1.033)
Zamora 1.029 (1.002, 1.058)
Subtotal 1.023 (1.010, 1.036)
NORTH
Asturias 1.011 (1.001, 1.021)
Alava 1.029 (1.002, 1.058)
Subtotal 1.015 (1.000, 1.031)
NORTHEAST
Barcelona 1.006 (1.000, 1.012)
Subtotal 1.006 (1.000, 1.012)
Overall 1.021 (1.015, 1.026)
NOTE: Weights are from random effects analysis
.92
1
1.09

Figure S5. Forest plots of the relative risks (RR) values of daily circulatory mortality associated with droughts by the climatic regionalization. **A** and **B**: Droughts measured by the Standardized Precipitation Evapotranspiration Index (SPEI) and the Standardized Precipitation Index (SPI) obtained at one month of drought accumulation (SPEI-1 and SPI-1, respectively). **C** and **D**: As per A and B, but for three months of accumulation (SPEI-3 and SPI-3, respectively). Only provinces with a statistically significant association ($p<0.05$) between drought indices and circulatory deaths are shown. Provincial RR data obtained with the use of both SPEI-1 and SPI-1 from Salvador et al., 2020.

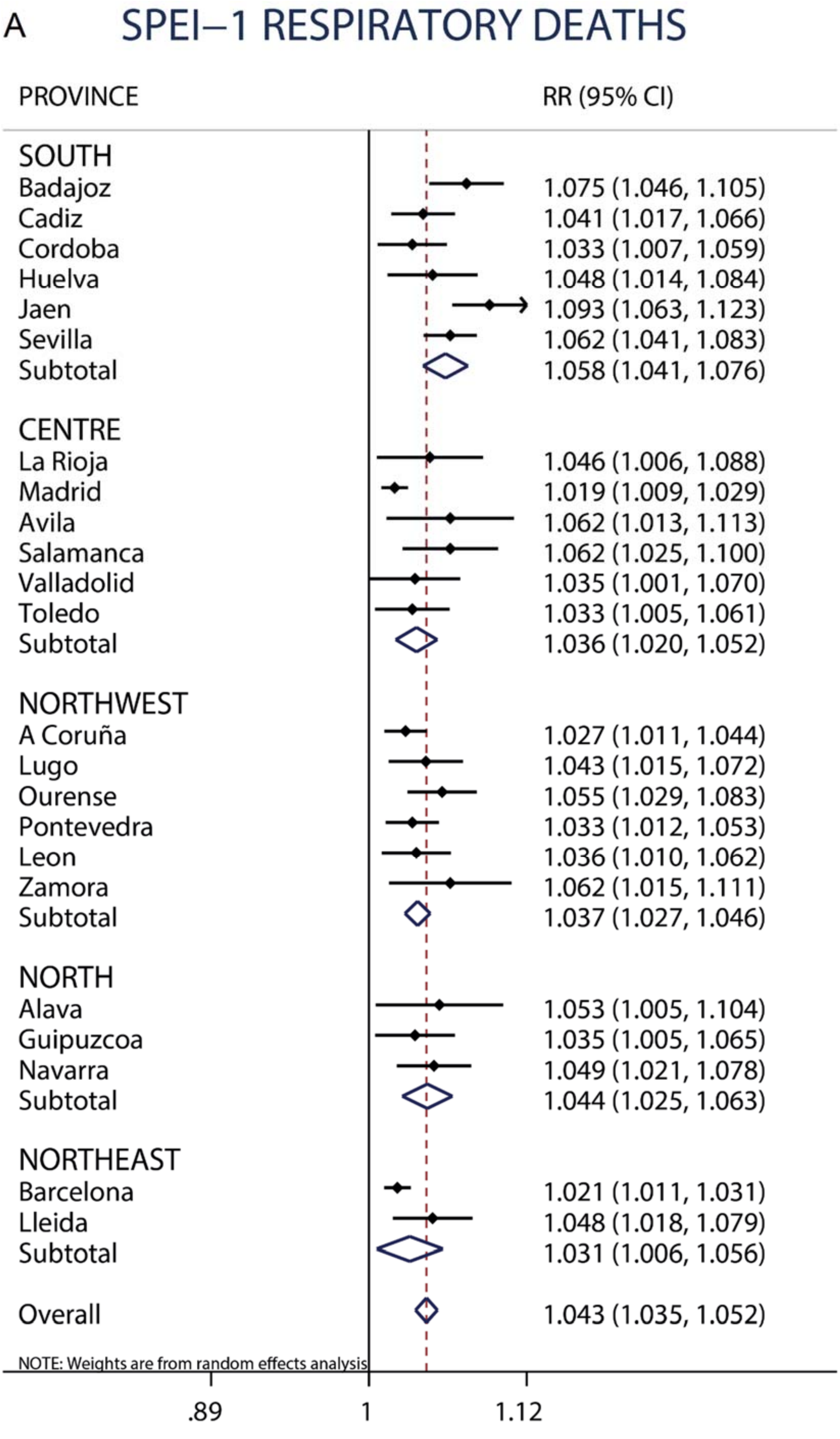
A
SPEI–1 RESPIRATORY DEATHS
PROVINCE
RR (95% CI)
SOUTH
Badajoz 1.075 (1.046, 1.105)
Cadiz 1.041 (1.017, 1.066)
Cordoba 1.033 (1.007, 1.059)
Huelva 1.048 (1.014, 1.084)
Jaen 1.093 (1.063, 1.123)
Sevilla 1.062 (1.041, 1.083)
Subtotal 1.058 (1.041, 1.076)
CENTRE
La Rioja 1.046 (1.006, 1.088)
Madrid 1.019 (1.009, 1.029)
Avila 1.062 (1.013, 1.113)
Salamanca 1.062 (1.025, 1.100)
Valladolid 1.035 (1.001, 1.070)
Toledo 1.033 (1.005, 1.061)
Subtotal 1.036 (1.020, 1.052)
NORTHWEST
A Coruña 1.027 (1.011, 1.044)
Lugo 1.043 (1.015, 1.072)
Ourense 1.055 (1.029, 1.083)
Pontevedra 1.033 (1.012, 1.053)
Leon 1.036 (1.010, 1.062)
Zamora 1.062 (1.015, 1.111)
Subtotal 1.037 (1.027, 1.046)
NORTH
Alava 1.053 (1.005, 1.104)
Guipuzcoa 1.035 (1.005, 1.065)
Navarra 1.049 (1.021, 1.078)
Subtotal 1.044 (1.025, 1.063)
NORTHEAST
Barcelona 1.021 (1.011, 1.031)
Lleida 1.048 (1.018, 1.079)
Subtotal 1.031 (1.006, 1.056)
Overall 1.043 (1.035, 1.052)
NOTE: Weights are from random effects analysis
.89
1
1.12

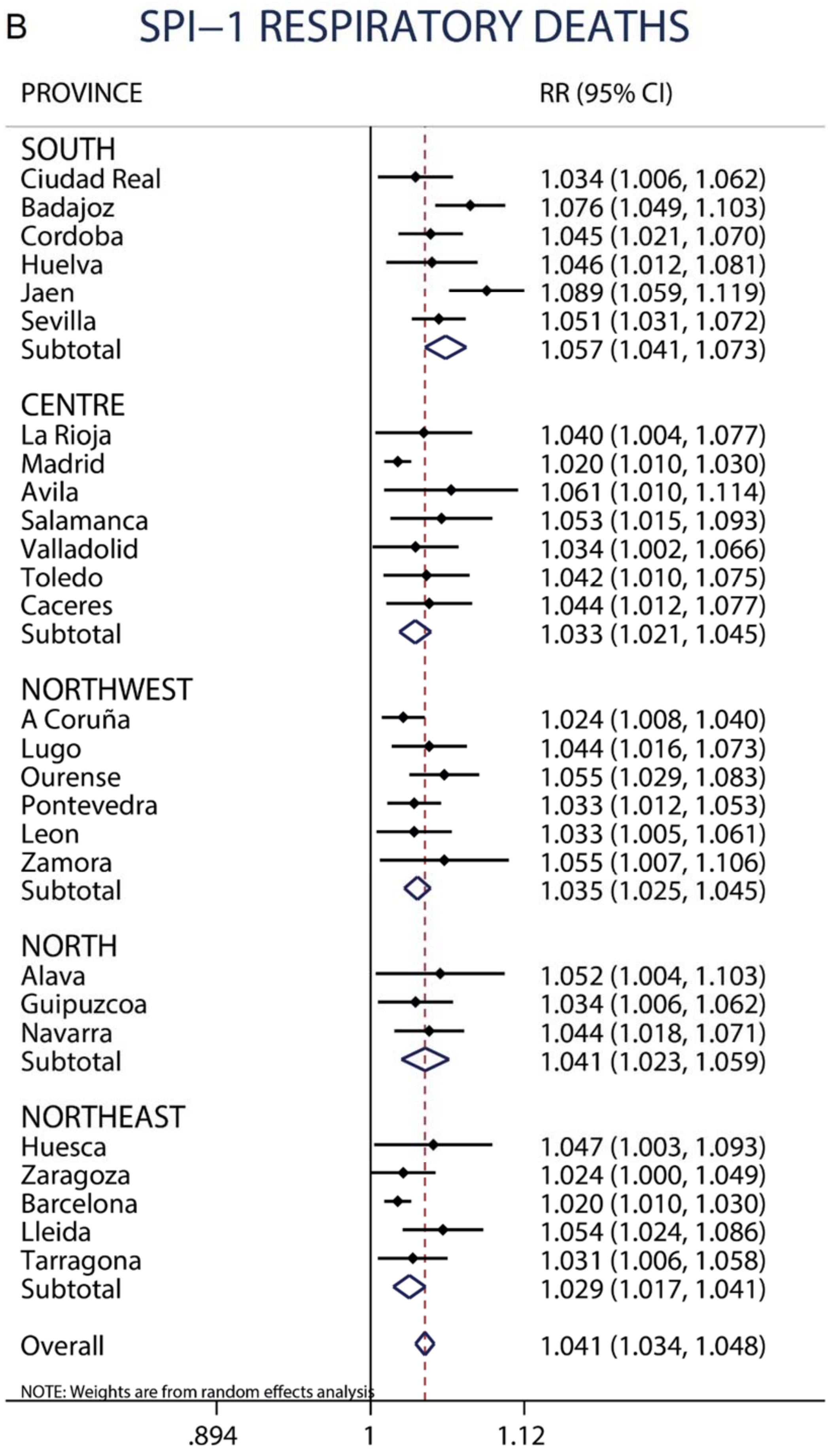
B
SPI–1 RESPIRATORY DEATHS
PROVINCE
RR (95% CI)
SOUTH
Ciudad Real 1.034 (1.006, 1.062)
Badajoz 1.076 (1.049, 1.103)
Cordoba 1.045 (1.021, 1.070)
Huelva 1.046 (1.012, 1.081)
Jaen 1.089 (1.059, 1.119)
Sevilla 1.051 (1.031, 1.072)
Subtotal 1.057 (1.041, 1.073)
CENTRE
La Rioja 1.040 (1.004, 1.077)
Madrid 1.020 (1.010, 1.030)
Avila 1.061 (1.010, 1.114)
Salamanca 1.053 (1.015, 1.093)
Valladolid 1.034 (1.002, 1.066)
Toledo 1.042 (1.010, 1.075)
Caceres 1.044 (1.012, 1.077)
Subtotal 1.033 (1.021, 1.045)
NORTHWEST
A Coruña 1.024 (1.008, 1.040)
Lugo 1.044 (1.016, 1.073)
Ourense 1.055 (1.029, 1.083)
Pontevedra 1.033 (1.012, 1.053)
Leon 1.033 (1.005, 1.061)
Zamora 1.055 (1.007, 1.106)
Subtotal 1.035 (1.025, 1.045)
NORTH
Alava 1.052 (1.004, 1.103)
Guipuzcoa 1.034 (1.006, 1.062)
Navarra 1.044 (1.018, 1.071)
Subtotal 1.041 (1.023, 1.059)
NORTHEAST
Huesca 1.047 (1.003, 1.093)
Zaragoza 1.024 (1.000, 1.049)
Barcelona 1.020 (1.010, 1.030)
Lleida 1.054 (1.024, 1.086)
Tarragona 1.031 (1.006, 1.058)
Subtotal 1.029 (1.017, 1.041)
Overall 1.041 (1.034, 1.048)
NOTE: Weights are from random effects analysis
.894
1
1.12

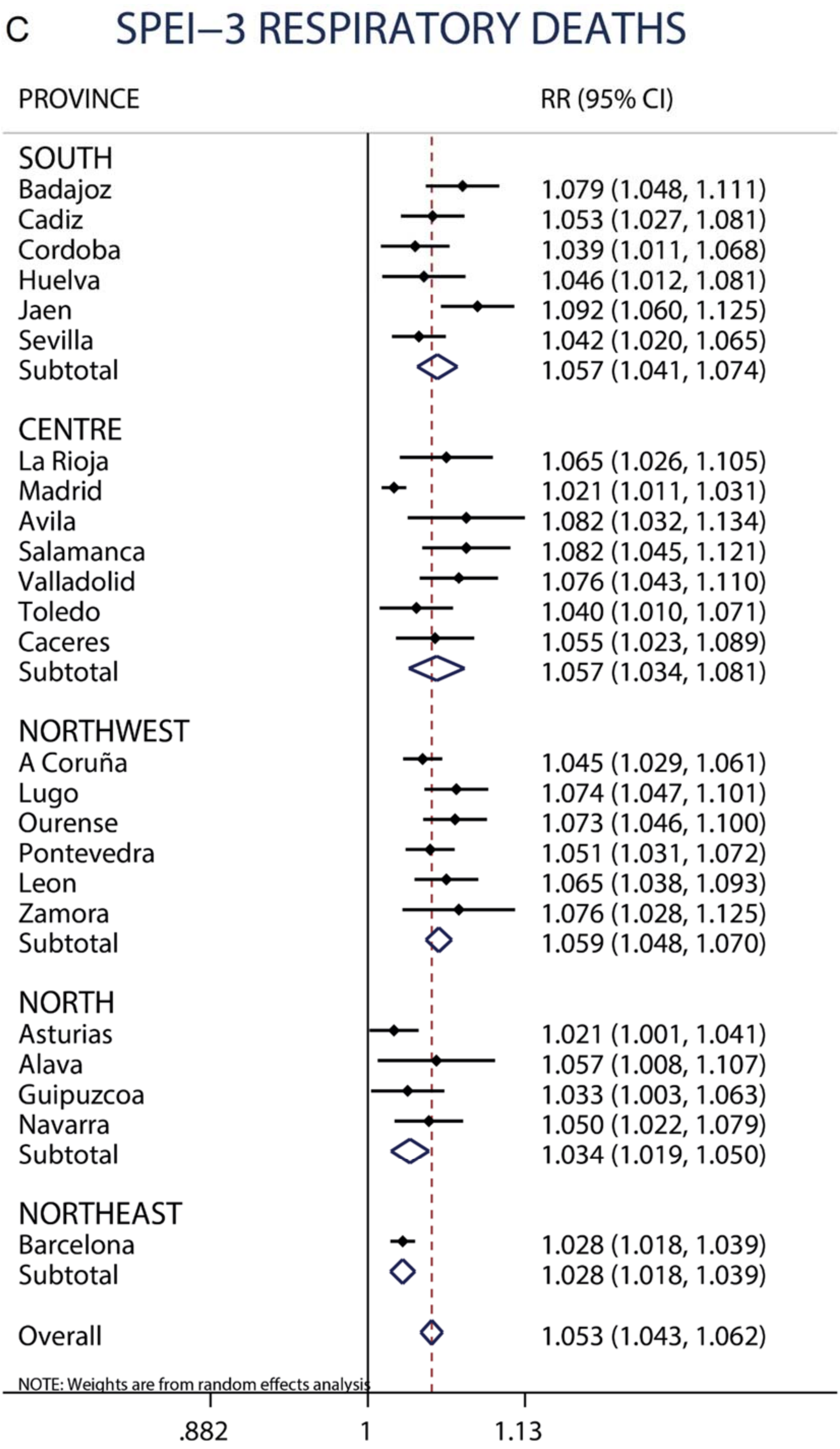
C
SPEI–3 RESPIRATORY DEATHS
PROVINCE
RR (95% CI)
SOUTH
Badajoz 1.079 (1.048, 1.111)
Cadiz 1.053 (1.027, 1.081)
Cordoba 1.039 (1.011, 1.068)
Huelva 1.046 (1.012, 1.081)
Jaen 1.092 (1.060, 1.125)
Sevilla 1.042 (1.020, 1.065)
Subtotal 1.057 (1.041, 1.074)
CENTRE
La Rioja 1.065 (1.026, 1.105)
Madrid 1.021 (1.011, 1.031)
Avila 1.082 (1.032, 1.134)
Salamanca 1.082 (1.045, 1.121)
Valladolid 1.076 (1.043, 1.110)
Toledo 1.040 (1.010, 1.071)
Caceres 1.055 (1.023, 1.089)
Subtotal 1.057 (1.034, 1.081)
NORTHWEST
A Coruña 1.045 (1.029, 1.061)
Lugo 1.074 (1.047, 1.101)
Ourense 1.073 (1.046, 1.100)
Pontevedra 1.051 (1.031, 1.072)
Leon 1.065 (1.038, 1.093)
Zamora 1.076 (1.028, 1.125)
Subtotal 1.059 (1.048, 1.070)
NORTH
Asturias 1.021 (1.001, 1.041)
Alava 1.057 (1.008, 1.107)
Guipuzcoa 1.033 (1.003, 1.063)
Navarra 1.050 (1.022, 1.079)
Subtotal 1.034 (1.019, 1.050)
NORTHEAST
Barcelona 1.028 (1.018, 1.039)
Subtotal 1.028 (1.018, 1.039)
Overall 1.053 (1.043, 1.062)
NOTE: Weights are from random effects analysis
.882
1
1.13

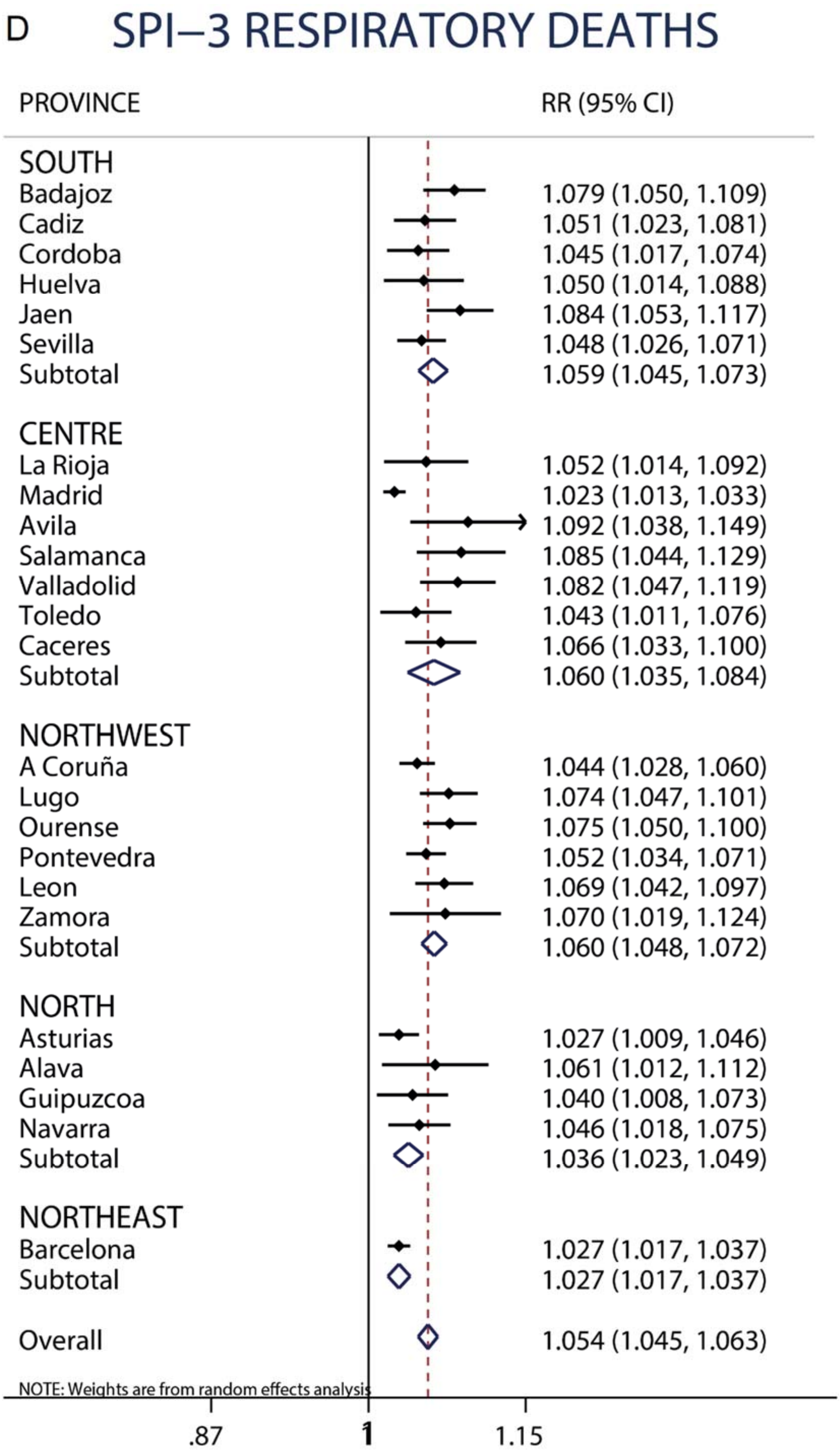
D
SPI–3 RESPIRATORY DEATHS
PROVINCE
RR (95% CI)
SOUTH
Badajoz 1.079 (1.050, 1.109)
Cadiz 1.051 (1.023, 1.081)
Cordoba 1.045 (1.017, 1.074)
Huelva 1.050 (1.014, 1.088)
Jaen 1.084 (1.053, 1.117)
Sevilla 1.048 (1.026, 1.071)
Subtotal 1.059 (1.045, 1.073)
CENTRE
La Rioja 1.052 (1.014, 1.092)
Madrid 1.023 (1.013, 1.033)
Avila 1.092 (1.038, 1.149)
Salamanca 1.085 (1.044, 1.129)
Valladolid 1.082 (1.047, 1.119)
Toledo 1.043 (1.011, 1.076)
Caceres 1.066 (1.033, 1.100)
Subtotal 1.060 (1.035, 1.084)
NORTHWEST
A Coruña 1.044 (1.028, 1.060)
Lugo 1.074 (1.047, 1.101)
Ourense 1.075 (1.050, 1.100)
Pontevedra 1.052 (1.034, 1.071)
Leon 1.069 (1.042, 1.097)
Zamora 1.070 (1.019, 1.124)
Subtotal 1.060 (1.048, 1.072)
NORTH
Asturias 1.027 (1.009, 1.046)
Alava 1.061 (1.012, 1.112)
Guipuzcoa 1.040 (1.008, 1.073)
Navarra 1.046 (1.018, 1.075)
Subtotal 1.036 (1.023, 1.049)
NORTHEAST
Barcelona 1.027 (1.017, 1.037)
Subtotal 1.027 (1.017, 1.037)
Overall 1.054 (1.045, 1.063)
NOTE: Weights are from random effects analysis
.87
1
1.15

Figure S6. Forest plots of the relative risks (RR) values of daily respiratory mortality associated with droughts by the climatic regionalization. **A** and **B**: Droughts measured by the Standardized Precipitation Evapotranspiration Index (SPEI) and the Standardized Precipitation Index (SPI) obtained at one month of drought accumulation (SPEI-1 and SPI-1, respectively). **C** and **D**: As per A and B, but for three months of accumulation (SPEI-3 and SPI-3, respectively). Only provinces with a statistically significant association ($p<0.05$) between drought indices and respiratory deaths are shown. Provincial RR data obtained with the use of both SPEI-1 and SPI-1 from Salvador et al., 2020.

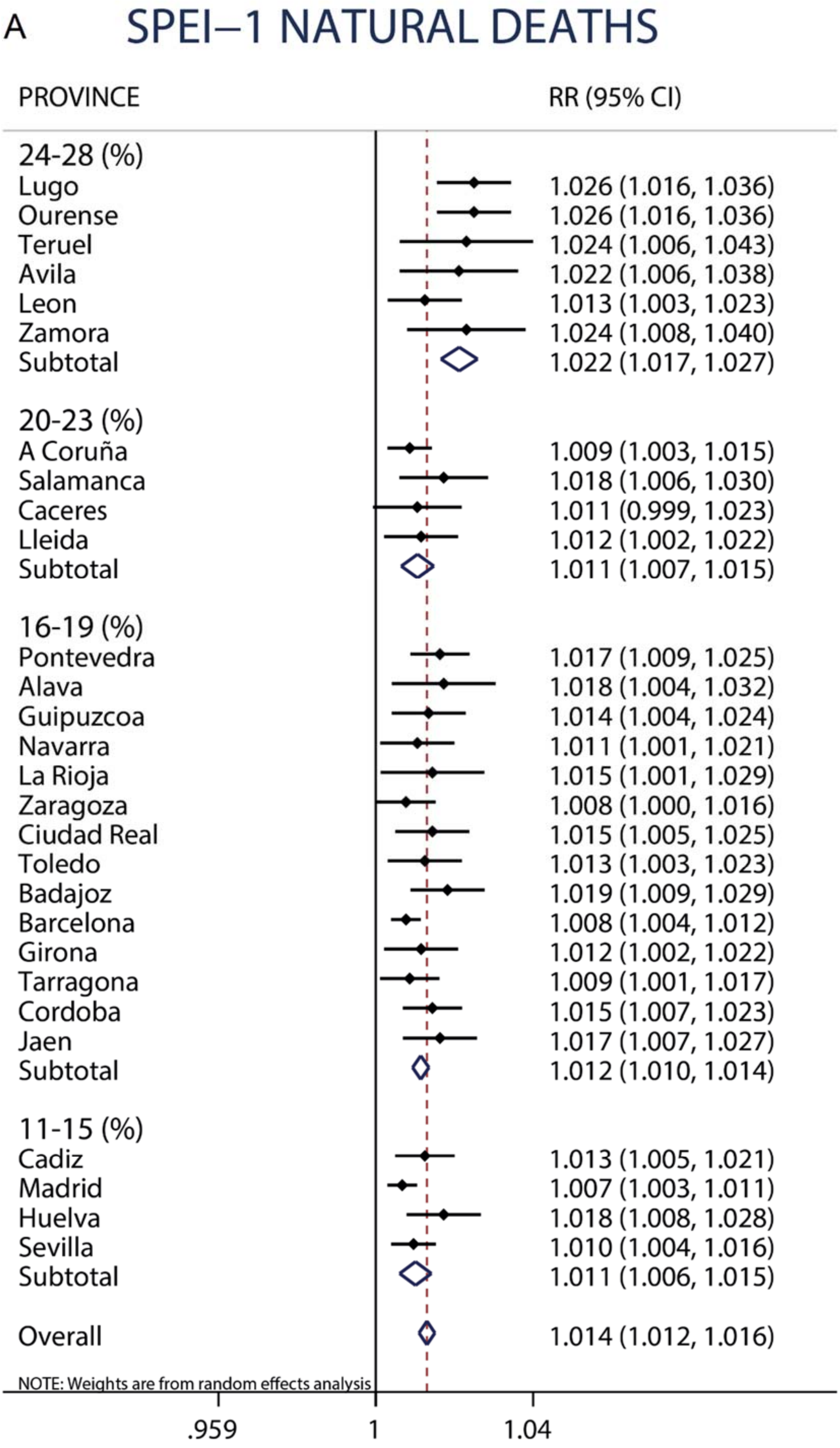
A
SPEI–1 NATURAL DEATHS
PROVINCE
RR (95% CI)
24-28 (%)
Lugo 1.026 (1.016, 1.036)
Ourense 1.026 (1.016, 1.036)
Teruel 1.024 (1.006, 1.043)
Avila 1.022 (1.006, 1.038)
Leon 1.013 (1.003, 1.023)
Zamora 1.024 (1.008, 1.040)
Subtotal 1.022 (1.017, 1.027)
20-23 (%)
A Coruña 1.009 (1.003, 1.015)
Salamanca 1.018 (1.006, 1.030)
Caceres 1.011 (0.999, 1.023)
Lleida 1.012 (1.002, 1.022)
Subtotal 1.011 (1.007, 1.015)
16-19 (%)
Pontevedra 1.017 (1.009, 1.025)
Alava 1.018 (1.004, 1.032)
Guipuzcoa 1.014 (1.004, 1.024)
Navarra 1.011 (1.001, 1.021)
La Rioja 1.015 (1.001, 1.029)
Zaragoza 1.008 (1.000, 1.016)
Ciudad Real 1.015 (1.005, 1.025)
Toledo 1.013 (1.003, 1.023)
Badajoz 1.019 (1.009, 1.029)
Barcelona 1.008 (1.004, 1.012)
Girona 1.012 (1.002, 1.022)
Tarragona 1.009 (1.001, 1.017)
Cordoba 1.015 (1.007, 1.023)
Jaen 1.017 (1.007, 1.027)
Subtotal 1.012 (1.010, 1.014)
11-15 (%)
Cadiz 1.013 (1.005, 1.021)
Madrid 1.007 (1.003, 1.011)
Huelva 1.018 (1.008, 1.028)
Sevilla 1.010 (1.004, 1.016)
Subtotal 1.011 (1.006, 1.015)
Overall 1.014 (1.012, 1.016)
NOTE: Weights are from random effects analysis
.959
1
1.04

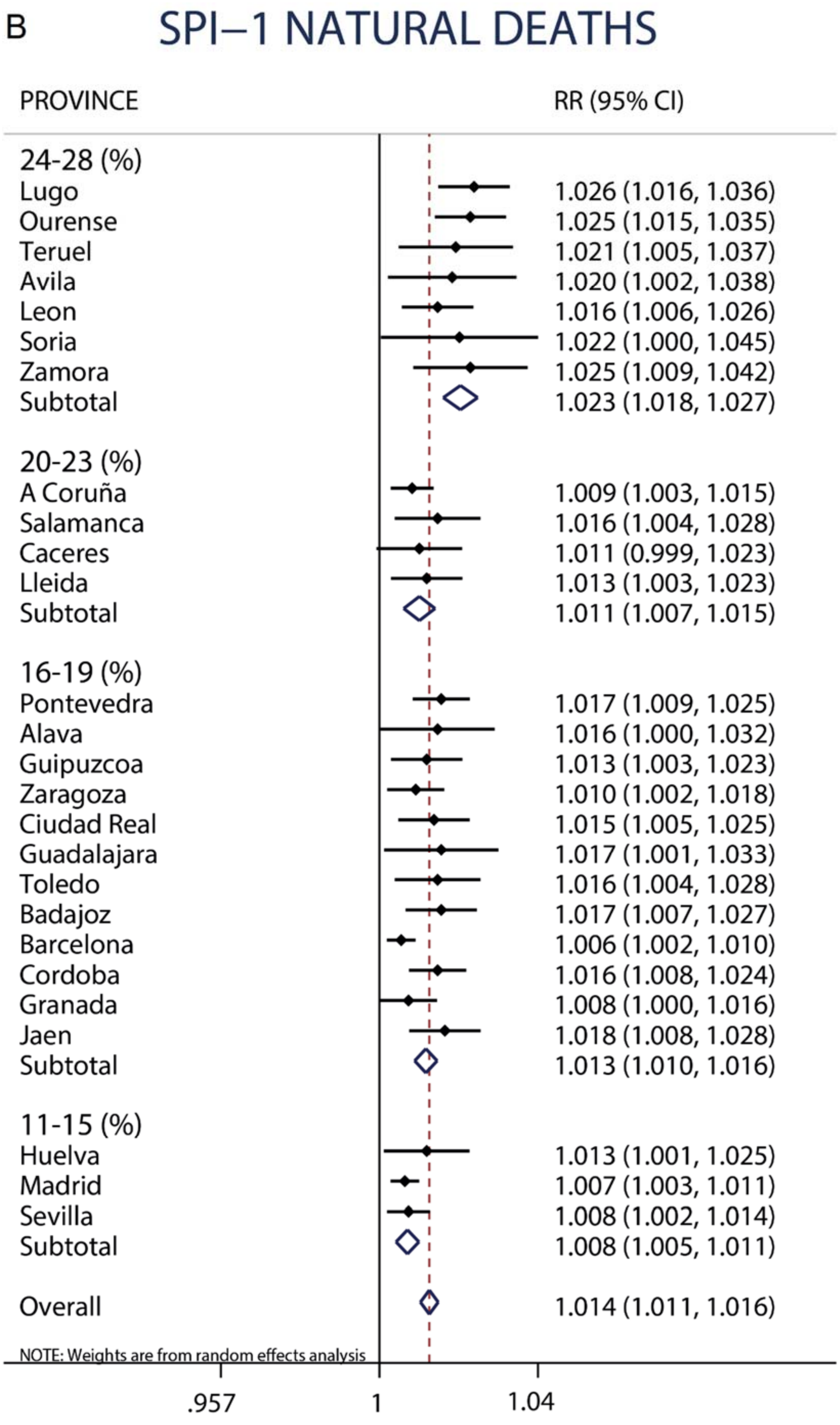
B
SPI–1 NATURAL DEATHS
PROVINCE
RR (95% CI)
24-28 (%)
Lugo 1.026 (1.016, 1.036)
Ourense 1.025 (1.015, 1.035)
Teruel 1.021 (1.005, 1.037)
Avila 1.020 (1.002, 1.038)
Leon 1.016 (1.006, 1.026)
Soria 1.022 (1.000, 1.045)
Zamora 1.025 (1.009, 1.042)
Subtotal 1.023 (1.018, 1.027)
20-23 (%)
A Coruña 1.009 (1.003, 1.015)
Salamanca 1.016 (1.004, 1.028)
Caceres 1.011 (0.999, 1.023)
Lleida 1.013 (1.003, 1.023)
Subtotal 1.011 (1.007, 1.015)
16-19 (%)
Pontevedra 1.017 (1.009, 1.025)
Alava 1.016 (1.000, 1.032)
Guipuzcoa 1.013 (1.003, 1.023)
Zaragoza 1.010 (1.002, 1.018)
Ciudad Real 1.015 (1.005, 1.025)
Guadalajara 1.017 (1.001, 1.033)
Toledo 1.016 (1.004, 1.028)
Badajoz 1.017 (1.007, 1.027)
Barcelona 1.006 (1.002, 1.010)
Cordoba 1.016 (1.008, 1.024)
Granada 1.008 (1.000, 1.016)
Jaen 1.018 (1.008, 1.028)
Subtotal 1.013 (1.010, 1.016)
11-15 (%)
Huelva 1.013 (1.001, 1.025)
Madrid 1.007 (1.003, 1.011)
Sevilla 1.008 (1.002, 1.014)
Subtotal 1.008 (1.005, 1.011)
Overall 1.014 (1.011, 1.016)
NOTE: Weights are from random effects analysis
.957
1
1.04

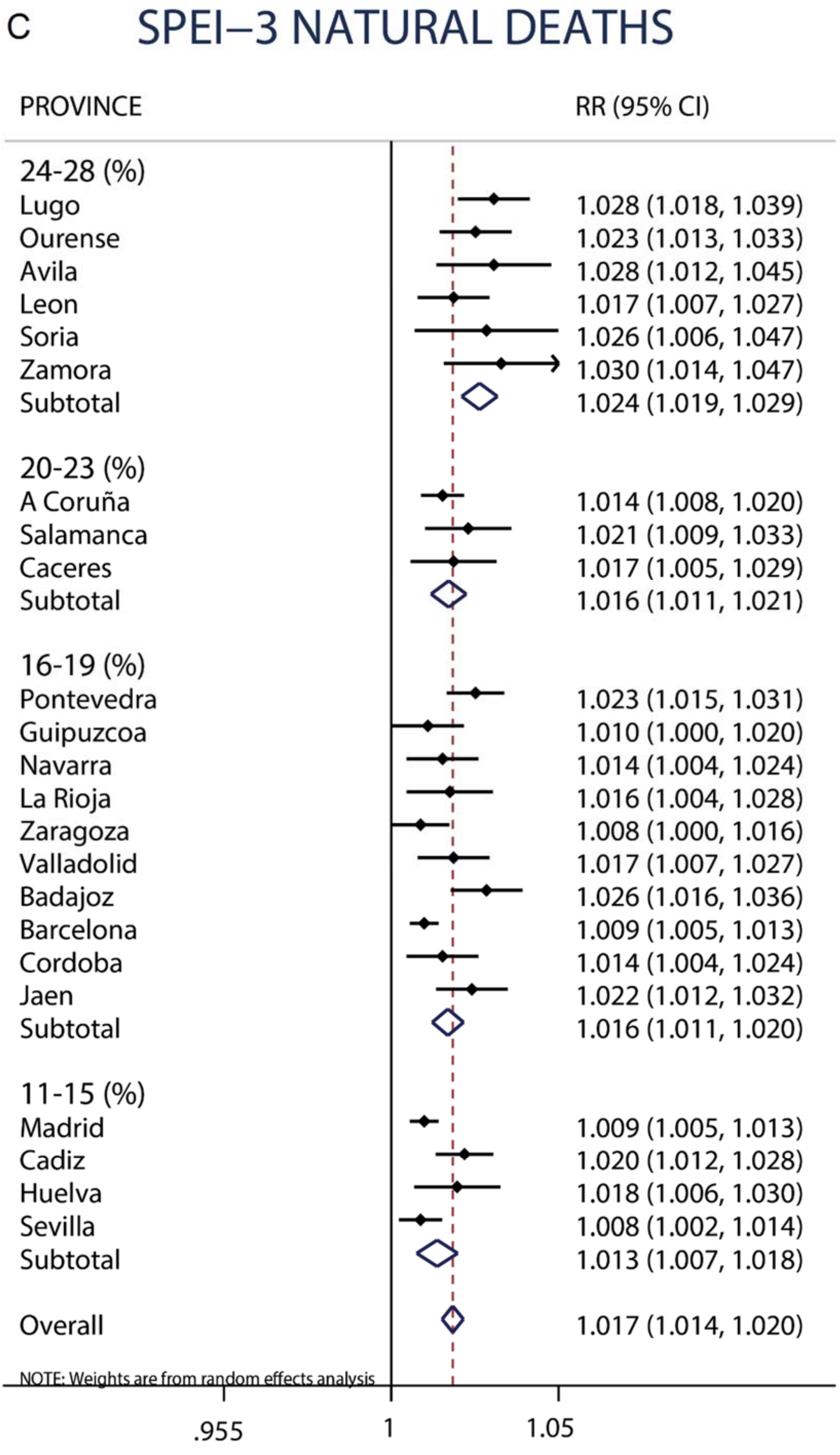
C
SPEI–3 NATURAL DEATHS
PROVINCE
RR (95% CI)
24-28 (%)
Lugo 1.028 (1.018, 1.039)
Ourense 1.023 (1.013, 1.033)
Avila 1.028 (1.012, 1.045)
Leon 1.017 (1.007, 1.027)
Soria 1.026 (1.006, 1.047)
Zamora 1.030 (1.014, 1.047)
Subtotal 1.024 (1.019, 1.029)
20-23 (%)
A Coruña 1.014 (1.008, 1.020)
Salamanca 1.021 (1.009, 1.033)
Caceres 1.017 (1.005, 1.029)
Subtotal 1.016 (1.011, 1.021)
16-19 (%)
Pontevedra 1.023 (1.015, 1.031)
Guipuzcoa 1.010 (1.000, 1.020)
Navarra 1.014 (1.004, 1.024)
La Rioja 1.016 (1.004, 1.028)
Zaragoza 1.008 (1.000, 1.016)
Valladolid 1.017 (1.007, 1.027)
Badajoz 1.026 (1.016, 1.036)
Barcelona 1.009 (1.005, 1.013)
Cordoba 1.014 (1.004, 1.024)
Jaen 1.022 (1.012, 1.032)
Subtotal 1.016 (1.011, 1.020)
11-15 (%)
Madrid 1.009 (1.005, 1.013)
Cadiz 1.020 (1.012, 1.028)
Huelva 1.018 (1.006, 1.030)
Sevilla 1.008 (1.002, 1.014)
Subtotal 1.013 (1.007, 1.018)
Overall 1.017 (1.014, 1.020)
NOTE: Weights are from random effects analysis
.955
1
1.05

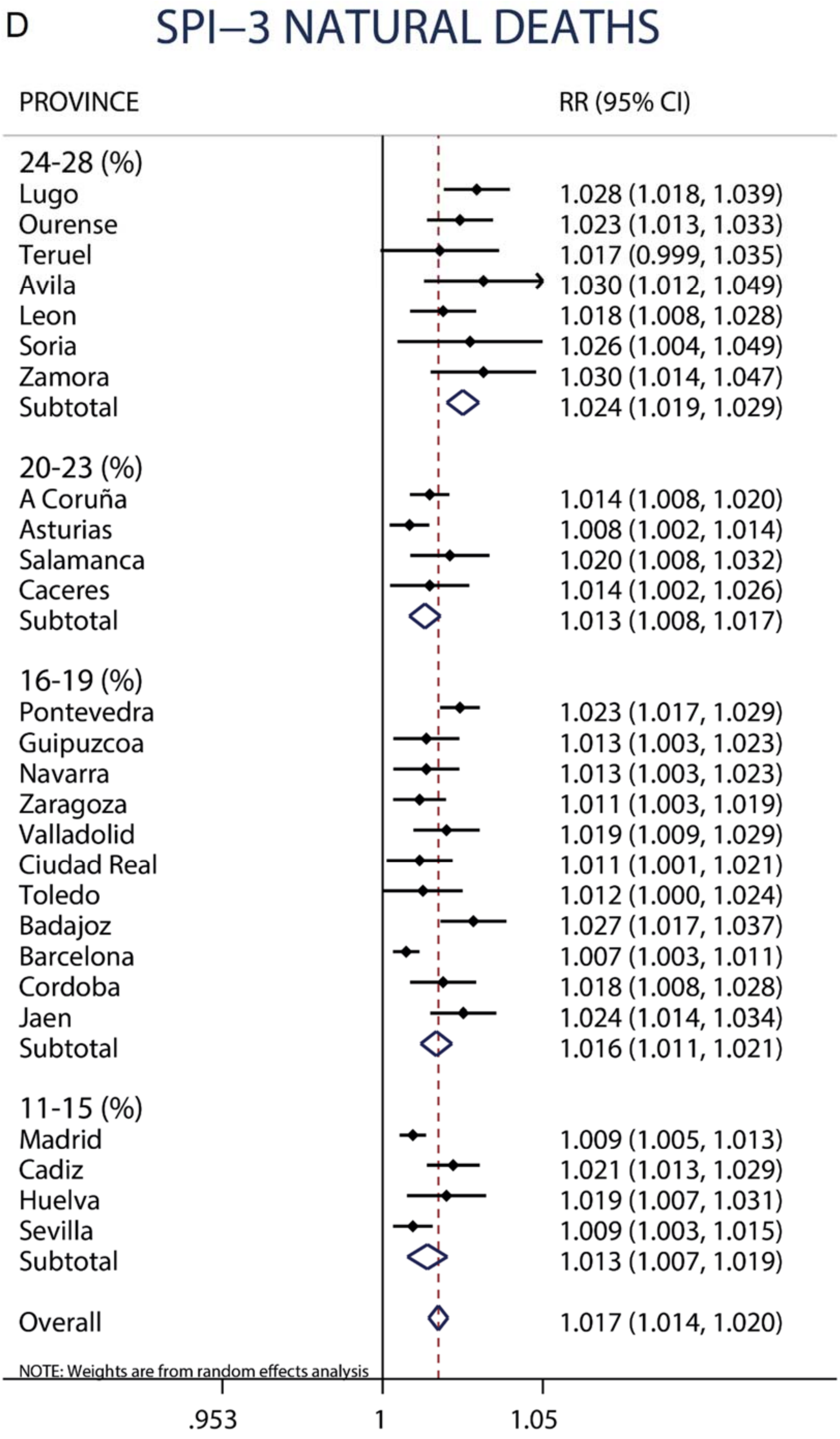
D
SPI–3 NATURAL DEATHS
PROVINCE
RR (95% CI)
24-28 (%)
Lugo 1.028 (1.018, 1.039)
Ourense 1.023 (1.013, 1.033)
Teruel 1.017 (0.999, 1.035)
Avila 1.030 (1.012, 1.049)
Leon 1.018 (1.008, 1.028)
Soria 1.026 (1.004, 1.049)
Zamora 1.030 (1.014, 1.047)
Subtotal 1.024 (1.019, 1.029)
20-23 (%)
A Coruña 1.014 (1.008, 1.020)
Asturias 1.008 (1.002, 1.014)
Salamanca 1.020 (1.008, 1.032)
Caceres 1.014 (1.002, 1.026)
Subtotal 1.013 (1.008, 1.017)
16-19 (%)
Pontevedra 1.023 (1.017, 1.029)
Guipuzcoa 1.013 (1.003, 1.023)
Navarra 1.013 (1.003, 1.023)
Zaragoza 1.011 (1.003, 1.019)
Valladolid 1.019 (1.009, 1.029)
Ciudad Real 1.011 (1.001, 1.021)
Toledo 1.012 (1.000, 1.024)
Badajoz 1.027 (1.017, 1.037)
Barcelona 1.007 (1.003, 1.011)
Cordoba 1.018 (1.008, 1.028)
Jaen 1.024 (1.014, 1.034)
Subtotal 1.016 (1.011, 1.021)
11-15 (%)
Madrid 1.009 (1.005, 1.013)
Cadiz 1.021 (1.013, 1.029)
Huelva 1.019 (1.007, 1.031)
Sevilla 1.009 (1.003, 1.015)
Subtotal 1.013 (1.007, 1.019)
Overall 1.017 (1.014, 1.020)
NOTE: Weights are from random effects analysis
.953
1
1.05

Figure S7. Forest plots of the relative risks (RR) values of daily natural mortality associated with droughts for provincial groups based on the proportion of elderly population in peninsular Spain.**A** and **B**: Droughts measured by the Standardized Precipitation Evapotranspiration Index (SPEI) and the Standardized Precipitation Index (SPI) obtained at one month of drought accumulation (SPEI-1 and SPI-1, respectively). **C** and **D**: As per **A** and **B**, but for three months of accumulation (SPEI-3 and SPI-3, respectively). Only provinces with a statistically significant association (p<0.05) between drought indices and natural deaths are shown. Provincial RR data obtained with the use of both SPEI-1 and SPI-1 from Salvador et al., 2020.

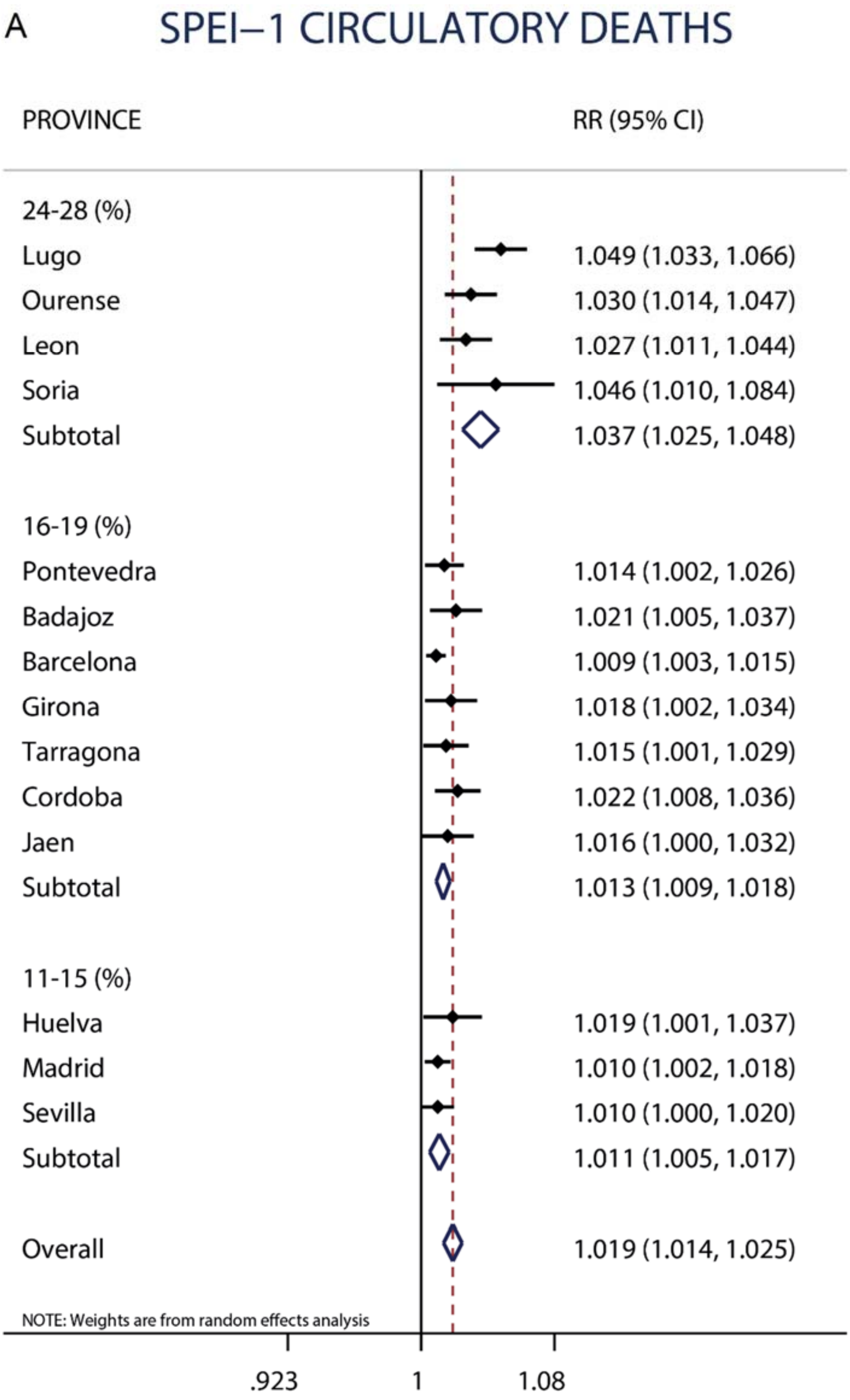
A
SPEI–1 CIRCULATORY DEATHS
PROVINCE
RR (95% CI)
24-28 (%)
Lugo
1.049 (1.033, 1.066)
Ourense
1.030 (1.014, 1.047)
Leon
1.027 (1.011, 1.044)
Soria
1.046 (1.010, 1.084)
Subtotal
1.037 (1.025, 1.048)
16-19 (%)
Pontevedra
1.014 (1.002, 1.026)
Badajoz
1.021 (1.005, 1.037)
Barcelona
1.009 (1.003, 1.015)
Girona
1.018 (1.002, 1.034)
Tarragona
1.015 (1.001, 1.029)
Cordoba
1.022 (1.008, 1.036)
Jaen
1.016 (1.000, 1.032)
Subtotal
1.013 (1.009, 1.018)
11-15 (%)
Huelva
1.019 (1.001, 1.037)
Madrid
1.010 (1.002, 1.018)
Sevilla
1.010 (1.000, 1.020)
Subtotal
1.011 (1.005, 1.017)
Overall
1.019 (1.014, 1.025)
NOTE: Weights are from random effects analysis
.923
1
1.08

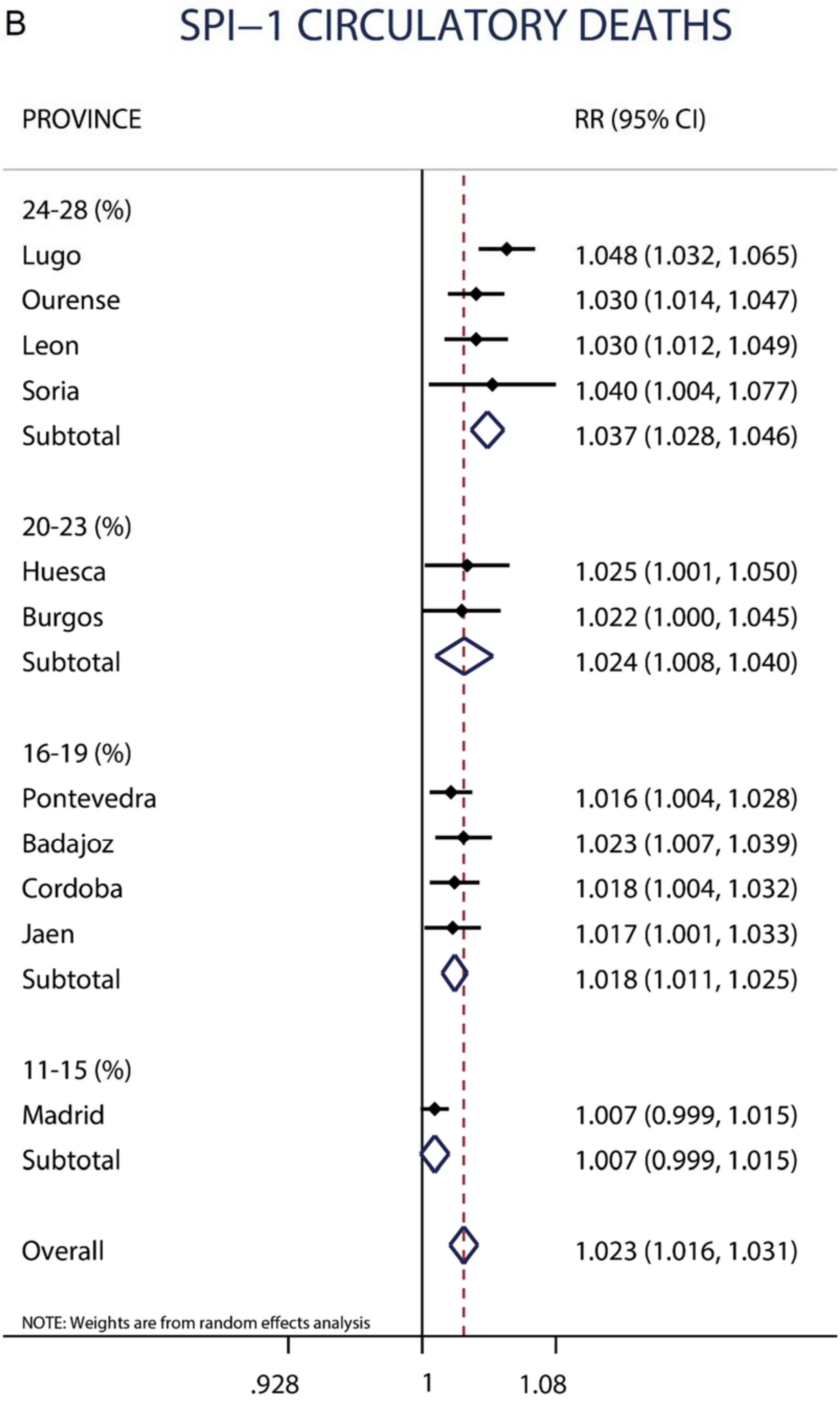
B
SPI–1 CIRCULATORY DEATHS
PROVINCE
RR (95% CI)
24-28 (%)
Lugo 1.048 (1.032, 1.065)
Ourense 1.030 (1.014, 1.047)
Leon 1.030 (1.012, 1.049)
Soria 1.040 (1.004, 1.077)
Subtotal 1.037 (1.028, 1.046)
20-23 (%)
Huesca 1.025 (1.001, 1.050)
Burgos 1.022 (1.000, 1.045)
Subtotal 1.024 (1.008, 1.040)
16-19 (%)
Pontevedra 1.016 (1.004, 1.028)
Badajoz 1.023 (1.007, 1.039)
Cordoba 1.018 (1.004, 1.032)
Jaen 1.017 (1.001, 1.033)
Subtotal 1.018 (1.011, 1.025)
11-15 (%)
Madrid 1.007 (0.999, 1.015)
Subtotal 1.007 (0.999, 1.015)
Overall 1.023 (1.016, 1.031)
NOTE: Weights are from random effects analysis
.928
1
1.08

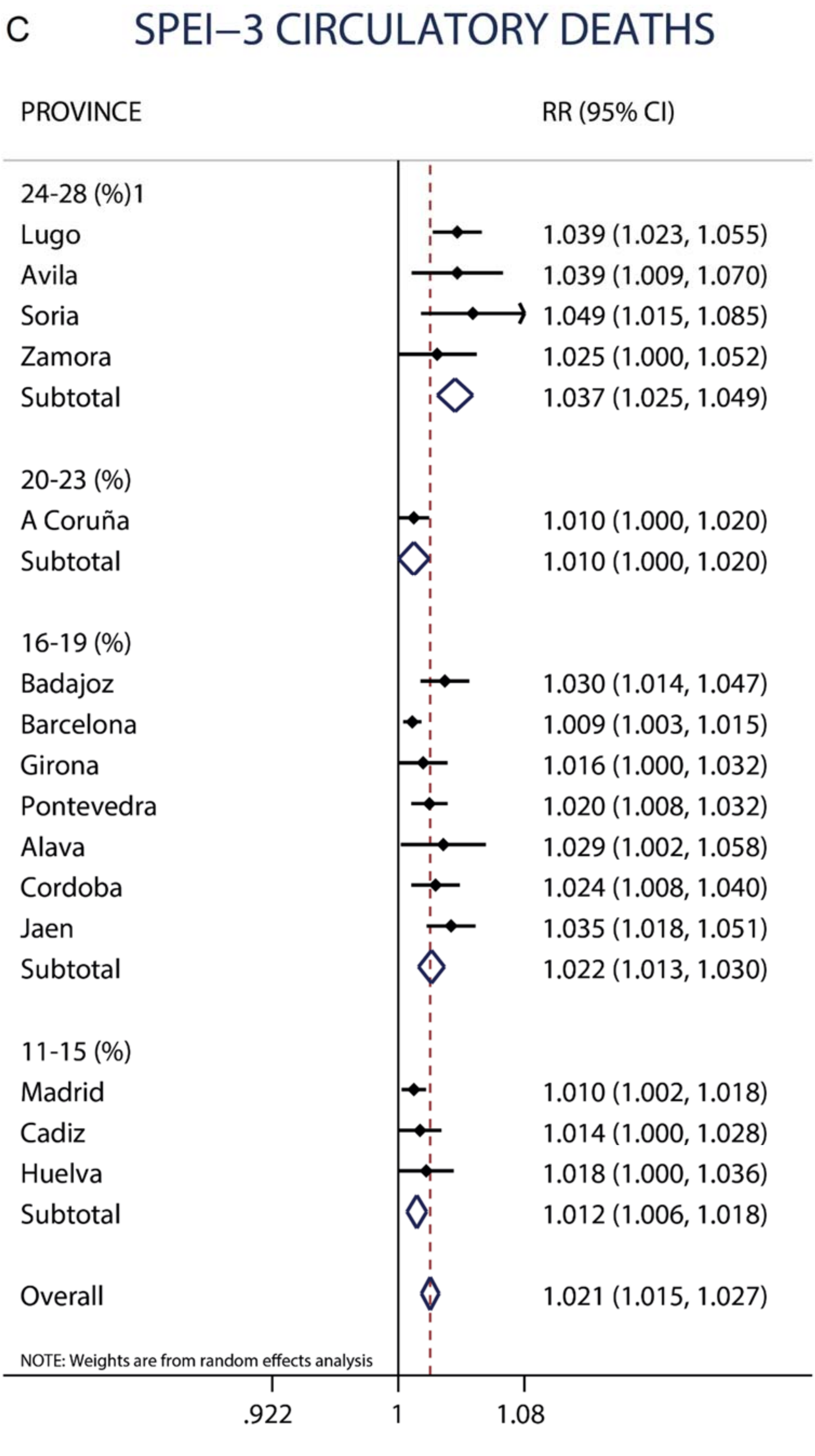
C
SPEI–3 CIRCULATORY DEATHS
PROVINCE
RR (95% CI)
24-28 (%)1
Lugo 1.039 (1.023, 1.055)
Avila 1.039 (1.009, 1.070)
Soria 1.049 (1.015, 1.085)
Zamora 1.025 (1.000, 1.052)
Subtotal 1.037 (1.025, 1.049)
20-23 (%)
A Coruña 1.010 (1.000, 1.020)
Subtotal 1.010 (1.000, 1.020)
16-19 (%)
Badajoz 1.030 (1.014, 1.047)
Barcelona 1.009 (1.003, 1.015)
Girona 1.016 (1.000, 1.032)
Pontevedra 1.020 (1.008, 1.032)
Alava 1.029 (1.002, 1.058)
Cordoba 1.024 (1.008, 1.040)
Jaen 1.035 (1.018, 1.051)
Subtotal 1.022 (1.013, 1.030)
11-15 (%)
Madrid 1.010 (1.002, 1.018)
Cadiz 1.014 (1.000, 1.028)
Huelva 1.018 (1.000, 1.036)
Subtotal 1.012 (1.006, 1.018)
Overall 1.021 (1.015, 1.027)
NOTE: Weights are from random effects analysis
.922
1
1.08

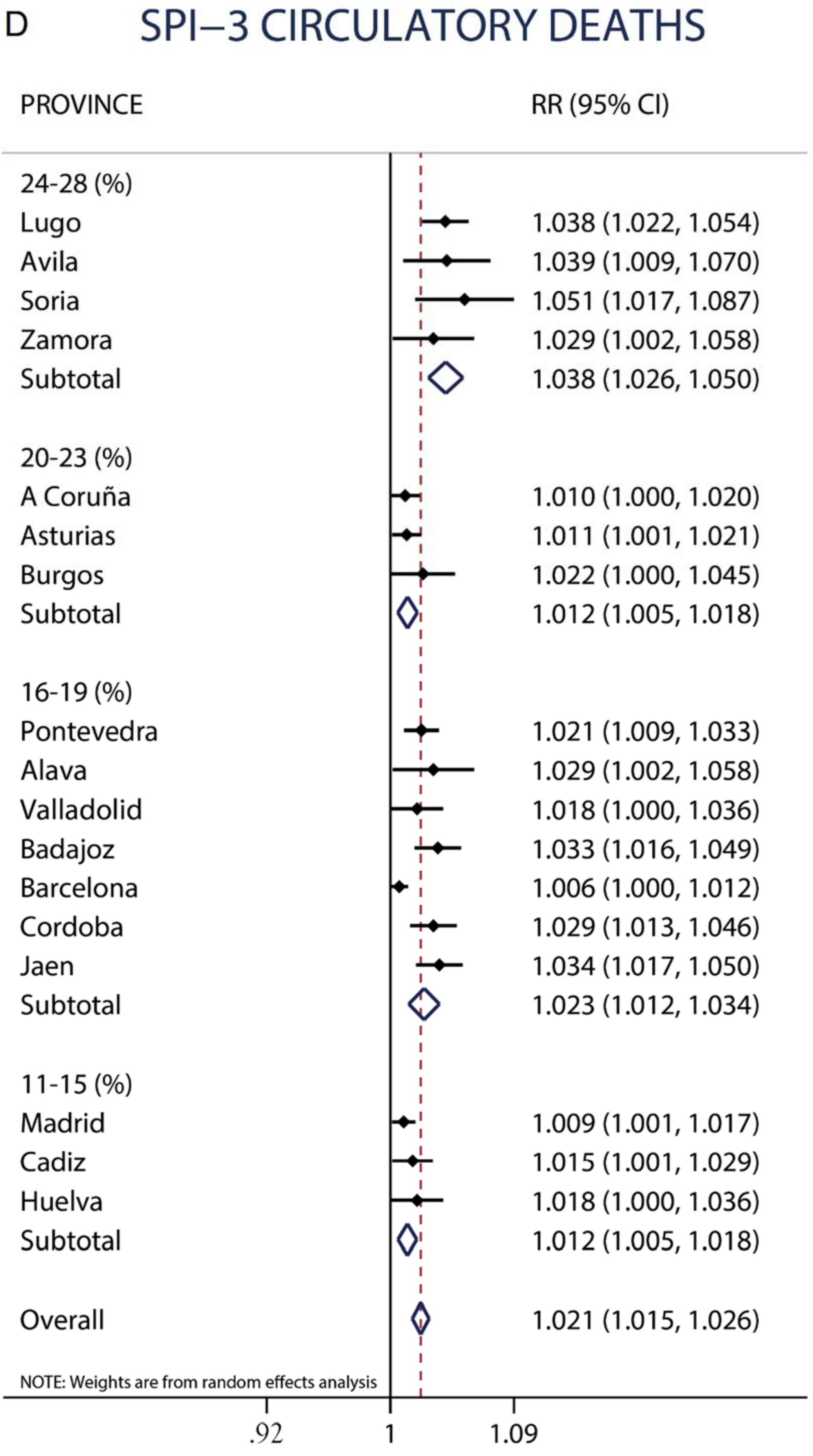
D
SPI–3 CIRCULATORY DEATHS
PROVINCE
RR (95% CI)
24-28 (%)
Lugo 1.038 (1.022, 1.054)
Avila 1.039 (1.009, 1.070)
Soria 1.051 (1.017, 1.087)
Zamora 1.029 (1.002, 1.058)
Subtotal 1.038 (1.026, 1.050)
20-23 (%)
A Coruña 1.010 (1.000, 1.020)
Asturias 1.011 (1.001, 1.021)
Burgos 1.022 (1.000, 1.045)
Subtotal 1.012 (1.005, 1.018)
16-19 (%)
Pontevedra 1.021 (1.009, 1.033)
Alava 1.029 (1.002, 1.058)
Valladolid 1.018 (1.000, 1.036)
Badajoz 1.033 (1.016, 1.049)
Barcelona 1.006 (1.000, 1.012)
Cordoba 1.029 (1.013, 1.046)
Jaen 1.034 (1.017, 1.050)
Subtotal 1.023 (1.012, 1.034)
11-15 (%)
Madrid 1.009 (1.001, 1.017)
Cadiz 1.015 (1.001, 1.029)
Huelva 1.018 (1.000, 1.036)
Subtotal 1.012 (1.005, 1.018)
Overall 1.021 (1.015, 1.026)
NOTE: Weights are from random effects analysis
.92
1
1.09

Figure S8. Forest plots of the relative risks (RR) values of daily circulatory mortality associated with droughts for provincial groups based on the proportion of elderly population in peninsular Spain. **A** and **B**: Droughts measured by the Standardized Precipitation Evapotranspiration Index (SPEI) and the Standardized Precipitation Index (SPI) obtained at one month of drought accumulation (SPEI-1 and SPI-1, respectively). **C** and **D**: As per **A** and **B**, but for three months of accumulation (SPEI-3 and SPI-3, respectively). Only provinces with a statistically significant association (p<0.05) between drought indices and circulatory deaths are shown. Provincial RR data obtained with the use of both SPEI-1 and SPI-1 from Salvador et al., 2020..

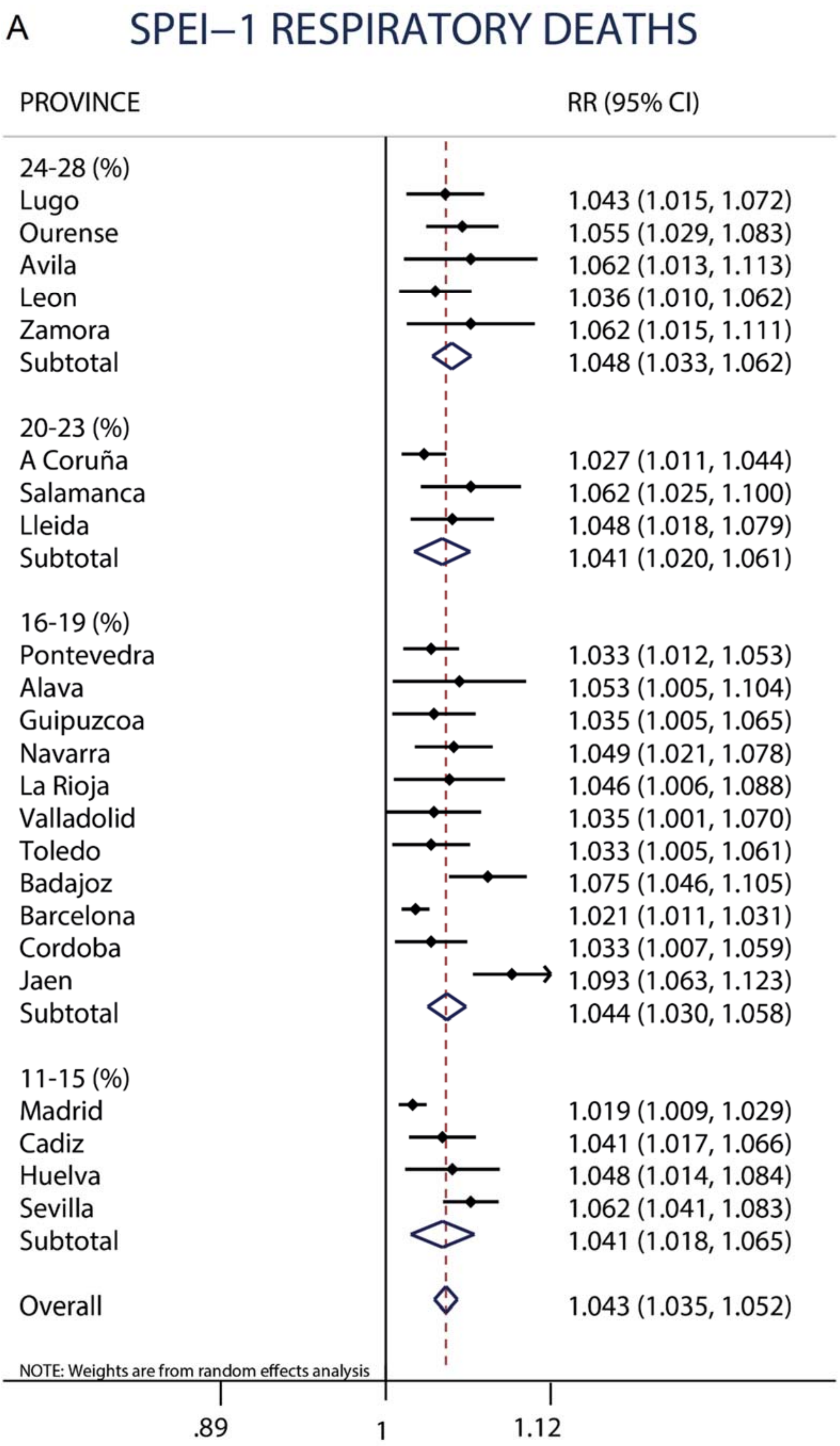
A
SPEI–1 RESPIRATORY DEATHS
PROVINCE
RR (95% CI)
24-28 (%)
Lugo 1.043 (1.015, 1.072)
Ourense 1.055 (1.029, 1.083)
Avila 1.062 (1.013, 1.113)
Leon 1.036 (1.010, 1.062)
Zamora 1.062 (1.015, 1.111)
Subtotal 1.048 (1.033, 1.062)
20-23 (%)
A Coruña 1.027 (1.011, 1.044)
Salamanca 1.062 (1.025, 1.100)
Lleida 1.048 (1.018, 1.079)
Subtotal 1.041 (1.020, 1.061)
16-19 (%)
Pontevedra 1.033 (1.012, 1.053)
Alava 1.053 (1.005, 1.104)
Guipuzcoa 1.035 (1.005, 1.065)
Navarra 1.049 (1.021, 1.078)
La Rioja 1.046 (1.006, 1.088)
Valladolid 1.035 (1.001, 1.070)
Toledo 1.033 (1.005, 1.061)
Badajoz 1.075 (1.046, 1.105)
Barcelona 1.021 (1.011, 1.031)
Cordoba 1.033 (1.007, 1.059)
Jaen 1.093 (1.063, 1.123)
Subtotal 1.044 (1.030, 1.058)
11-15 (%)
Madrid 1.019 (1.009, 1.029)
Cadiz 1.041 (1.017, 1.066)
Huelva 1.048 (1.014, 1.084)
Sevilla 1.062 (1.041, 1.083)
Subtotal 1.041 (1.018, 1.065)
Overall 1.043 (1.035, 1.052)
NOTE: Weights are from random effects analysis
.89
1
1.12

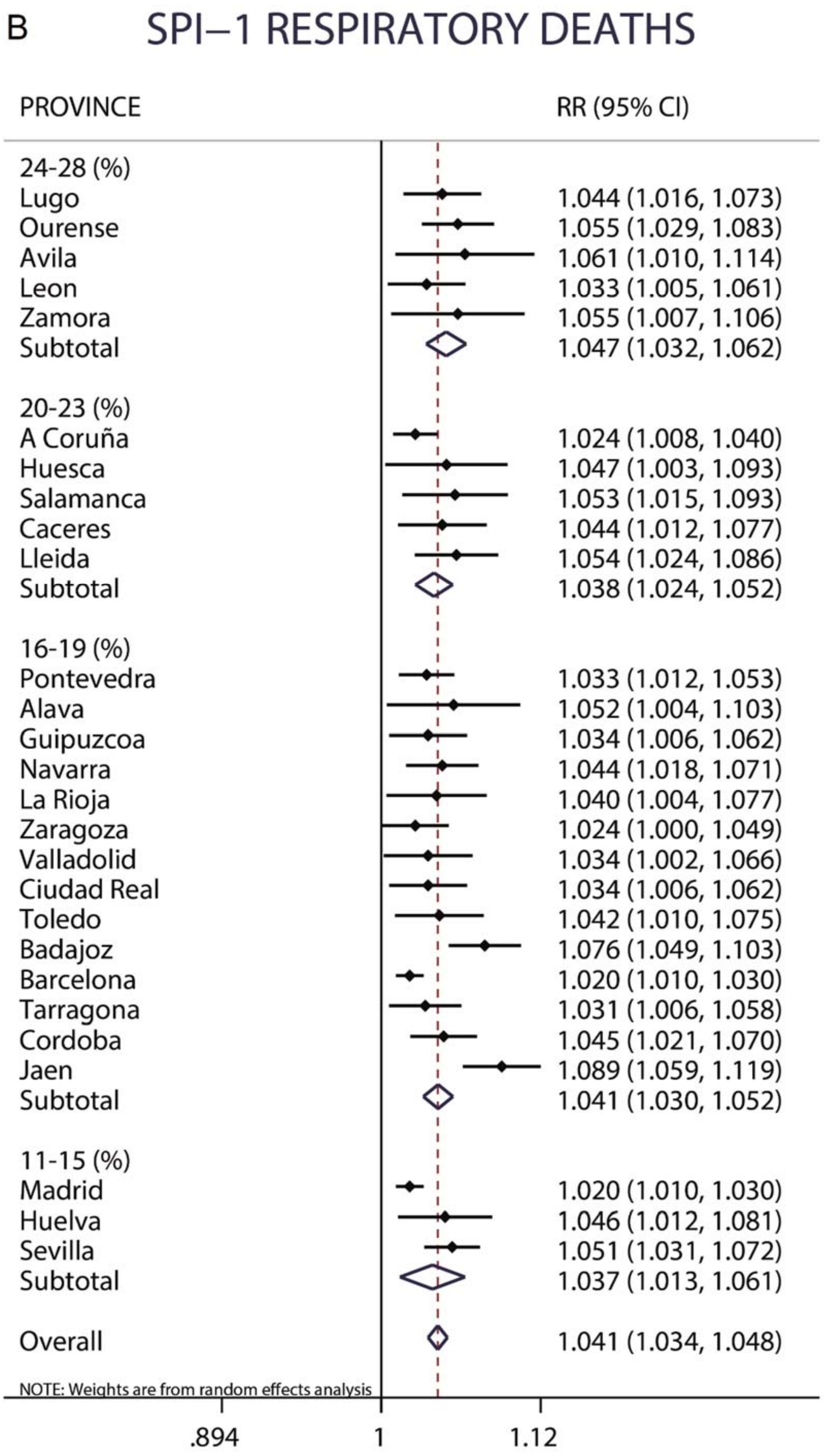
B
SPI–1 RESPIRATORY DEATHS
PROVINCE
RR (95% CI)
24-28 (%)
Lugo 1.044 (1.016, 1.073)
Ourense 1.055 (1.029, 1.083)
Avila 1.061 (1.010, 1.114)
Leon 1.033 (1.005, 1.061)
Zamora 1.055 (1.007, 1.106)
Subtotal 1.047 (1.032, 1.062)
20-23 (%)
A Coruña 1.024 (1.008, 1.040)
Huesca 1.047 (1.003, 1.093)
Salamanca 1.053 (1.015, 1.093)
Caceres 1.044 (1.012, 1.077)
Lleida 1.054 (1.024, 1.086)
Subtotal 1.038 (1.024, 1.052)
16-19 (%)
Pontevedra 1.033 (1.012, 1.053)
Alava 1.052 (1.004, 1.103)
Guipuzcoa 1.034 (1.006, 1.062)
Navarra 1.044 (1.018, 1.071)
La Rioja 1.040 (1.004, 1.077)
Zaragoza 1.024 (1.000, 1.049)
Valladolid 1.034 (1.002, 1.066)
Ciudad Real 1.034 (1.006, 1.062)
Toledo 1.042 (1.010, 1.075)
Badajoz 1.076 (1.049, 1.103)
Barcelona 1.020 (1.010, 1.030)
Tarragona 1.031 (1.006, 1.058)
Cordoba 1.045 (1.021, 1.070)
Jaen 1.089 (1.059, 1.119)
Subtotal 1.041 (1.030, 1.052)
11-15 (%)
Madrid 1.020 (1.010, 1.030)
Huelva 1.046 (1.012, 1.081)
Sevilla 1.051 (1.031, 1.072)
Subtotal 1.037 (1.013, 1.061)
Overall 1.041 (1.034, 1.048)
NOTE: Weights are from random effects analysis
.894
1
1.12

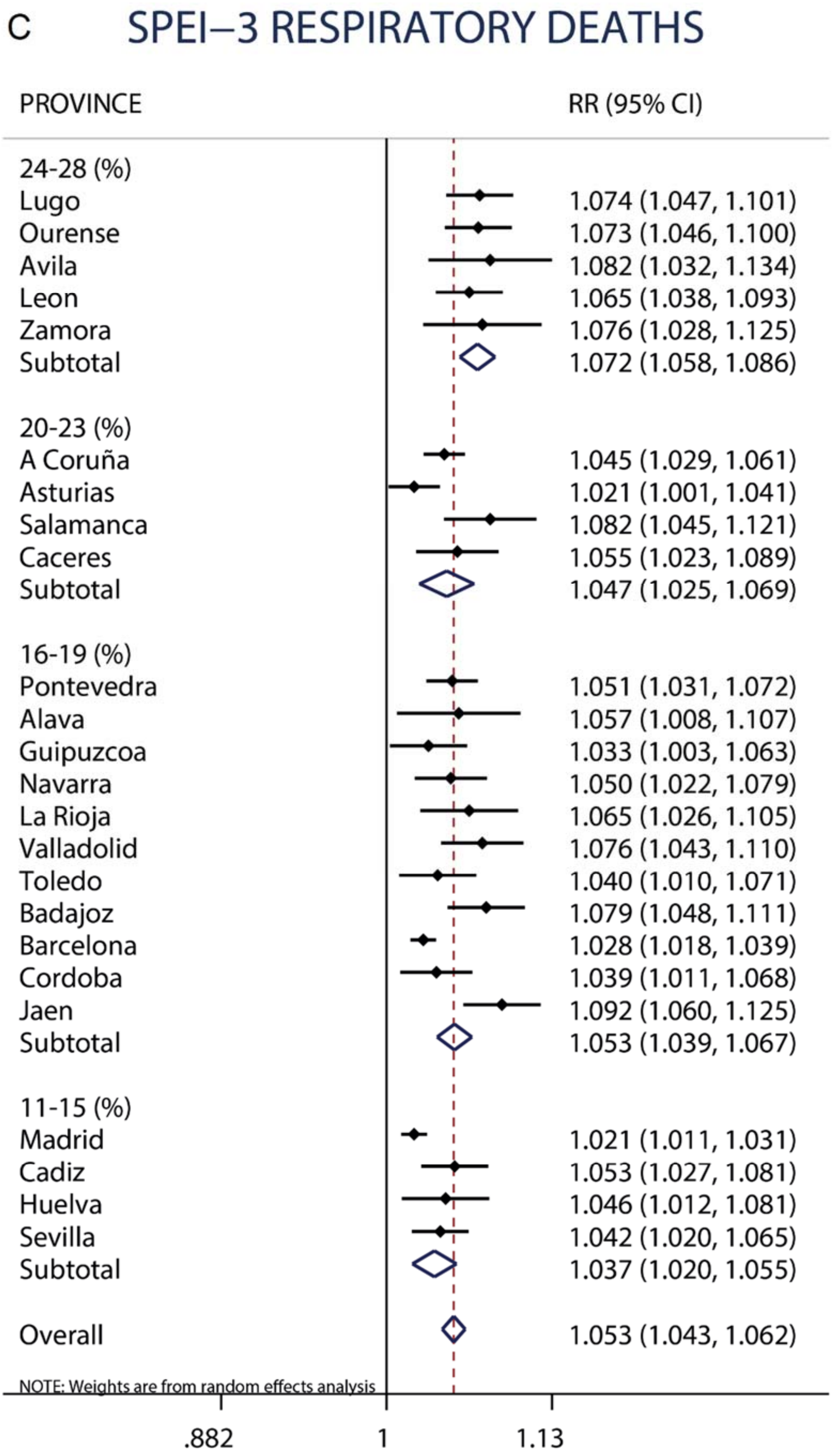
C
SPEI–3 RESPIRATORY DEATHS
PROVINCE
RR (95% CI)
24-28 (%)
Lugo
1.074 (1.047, 1.101)
Ourense
1.073 (1.046, 1.100)
Avila
1.082 (1.032, 1.134)
Leon
1.065 (1.038, 1.093)
Zamora
1.076 (1.028, 1.125)
Subtotal
1.072 (1.058, 1.086)
20-23 (%)
A Coruña
1.045 (1.029, 1.061)
Asturias
1.021 (1.001, 1.041)
Salamanca
1.082 (1.045, 1.121)
Caceres
1.055 (1.023, 1.089)
Subtotal
1.047 (1.025, 1.069)
16-19 (%)
Pontevedra
1.051 (1.031, 1.072)
Alava
1.057 (1.008, 1.107)
Guipuzcoa
1.033 (1.003, 1.063)
Navarra
1.050 (1.022, 1.079)
La Rioja
1.065 (1.026, 1.105)
Valladolid
1.076 (1.043, 1.110)
Toledo
1.040 (1.010, 1.071)
Badajoz
1.079 (1.048, 1.111)
Barcelona
1.028 (1.018, 1.039)
Cordoba
1.039 (1.011, 1.068)
Jaen
1.092 (1.060, 1.125)
Subtotal
1.053 (1.039, 1.067)
11-15 (%)
Madrid
1.021 (1.011, 1.031)
Cadiz
1.053 (1.027, 1.081)
Huelva
1.046 (1.012, 1.081)
Sevilla
1.042 (1.020, 1.065)
Subtotal
1.037 (1.020, 1.055)
Overall
1.053 (1.043, 1.062)
NOTE: Weights are from random effects analysis
.882
1
1.13

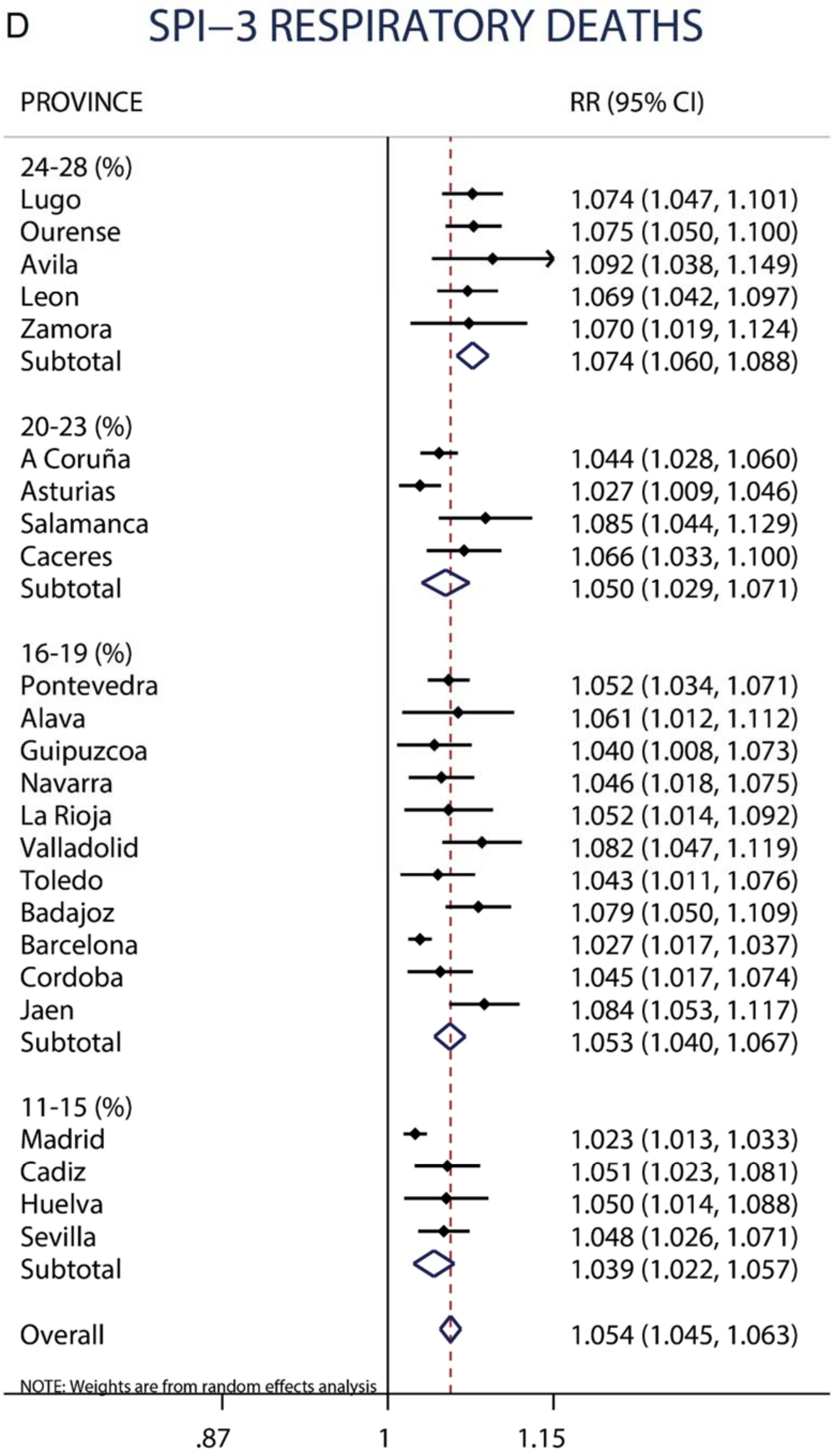
D
SPI–3 RESPIRATORY DEATHS
PROVINCE
RR (95% CI)
24-28 (%)
Lugo 1.074 (1.047, 1.101)
Ourense 1.075 (1.050, 1.100)
Avila 1.092 (1.038, 1.149)
Leon 1.069 (1.042, 1.097)
Zamora 1.070 (1.019, 1.124)
Subtotal 1.074 (1.060, 1.088)
20-23 (%)
A Coruña 1.044 (1.028, 1.060)
Asturias 1.027 (1.009, 1.046)
Salamanca 1.085 (1.044, 1.129)
Caceres 1.066 (1.033, 1.100)
Subtotal 1.050 (1.029, 1.071)
16-19 (%)
Pontevedra 1.052 (1.034, 1.071)
Alava 1.061 (1.012, 1.112)
Guipuzcoa 1.040 (1.008, 1.073)
Navarra 1.046 (1.018, 1.075)
La Rioja 1.052 (1.014, 1.092)
Valladolid 1.082 (1.047, 1.119)
Toledo 1.043 (1.011, 1.076)
Badajoz 1.079 (1.050, 1.109)
Barcelona 1.027 (1.017, 1.037)
Cordoba 1.045 (1.017, 1.074)
Jaen 1.084 (1.053, 1.117)
Subtotal 1.053 (1.040, 1.067)
11-15 (%)
Madrid 1.023 (1.013, 1.033)
Cadiz 1.051 (1.023, 1.081)
Huelva 1.050 (1.014, 1.088)
Sevilla 1.048 (1.026, 1.071)
Subtotal 1.039 (1.022, 1.057)
Overall 1.054 (1.045, 1.063)
NOTE: Weights are from random effects analysis
.87
1
1.15

Figure S9. Forest plots of the relative risks (RR) values of daily respiratory mortality associated with droughts for provincial groups based on the proportion of elderly population in peninsular Spain. **A** and **B**: Droughts measured by the Standardized Precipitation Evapotranspiration Index (SPEI) and the Standardized Precipitation Index (SPI) obtained at one month of drought accumulation (SPEI-1 and SPI-1, respectively). **C** and **D**: As per **A** and **B**, but for three months of accumulation (SPEI-3 and SPI-3, respectively). Only provinces with a statistically significant association (p<0.05) between drought indices and circulatory deaths are shown. Provincial RR data obtained with the use of both SPEI-1 and SPI-1 from Salvador et al., 2020.
